# Time space and single-cell resolved tissue lineage trajectories and laterality of body plan at gastrulation

**DOI:** 10.1038/s41467-023-41482-5

**Published:** 2023-09-14

**Authors:** Ran Wang, Xianfa Yang, Jiehui Chen, Lin Zhang, Jonathan A. Griffiths, Guizhong Cui, Yingying Chen, Yun Qian, Guangdun Peng, Jinsong Li, Liantang Wang, John C. Marioni, Patrick P. L. Tam, Naihe Jing

**Affiliations:** 1 State Key Laboratory of Cell Biology, CAS Center for Excellence in Molecular Cell Science, Shanghai Institute of Biochemistry and Cell Biology, Chinese Academy of Sciences, 320 Yue Yang Road, Shanghai, 200031 China; 2 Guangzhou National Laboratory, Guangzhou, 510005 Guangdong Province China; 3 Cancer Research UK Cambridge Institute, University of Cambridge, Cambridge, CB2 0RE UK; 4 European Molecular Biology Laboratory, European Bioinformatics Institute (EMBL-EBI), Cambridge, CB10 1SD UK; 5 CAS Key Laboratory of Regenerative Biology, Guangdong Provincial Key Laboratory of Stem Cell and Regenerative Medicine, Guangzhou Institutes of Biomedicine and Health, Chinese Academy of Sciences, Guangzhou, 510530 China; 6 Institute for Stem Cell and Regeneration, Chinese Academy of Sciences, Beijing, 100101 China; 7 School of Mathematics, Northwest University, Xi’an, 710127 China; 8 Embryology Research Unit, Children’s Medical Research Institute, University of Sydney, Sydney, New South Wales Australia; 9 School of Medical Sciences, Faculty of Medicine and Health, University of Sydney, Sydney, New South Wales Australia; 10 Present Address: Genomics Plc, 50-60 Station Road, Cambridge, CB1 2JH UK

**Keywords:** Gastrulation, Computational biology and bioinformatics, Morphogen signalling

## Abstract

Understanding of the molecular drivers of lineage diversification and tissue patterning during primary germ layer development requires in-depth knowledge of the dynamic molecular trajectories of cell lineages across a series of developmental stages of gastrulation. Through computational modeling, we constructed at single-cell resolution, a spatio-temporal transcriptome of cell populations in the germ-layers of gastrula-stage mouse embryos. This molecular atlas enables the inference of molecular network activity underpinning the specification and differentiation of the germ-layer tissue lineages. Heterogeneity analysis of cellular composition at defined positions in the epiblast revealed progressive diversification of cell types. The single-cell transcriptome revealed an enhanced BMP signaling activity in the right-side mesoderm of late-gastrulation embryo. Perturbation of asymmetric BMP signaling activity at late gastrulation led to randomization of left-right molecular asymmetry in the lateral mesoderm of early-somite-stage embryo. These findings indicate the asymmetric BMP activity during gastrulation may be critical for the symmetry breaking process.

## Abstract (web summary)

Gastrulation entails a series of events that are highly coordinated in space and time. Here they construct a spatiotemporal molecular atlas of lineage trajectories in the gastrulating mouse embryo by mapping single cells to spatial coordinates in the germ layers with reference to positional data in the transcriptome.

## Introduction

During gastrulation, the mouse embryo that is constituted initially of the epiblast and visceral endoderm is transformed into one with primary germ layers: ectoderm, mesoderm, and endoderm. The process of gastrulation encompasses the generation of diverse tissue lineages of the three germ layers and the assembly of the embryonic tissues into an early body plan. Fate-mapping and lineage tracing studies have shown that the epiblast and the primitive streak (PS) are the sources of ectoderm, mesoderm, and definitive endoderm^1–3^. The definitive endoderm, together with the contribution of the visceral endoderm, makes up the endoderm layer of the late-gastrulation embryo^4^. Cell populations in different regions of the epiblast, the PS, and the emerging germ layers display different prospective tissue fates, suggesting that a multitude of precursors of germ layer tissues may have been set aside during gastrulation and the precursors and derivatives are regionalized by tissue patterning^5–10^. Building on the knowledge of acquisition of prospective fates and regionalization of the cell populations in the germ layers, a better understanding of the mechanism and outcome of gastrulation would require a deeper insight into the developmental trajectory of the cell and tissue lineages and the genetic determinants and molecular drivers of lineage development and tissue patterning in the gastrulating embryo.

The molecular architecture of cell populations in mouse embryos at pre- to late-gastrulation stages has been charted by profiling the transcriptome of small groups of cells captured from defined locations in the germ layers^11^. These data have provided insight into the developmental trajectory of the germ layer tissues, the activity of the molecular networks associated with the transition of pluripotency states, and the specification and regionalization of the germ layer derivative during gastrulation. Since the transcriptomic profile was collated from populations of cells, it is not feasible to resolve the divergence in transcriptome activity between cells of different identities within each population. Recently, single-cell genomics and transcriptomics have been applied to investigate the molecular attributes and potential lineage relationship of the multitude of cell types in the embryo during development^12,13^. However, profiling the population of single cells pooled from embryos, where spatial information is lost, and analyzing cells from embryos at one developmental time point cannot provide insight into the molecular architecture of individual cells that are regionalized to the specific compartment (space) in the embryo across the developmental stages (time). It is, therefore, imperative to combine existing spatially resolved transcriptome information with single-cell transcriptomes to construct a high-dimension, in time and space (4D), molecular atlas of spatially- and stage-resolved single-cell gene expression profiles of cells in the germ layers of the embryo.

In this work, we constructed the 4D molecular roadmap of the developmental trajectory of cell populations in the germ layer of gastrulating mice, which enables inferring the molecular drivers of the specification and differentiation of diverse tissue lineages and gleaning new understanding of the morphogenetic activity underpinning embryonic patterning during mouse gastrulation.

## Results

### The spatiotemporal molecular atlas

A key prerequisite for the construction of the atlas of molecular trajectories of cells in the embryo is the collation of a stage- and spatially-registered population-based transcriptome dataset. To this end, we have enriched the transcriptome data of mouse embryonic day (E) 6.5–7.5 embryos by capturing two additional time points: E6.75 and E7.25, and integrating the data into the established spatio-temporal transcriptome^11^. The full transcriptome dataset comprises Geo-seq data of 29 (E6.5), 29 (E6.75), 73 (E7.0), 94 (E7.25), and 81 (E7.5) positionally registered samples from the epiblast, ectoderm, mesoderm, and endoderm of embryos (in replicates, Supplementary Fig. 1a–c). The data were rendered digitally for depiction in 2D corn plots, with the samples staged by the developmental status of the PS (by *T* expression) and the ectoderm progenitor (*Otx2* expression) (Supplementary Fig. 1d). Quality assessment of this dataset showed that a median of about 11,000 genes was detected per sample across all embryos (Supplementary Fig. 1e, f), with an average of 10 million reads per library that ensured sufficient sequencing depth saturation (Supplementary Fig. 1g and Supplementary Data 1 and 2).

**Fig. 1:**
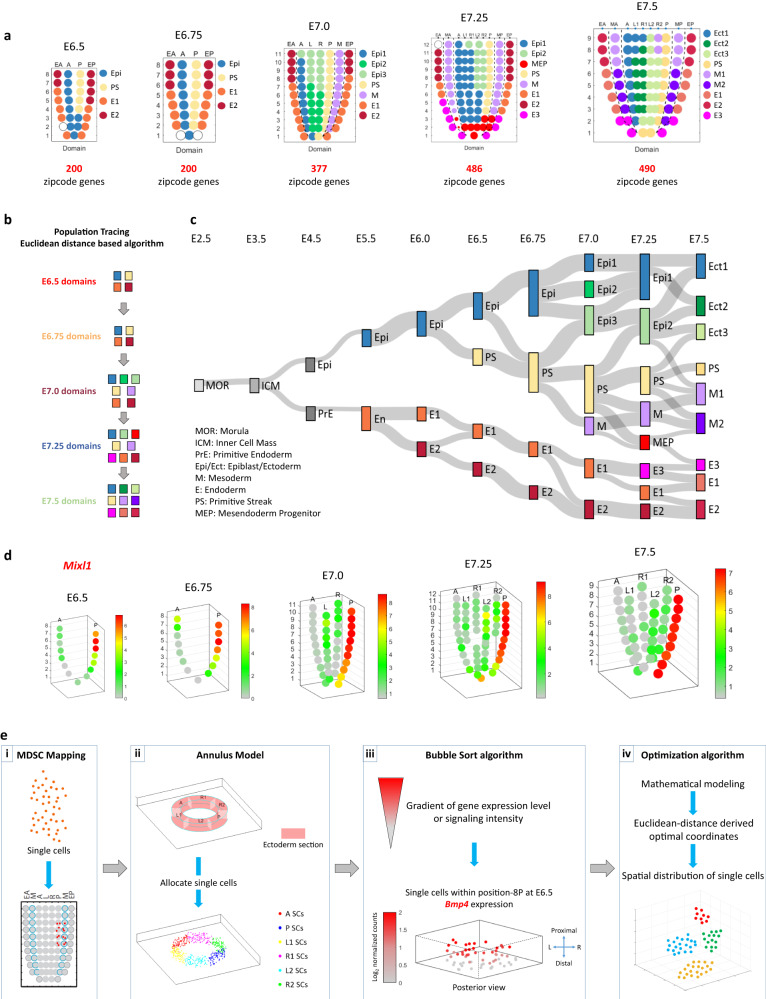
Generation of a spatio-temporal molecular atlas of the germ layers of gastrula-stage mouse embryo. **a** Spatial domain of cell populations in the epiblast/ectoderm, mesoderm, and endoderm of E6.5–E7.5 embryos, defined by the position-specific expression of zipcode gene transcripts. Geo-seq sampling positions: epiblast/ectoderm—A, anterior; L, left lateral; R, right lateral; L1/R1, left/right anterior lateral, L2/R2, left/right posterior lateral; M, mesoderm—MA, anterior mesoderm; MP, posterior mesoderm; E, endoderm—EA, anterior endoderm; EP, posterior endoderm. Number: descending series indicating positions in the proximal-distal axis. Germ layer domains: Epi: epiblast, Epi1, 2, 3: epiblast domain 1, 2, and 3; M: mesoderm, M1, M2: mesoderm domain 1 and 2; MEP, putative mesendoderm progenitors; E: endoderm, E1, E2, E3: endoderm domain 1, 2, and 3; PS, primitive streak. **b** The structure of the Population Tracing algorithm for imputing the developmental connectivity of cell populations across stages of gastrulation (see the detail of mathematical operations in Methods and Supplementary Fig. 2f). **c** The developmental trajectory of sub-populations within each germ layer tissue domain descending from blastomeres of the preimplantation E2.5 morula stage embryo to the germ layers of E7.5 late-gastrulation stage embryo. **d** 3D Model of the epiblast/ectoderm displaying the cell populations by imputed positional coordinates (see Methods for details of the mathematical modeling). The exemplar 3D corn plots show the spatiotemporal distribution of the *Mixl1*-expressing population in the primitive streak; the proximal-distal span of the *Mixl1*+ domain defines the developmental stage of the gastrulating embryo. The color legend indicates the level of expression determined by the transcript counts. **e** A flow diagram of the 4-step spatial mapping protocol. (1) Multi-Dimension Single-Cell (MDSC) Mapping allocates single cells to their imputed position. (2) The Annulus Model simulates the Geo-seq positions. Single cells that mapped to a Geo-seq position were distributed uniformly across the interior space of the position in each annulus section. (3) Bubble Sort algorithm displays the cells in relation to the gradient of gene expression level or signaling intensity. (4) The optimization algorithm refines and visualizes the spatial distribution pattern of cell types by optimal coordinates at each Geo-seq position.

The spatiotemporal transcriptome can be mined to identify the position-specific signature gene transcripts of cell populations in different domains of the germ layers of the embryos. Applying BIC-SKmeans and PC-loading analysis, the number of distinct gene-expression domains (Supplementary Fig. 2a–e) and the position-specific signature genes for corn plot (i.e., Geo-seq) positions (Fig. 1a; designated as the zipcode genes, zipcodes for short)^14–16^ were identified in the germ layers of E6.5–E7.5 embryos (see “Methods”). To trace the developmental connectivity of cell populations in various domains, we devised a Population Tracing algorithm (Fig. 1b, Supplementary Fig. 2f, and “Methods”) for inferring the putative molecular trajectory of cell populations at successive developmental stages. The algorithm was applied by collating the zipcodes of cell populations at different developmental stages as the input gene set and calculating the Euclidean distance of any two Geo-seq domains in embryos of successive developmental stages to identify the most closely-connected domains. Using a combined dataset of the transcriptome of pre- and peri-implantation embryo^17^ and the E5.5–E7.5 embryo^11^, we charted the developmental trajectory of cell populations from blastomeres of the morula to the nine major germ layer tissue domains at E7.5 (Fig. 1c and Supplementary Data 3).

The developmental trajectory has also provided insights into the location of unique progenitor cell types and the molecular activity that may be related to their ontogeny and differentiation. For example, through PC loading analysis, a mixed cell population containing putative mesendoderm progenitor (MEP) (Fig. 1a) was pinpointed to the E7.25 anterior PS and the adjacent mesoderm by the expression of Group (G)-5 and G-7 genes of the indicative functional ontogeny group (Supplementary Fig. 2d, g, h). By E7.5, the mixed population of dual potential precursor cells diverged into two cell types, the distal mesoderm (M2) and distal endoderm (E3) (Fig. 1c). Another example arose from tracking the expression of signature genes is the emerging node that is expressing genes of the top functional ontogeny of MEP (Supplementary Fig. 2e, g, i), that are characteristics of the gastrula organizer^18^. We also identified two cell populations in the proximal-lateral ectoderm (at positions: 8R2 and 9R2) of the E7.5 embryo (Supplementary Fig. 2e) that showed significant connectivity of transcriptome features (gene group G-8) with the mesoderm in the proximal region (M1, position 7-9MA/P) (see details later). Overall, the definition of spatial domains has a high inter-embryo consistency (Supplementary Fig. 3a–e), implicating significant synchronicity in the patterning of cell populations in the germ layers during gastrulation.

To recapitulate the topography of the embryo and visualize the spatial pattern of gene expression, the spatio-temporal transcriptome data were then rendered digitally and depicted in “3D corn plots” (Fig. 1d, Supplementary Fig. 4a and “Methods”), each dot in the 3D model represents the cell population at the specific positional address. The ‘3D corn plot’ model was utilized as the template for spatial mapping of single cells (see next section).

### A single-cell resolution 3D molecular atlas

Single-cell RNA-sequencing (scRNA-seq) approaches have been used to profile the molecular features of individual cells during early development^12,13^. However, most single-cell studies have profiled dissociated populations of cells, where spatial information of the single cells in the embryo is lost. Previous works of mapping the location of cells in biological structures on the basis of the concordance of the gene expression profile^15,19^ have been confounded by mathematical uncertainties and false positives^20,21^. A high-value attribute of the spatio-temporal transcriptome is the amenability of mining the dataset to identify population-specific signature transcripts as zipcodes (Fig. 1a) that could be applied for imputing the position of single cells in the germ layers of E6.5–E7.5 embryos^15,22^. Leveraging our spatio-temporal molecular atlas and single-cell RNA-sequencing (scRNA-seq) dataset, we developed an analytics methodology that comprises tiered algorithms to infer the spatial distribution of single cells and to reconstruct a single-cell resolution 3D molecular atlas (Fig. 1e).

**Fig. 2:**
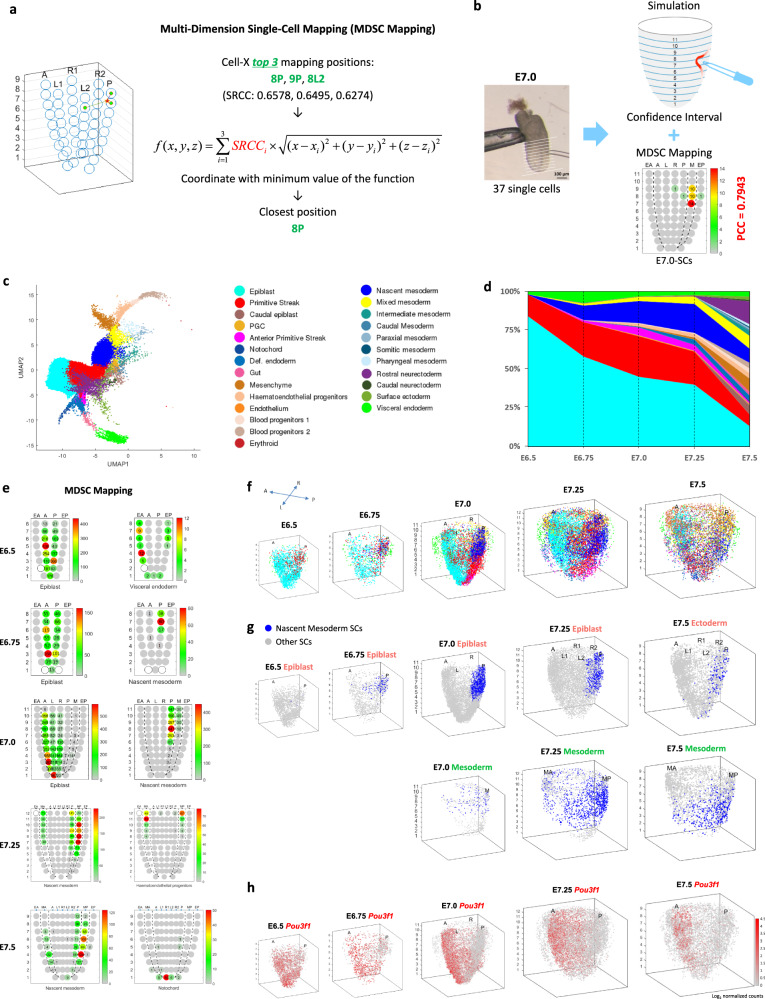
A single-cell resolution 4D molecular atlas of mouse gastrulation. **a** The pipeline of multi-dimension single-cell (MDSC) mapping. The SRCCs of the expression values of the zipcodes of each single cell against all reference samples of the reference embryo were calculated, followed by the application of a spatial smoothing algorithm to impute the high-confidence (closest) location (see “Methods”). The mapping of cells to position 8P was shown as an example. **b** Verification of the results of MDSC Mapping of single cells isolated from a known position in an E7.0 embryo. The number on each corn indicates the number of cells mapped to the Geo-seq position in the germ layers. PCC values and confidence intervals are shown for the simulation. **c** Uniform manifold approximation and projection (UMAP) plot showing the data structure of the “Gastrulation Atlas” comprising 32,940 cells from E6.5 to E7.5 embryos, with the exclusion of extraembryonic cells. Twenty-five cell types are annotated (see color legend). Def. endoderm definitive endoderm, PGC primordial germ cells. **d** Fraction of cell type per time point, displaying a progressive increase in cell-type complexity during gastrulation. **e** MDSC Mapping results of exemplar cell types for E6.5, E6.75, E7.0, E7.25, and E7.5 embryos. The number in each corn indicates the number of cells mapped to the specific Geo-seq position. **f** The spatiotemporal distribution of all the single cells identified in the Gastrulation Atlas of E6.5–E7.5 mouse embryos. Cell types are annotated as in (**c**). **g** The spatiotemporal distribution of single cells annotated as “nascent mesoderm” in the epiblast and mesoderm of E6.5–E7.5 embryos. **h** The spatio-temporal distribution of *Pou3f1*-expressing cells in E6.5–E7.5 embryos. The color legend indicates the level of expression determined by the transcript counts.

In our mapping study, the 3D corn plot template was used for mapping the spatial coordinates of single cells. By employing a multi-dimension single-cell mapping (MDSC Mapping) algorithm (Fig. 2a and “Methods”), single cells were mapped by the zipcodes embedded in their transcriptome to the inferred position in the model. To evaluate the level of precision of positional mapping, we tested the mapping of single cells isolated from known positions at five developmental time points between E6.5 and E7.5. The results showed that the single cells could be mapped to their best-fit site of origin at a significant confidence level (PCC values at 0.74–0.97, Fig. 2b, Supplementary Fig. 4b and “Methods”). Other technologies, such as MERFISH and MERSCOPE, are amenable for capturing spatial transcriptome at single-cell resolution but have limited gene detection rate^20^ compared with Geo-seq and therefore are less suitable for the spatial mapping of single cells.

Drawing from the single-cell transcriptome data of mouse embryos at gastrulation to early organogenesis (“Gastrulation atlas,” Fig. 2c, d)^12^, we first applied the MDSC Mapping algorithm and mapped the 25 embryonic cell types to the germ layers of the E6.5–E7.5 embryos (Fig. 2e and Supplementary Fig. 4c–l). To visualize the spatial distribution of the cells in the germ layers, an Annulus Model was applied to display the “Geo-seq position” of single cells (Fig. 1e, Supplementary Fig. 5a–e and Methods) in a series of 3D-rendered spatiotemporal maps. After mapping single cells to the best-fit position in the Annulus Model (Supplementary Fig. 5d, e), we applied mathematical modeling based on Bubble Sort Algorithm to re-position single cells within each Geo-seq position by incorporating information of vectorially graded molecular activity (e.g., gradient of signaling activity and gene expression levels) (Fig. 1e, Supplementary Fig. 5f and “Methods”) that is known to associate with the regionalization of cell fate. This mathematical model enables an effective imputation of the coordinates of single cells in the Geo-seq zipcode position (Fig. 2f–h). By taking into consideration the pattern of distribution across the developmental stage, the data could be rendered digitally into the spatio-temporal (4D) Atlas at single-cell resolution (Fig. 2f and Supplementary Fig. 6a). This 4D Atlas provides unique insights into the spatial distribution of, for example, specific group of single cells in the germ layers of E6.5–E7.5 embryos (Fig. 2g and Supplementary Fig. 6a) and, in specific cases, the *Pou3f1*-expressing cells in the epiblast/ectoderm (Fig. 2h), and T-expressing cells in the three germ layers (Supplementary Fig. 6b) during gastrulation.

The single-cell mapping results were generally consistent with prior knowledge of the identity of cell types annotated based on expression markers and lineage inference^11,12^. For example, the anterior PS cells were mapped to the distal posterior epiblast (positions 1P–4P) at E7.0 (Supplementary Fig. 4h), and rostral neuroectoderm cells were mapped to anterior regions of the ectoderm of E7.5 embryo (Supplementary Fig. 4l). However, some cell types were mapped to areas of the germ layer that are apparently inconsistent with their cell identity. For example, cells of the PS were mapped widely in the epiblast outside the PS (Supplementary Fig. 4d, f, h, j, l), and cells that are annotated as nascent mesoderm were mapped predominantly to the posterior epiblast (6P–11P), besides in the mesoderm layer at E7.0 (Supplementary Fig. 4h). It might be that these cells in the epiblast represent a transitional cell state and not the fully specified cell type^23,24^. This observation raised the possibility that the PS cells and nascent mesoderm mapped to the epiblast may represent a population of progenitor cells at the transition of their allocation from the epiblast to the emerging germ layers. Drawing on the knowledge of the prospective fate of cells in the epiblast of gastrulating embryos^1,11^, we re-annotated the single cells mapped to the epiblast as progenitor/precursor cells (the intermediate cell types) of the germ layer derivatives based on the findings of heterogeneity of cell populations at a coordinate position in the germ layers (see next section).

### Heterogeneity of space-registered cell population

**Fig. 3:**
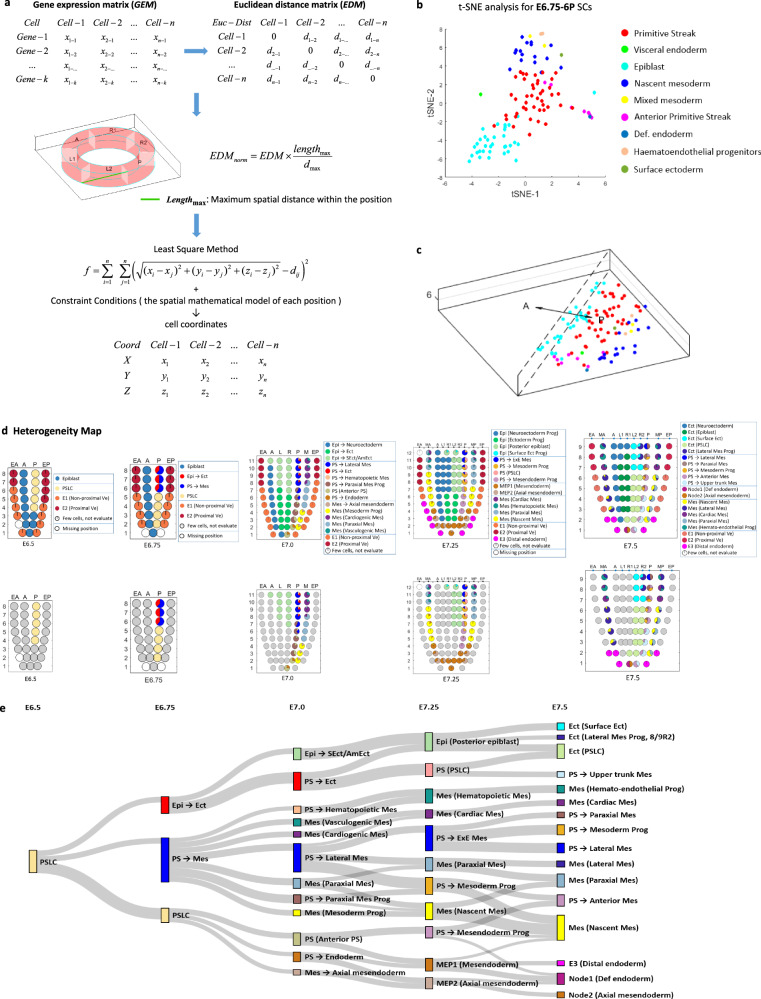
Mathematical modeling for single-cell spatial distribution and the collation of spatio-temporal heterogeneity map. **a** The mathematical model for re-ordering the spatial distribution of the single cells within a Geo-seq position by Euclidean-distance derived optimal coordinates (see Methods). **b** *t*-distributed stochastic neighbor embedding (*t*-SNE) plot showing the single cells mapped to position-6P at E6.75. Cell types are annotated (see legend). **c** The imputed spatial distribution of single cell types within position-6P at E6.75. Cell types are annotated as in (**b**). **d** Heterogeneity Map. Corn plots (top row) show the composition of cell types (shown as a pie chart in each corn) at different Geo-seq positions in the germ layers across the five-time points of gastrulation. Corn plots (bottom row) show the heterogeneity of cell populations descending from the primitive steak-like cells in different Geo-seq positions in the germ layers of the gastrulation stage embryo. **e** The molecular trajectory of the descendants of primitive streak-like cells of the E6.5 posterior epiblast in the germ layers of E6.75, E7.0, E7.25, and E7.5 embryo, imputed using the Population Tracing algorithm. Cell types are listed in Supplementary Fig. 8. The rectangle represents the spatialized cell type (color indicating the cell types), with the size indicating the propensity of branching trajectory, and the width of the edge indicates the strength of correlation between the connected cell types.

To visualize the composition of the single-cell population at each Geo-seq position, an optimization algorithm based on Euclidean distance was applied (Fig. 3a and “Methods”). Using the gene-expression matrix of single cells mapped to a specific position as an input, computing a normalized Euclidean distance matrix followed by applying the least square method, an optimized coordinate was assigned to each single cell. Applying this optimization algorithm, we simulated the spatial pattern of cell types within each Geo-seq position. For example, for position-6P at E6.75, this refined mapping revealed the presence of a heterogeneous cell population of the epiblast, PS, and nascent mesoderm cells (Fig. 3b, c). This finding prompted us to examine the heterogeneity of cell types in all the Geo-seq positions in the germ layers of the embryo at the five developmental time points.

To uncover the heterogeneity of single-cell population in the Geo-seq position, we applied t-SNE to re-cluster cells at each position in the germ layers of embryos at the five developmental stages (Supplementary Fig. 7). This analysis revealed that some cell types that were previously annotated differently were transcriptionally similar, illustrating the challenges of grouping of cells into discrete entities by primarily transcriptome. For these cells with different annotations that mapped to an unexpected germ-layer domain but displayed proximity of transcriptome, we re-annotated the cell identity based on both marker gene expression and prior knowledge of the prospective cell fate gleaned from fate-mapping and lineage tracing studies^25,26^ (Supplementary Figs. 7 and 8). For example, analysis of the heterogeneity of cell types identified two clusters of single cells at Geo-seq position-6P in the posterior epiblast of the E6.75 embryo (Supplementary Fig. 9a–c). One cluster displayed upregulation of mesoderm genes and enriched gene ontology (GO) terms of mesoderm development, and the other cluster (Cluster 2) displayed a transcription signature of ectoderm. These two clusters were therefore annotated as ‘PS→Mes’ and ‘Epi→Ect’ respectively (Supplementary Fig. 9c, see methods for nomenclature). RNAscope analyses validated the expression of *Mesp1* (Supplementary Fig. 9d, e) and co-existence of *Pou3f1*-positive cells as well as *T*-positive cells in the proximal-posterior epiblast of E6.75 embryo (Supplementary Fig. 9d, f). Based on the fractions of re-annotated cell types of every Geo-seq position, a series of Heterogeneity Map was constructed for the gastrula embryo (Fig. 3d), and the presence of all cell types in the three germ layers during gastrulation was identified (Fig. 3d and Supplementary Fig. 8).

### Inferring the developmental trajectory

The information on cellular heterogeneity further revealed the progressive diversification of cell types in the germ layers (Fig. 3d), which might mirror the events of lineage development during gastrulation. The maps showed that a homogeneous cell population is found in the epiblast and ectoderm domains initially, and the heterogeneous cell types emerge during gastrulation in cell populations in the posterior epiblast/ectoderm, the PS, the mesoderm, and the endoderm. To reveal the developmental connectivity of the cell populations with increasing heterogeneity, the Population Tracing algorithm (Supplementary Fig. 2f and “Methods”) was applied to infer the putative molecular trajectory of the PS-like cells (PSLCs) in the posterior epiblast of E6.5 embryo to cells in the germ layers at advancing stages of gastrulation (Fig. 3d). Embedded in the transcriptome of the inferred trajectories is the information of the molecular regulatory networks that drive lineage development, for example, the derivation of PS-Mes cells from E6.5 PSLCs, and subsequent allocation of PS-Mes cells to diverse mesodermal cell types at E7.5 (Fig. 3e).

Cells that were originally annotated as “PS” were highly correlated with the PSLCs in our study. It is worth noting that the PSLCs diverge to progenitors of three germ layers at E6.75. A portion of cells in the proximal-posterior epiblast contributes to the epiblast/ectoderm lineage, while the bulk of PSLCs is partitioned into proximal and distal cell groups that were allocated to mesoderm and mesendoderm lineages. The “PS→Mes” cells from proximal PS contribute different mesoderm derivatives at later stages, including the blood and cardiac mesoderm that emerge from proximal PS-mesoderm populations at E7.0. Uniquely, the PSLCs in the E7.25 distal PS contain a cell population that may be the putative MEP, potentially contributing to both mesoderm and endoderm lineages (Fig. 3e and Supplementary Fig. 10a, b). Overall, the inferred molecular trajectories are broadly in line with the prospective fate of the epiblast cells, which provide an entry point for inferring the molecular control of the specification and differentiation of cell types derived from the PS-like cells at successive time points of germ layer development.

### Chirality of cell populations in proximal epiblast

**Fig. 4:**
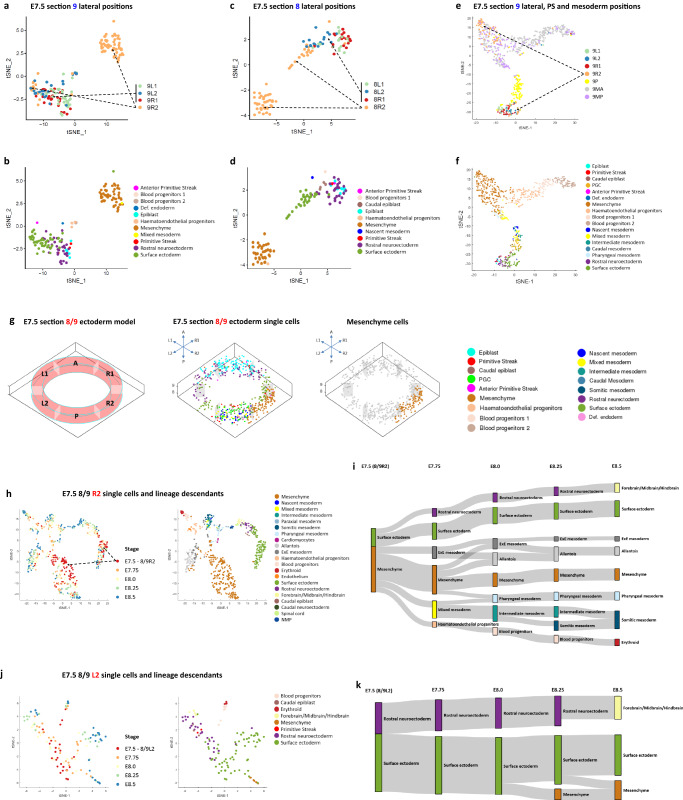
Heterogeneity of cell types and developmental trajectories of single cells in the proximal-lateral ectoderm of E7.5 embryo. **a**–**f** *t*-SNE plots showing the heterogeneous cell clusters in position-9R2 and -8R2. Cells are annotated by position (**a**, **c**, **e**) and cell type (**b**, **d**, **f**). Dashed arrows (in **a**, **c**, **e**) denote the heterogeneous cell clusters in position-9R2 and -8R2 versus other lateral positions. Panel **a**–**d** showed the single cells of proximal-lateral ectoderm positions in sections 9 (**a**, **b**) and 8 (**c**, **d**). Panel **e**, **f** showed the single cells of proximal-lateral ectoderm, primitive streak, and mesoderm positions in section 9. **g** The spatial distribution of single cells in the Annulus Model of ectoderm at Geo-seq section 8/9. **h**–**k** *t*-SNE plots (**h**, **j**) and the molecular trajectories (**i**, **k**) of single cells (imputed using the population tracing algorithm) at position-8R2/9R2 (**h**, **i**) and position-8L2/9L2 (**j**, **k**) in E7.5–E8.5 embryos. Developmental time points (stage) and cell types (see legend) are indicated in the *t*-SNE plots. Cell types in E7.75–E8.5 embryos are annotated according to the ‘Gastrulation Atlas’.

An unexpected discovery of the cellular heterogeneity of the epiblast was the finding of different cellular composition of the proximal-lateral epiblast located on contralateral sides (positions 8R2 and 9R2 versus 8L2 and 9L2) of the E7.5 embryo. The populations on the right side are an admixture of cell types, while those on the left side are homogeneous (Fig. 3d, e). To delineate the diversity of cell types in the population, we computationally re-clustered the single cells mapped to position-8R2 and -9R2 and the adjacent positions: 8R1, 9R1 and contralateral positions 8L1, 8L2, 9L1 and 9L2 in the proximal-lateral epiblast, as well as position-9P in the PS and position-9MA and -9MP in the mesoderm. The clustering analysis affirmed the heterogeneity of cell types in positions 8R2 and 9R2 (Fig. 4a–d and Supplementary Fig. 11a–d)—in particular, a large fraction of the cells at these two positions were allocated to a distinct cluster that was enriched for mesenchyme characteristics^12^, in contrast to the remaining cells, which grouped with cells from other positions and were enriched for ectoderm characteristics. The mesenchyme cell type is transcriptionally similar to cells in the mesoderm and is tightly clustered with cells in position-9MA and -9MP (Fig. 4e, f and Supplementary Fig. 11e, f). Applying the Euclidean distance-based Optimization Algorithm, we recapitulated the spatial distribution of single cells within ectoderm section-8/9 (Fig. 4g), where mesenchyme-like cells, distinct from other cell types, were enriched in position-8/9R2.

To identify the descendants of these cell clusters in position-8R2 and -9R2, we applied the Population Tracing algorithm to infer the descendants of these cells in E7.75–E8.5 embryos (based on single-cell data in the “Gastrulation Atlas”)^12^ (Supplementary Fig. 11g). The cell clusters follow different trajectories, with the mesenchyme cluster giving rise to a multitude of mesodermal tissues, and the ectoderm cluster to the surface ectoderm and rostral neuroectoderm (Fig. 4h, i and Supplementary Fig. 11h). These two cell clusters at position 8/9R2 are likely to be the progenitors of the lateral mesoderm, and the surface ectoderm and neuroectoderm respectively. In contrast, cells that populate the other six proximal-lateral positions (8/9L1, 8/9L2, and 8/9R1) contribute primarily to ectoderm derivatives and make a minor contribution to ectomesenchyme (presumptively the neural crest cells) (Fig. 4j, k and Supplementary Fig. 11i–l). Consistent with previous findings^11^, anterior lateral epiblast (L1, R1) contributes mainly to the neuroectoderm lineage, whereas posterior lateral epiblast (L2) preponderantly contributes descendant to the surface ectoderm. This preponderant contribution of the posterior proximal epiblast on the right side of the embryo to the mesoderm derivatives may implicate an alignment of this developmental event to the specification of left–right (L–R) body asymmetry of the embryo.

### Initiation of laterality in the body plan

**Fig. 5:**
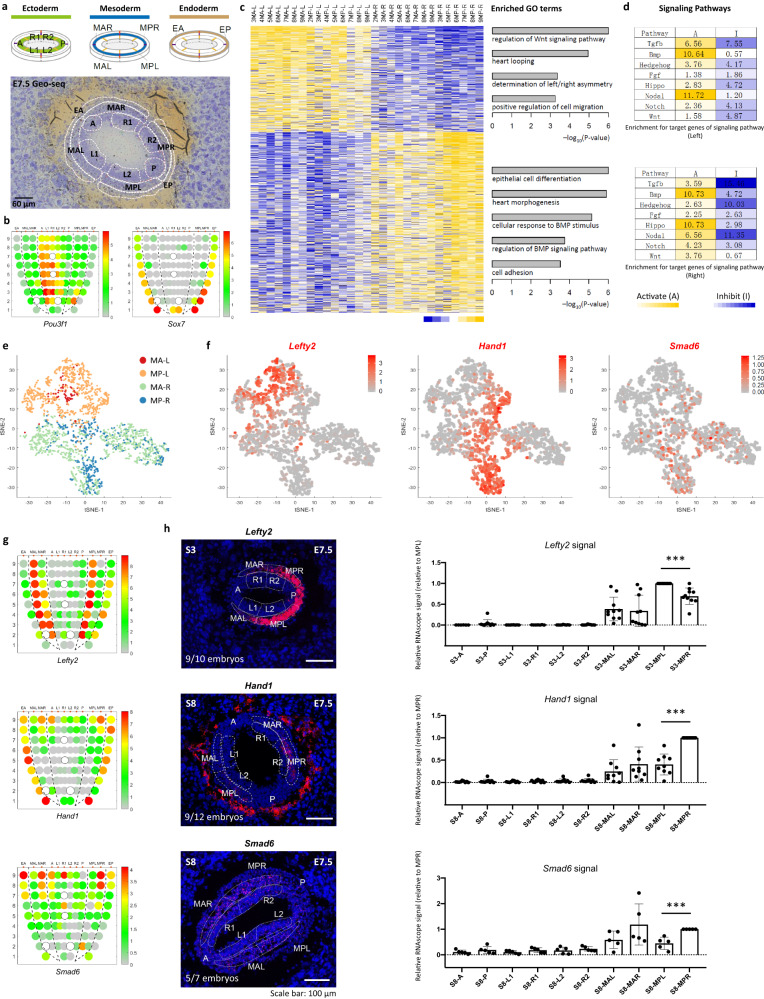
Left–right asymmetry at the late-gastrulation stage. **a** The strategy of laser capture microdissection of cell samples of E7.5 embryos. For the ectoderm and endoderm germ layers, the same Geo-seq strategy was applied as in Supplementary Fig. 1. The mesoderm germ layer was partitioned into MAL (anterior left mesoderm), MAR (anterior right mesoderm), MPL (posterior left mesoderm) and MPR (posterior right mesoderm) areas for sampling. Sampling areas are shown in histology images; scale bar, 60 μm. Three biologically independent sequencing replicates were prepared. **b** Corn plots showing the spatial pattern of expression of *Pou3f1* and *Sox7*. Hollow circles indicate missing samples. **c** Heat map showing the differentially expressed genes (DEGs) of the left lateral mesoderm (*n* = 518) and right lateral mesoderm (*n* = 881) (one-sided test, *p* < 0.05, fold change >1.5). The enriched gene ontology (GO) terms for each group were listed on the right (*p* < 0.01). **d** The enrichment for target and response genes of development-related signaling pathways in the left and right mesoderm. Signaling activity: red, activating (A); green, inhibitory (I). The significance of −log_10_(FDR) value in each cell was calculated by one-sided Fisher’s exact test followed by Benjamini–Hochberg correction. **e** Deconvolution analysis inferred the proportion of left and right lateral mesoderm cell populations and visualized on a t-SNE plot. Cells are colored by inferred positions: MA-L, MP-L, MA-R, and MP-R. **f** *t*-SNE plots showing the distribution of *Lefty2*-expressing cells in the left mesoderm and *Hand1-* and *Smad6-*expressing cells in the right mesoderm. **g** Corn plots showing the distribution of *Lefty2*-expressing cells in the left mesoderm and *Hand1-* and *Smad6-*expressing cells in the right mesoderm. **h** RNAscope analysis validated the bilaterally asymmetric expression of *Lefty2*, *Hand1*, and *Smad6* in the selected transverse sections (S-numbered, reference: (**g**)). The right panels summarize the quantified signal intensity and statistical results, data are presented as mean values ± S.E.M, *n* = 10 for *Lefty2*, *n* = 9 for *Hand1*, *n* = 5 for *Smad6*, n represents biologically independent samples subject to RNAscope analyses. Student’s *t*-test was performed, *** represents *p*-value < 0.001, the exact *p*-values for *Lefty2*, *Hand1*, *Smad6* group were 0.00011, 1e−6, 0.00086, respectively.

To examine the L–R asymmetry of the germ layers in more depth, we performed a Geo-seq analysis focusing on the domain of molecular activity in the mesoderm of E7.5 embryos (Fig. 5a, b and Supplementary Fig. 12a–f). In addition to the L–R difference in the proximal ectoderm (Supplementary Fig. 13a, c–e, h), we noticed that the proximal R2-related genes were more divergent in the right-side proximal lateral mesoderm (Supplementary Fig. 13b, f, g, i). Analyses of the spatial transcriptome revealed that the mesoderm cell population on contralateral sides displayed different profiles of gene expression and enrichment of functional gene ontology (Fig. 5c and Supplementary Fig. 14a). Analysis of whole-population transcriptome revealed left versus right differences in the enrichment of signaling activity of TGFβ, BMP, Hippo and Nodal pathway (Fig. 5d). By deconvolution analysis using the spatial transcriptome data, single mesoderm cells were allocated to the four domains (MA-L, MP-L, MA-R, MP-R) in the mesoderm layer (Fig. 5e, f and Supplementary Fig. 14b). Analysis of the transcriptome of the single cell populations revealed that the proximal mesoderm population (sections 7–9) on contralateral sides of the embryo displayed different gene expression profiles (Supplementary Fig. 14c and Supplementary Data 5 and 6) and the BMP and Hippo pathway activity was enriched in the mesoderm on the right side of the embryo (Supplementary Fig. 14d). Lateral mesoderm populations on contralateral sides were transcriptionally distinct. The side-specific enhanced pattern of marker genes (left: *Lefty2*, right: *Hand1* and *Smad6*) was validated by RNAscope and RT-qPCR analyses (Fig. 5g, h and Supplementary Fig. 14e, f).

**Fig. 6:**
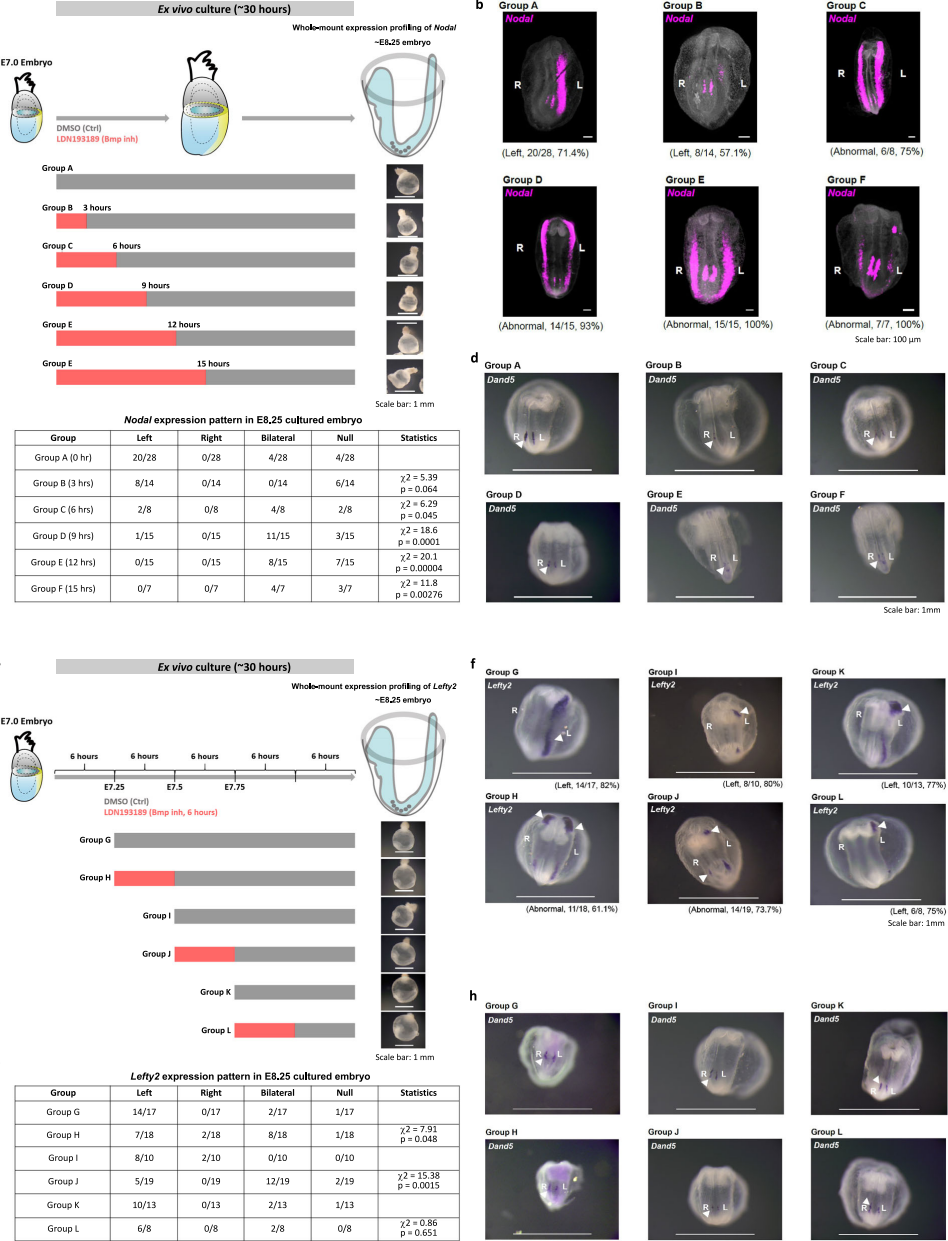
The temporal roles of gastrulation stage BMP signaling pathway in regulating left–right asymmetry. **a** Experimental strategy of ex vivo culture and analysis of embryos following chemical inhibition of BMP activity for 3, 6, 9, 12, and 15 h beginning on E7.0. **b** Whole-mount RNAscope analysis of embryos after 30 h of ex vivo culture, showing the expression of *Nodal* in the lateral mesoderm and the node (ventral view). The frequency of left-sided expression of *Nodal* is shown for Group A (untreated control) and Group B (3 h treatment), and the frequency of abnormal (bilateral) expression of *Nodal* is shown for Groups C (6 h treatment), D (9 h treatment), E (12 h treatment), and F (15 h treatment). **c** Table showing the pattern of *Nodal* expression in the cultured embryos collected at 30 h in vitro (equivalent to E8.25). A chi-squared test was performed to determine the statistical significance. **d** Whole-mount in situ hybridization of *Dand5*/*Cerl2* in cultured embryos from indicated experimental groups. Three biologically independent samples for each group were examined for consistency of gene expression pattern. **e** Experimental strategy of ex vivo culture and analysis of embryos following chemical inhibition of BMP activity for 6 h beginning at E7.25 (Group G and H), E7.5 (Group I and J), and E7.75 (Group K and L) stages. **f** Whole-mount in situ hybridization of *Lefty2* after ex vivo culture (equivalent to E8.25), showing the expression of *Lefty2* in the lateral mesoderm (ventral view). The frequency and embryo number of annotated patterns of *Lefty2* are shown for each group. **g** Table showing the pattern of *Lefty2* expression in the cultured embryos collected at equivalent E8.25 stages. A chi-squared test was performed to determine the statistical significance. **h** Whole-mount in situ hybridization of *Dand5*/*Cerl2* in cultured embryos from indicated experimental groups. Three biologically independent samples for each group were examined for consistency of gene expression pattern.

L–R asymmetry of the body plan is manifested at the early-organogenesis stages (E8.25–E8.5) by the looping of the heart and rotation of the epithelium of foregut portal^27,28^, and the asymmetric Nodal signaling activity and left-sided *Lefty2* expression in the lateral mesoderm (Supplementary Fig. 14g)^29,30^. In the mouse, the L–R tissue patterning is reputed to be initiated by asymmetric Nodal signaling activity in the node (the L–R organizer) at the post-gastrulation stage (~E7.75), coupling with the propagation of signaling activity to the lateral plate mesoderm (LPM) to activate *Nodal* and Nodal target genes by the early-somite stage (~E8.25, Supplementary Fig. 14g)^29,31^. BMP signaling has been known to modulate the Nodal signaling activity and the downstream signal transduction activity. During the establishment of laterality at the early-somite stage, symmetric BMP activity in the lateral plate mesoderm sets a bilateral repressive threshold for Nodal/Smad4-dependent *Nodal* activation. Super-activating the BMP activity by overexpressing constitutive ALK6 receptor in the mesoderm on the left side of the post-gastrulation mouse embryo can counteract the Nodal/Smad4 activity and lead to disruption of the asymmetric Nodal downstream gene activity in the lateral plate mesoderm^30^. To test the impact of asymmetric BMP signaling activity in the mesoderm at gastrulation on the establishment of body laterality, mid-streak embryos (E7.0) were treated ex vivo with BMP inhibitor (LDN193189) for a duration of 3 to 15 hours followed by Geo-seq analysis (at 12 hr ex vivo = E7.5 late-streak stage) or RNA in situ hybridization (at 30 h ex vivo = E8.25 early-somite stage) (Fig. 6a and Supplementary Fig. 15a). Early-somite stage embryos that were treated with inhibitor for more than 3 hours from E7.0 showed abnormal pattern of *Nodal* expression in the lateral mesoderm (Fig. 6a–c), with a reduced frequency in the left-sided expression, and an increased incidence of bilateral and absent expression (6 h: 75%, 9 h: 93%, 12 h: 100%, compared to control: 28%) (Fig. 6b, c). In the treated embryos (Group D: 9 hours treatment) at the stage equivalent to E7.5, Geo-seq analysis of the transcriptome revealed that BMP pathway activity was down-regulated in the right-side proximal lateral mesoderm while remaining unchanged on the L–R (Supplementary Fig. 15b) and the asymmetric pattern of gene expression in the lateral mesoderm has diminished (Supplementary Fig. 15c, d). The disruption of L–R asymmetry of Nodal activity in BMP-inhibited embryos raises the possibility that specification of the laterality of the body plan is initiated at the late-gastrulation stage, ahead of the formation and functionalization of the L–R organizer. In the BMP-inhibited embryos, *Nodal* and *Dand5* remained expressed in the peri-node tissue (Fig. 6b, d), suggesting that the action of BMP signaling in L–R patterning may be independent of the function of the L-R organizer. To delineate the stage-specific impact of BMP signaling on L–R patterning, the embryos were treated, beginning at different stages (E7.25, E7.5, and E7.75), for 6 h by LDN193189 inhibition (Fig. 6e). Blocking BMP activity at the mid-late streak stage (E7.25) and the late-streak stage (E7.5), but not at the early-head-plate stage (E7.75), led to disruption of the asymmetric *Lefty2* expression in the lateral plate mesoderm of the early-somite stage embryo (Fig. 6f, g), while *Dand5* expression at the node region was not consistently changed (Fig. 6h). These findings point to a stage-specific requirement of asymmetric BMP activity during late gastrulation for establishing L–R molecular asymmetry.

**Fig. 7:**
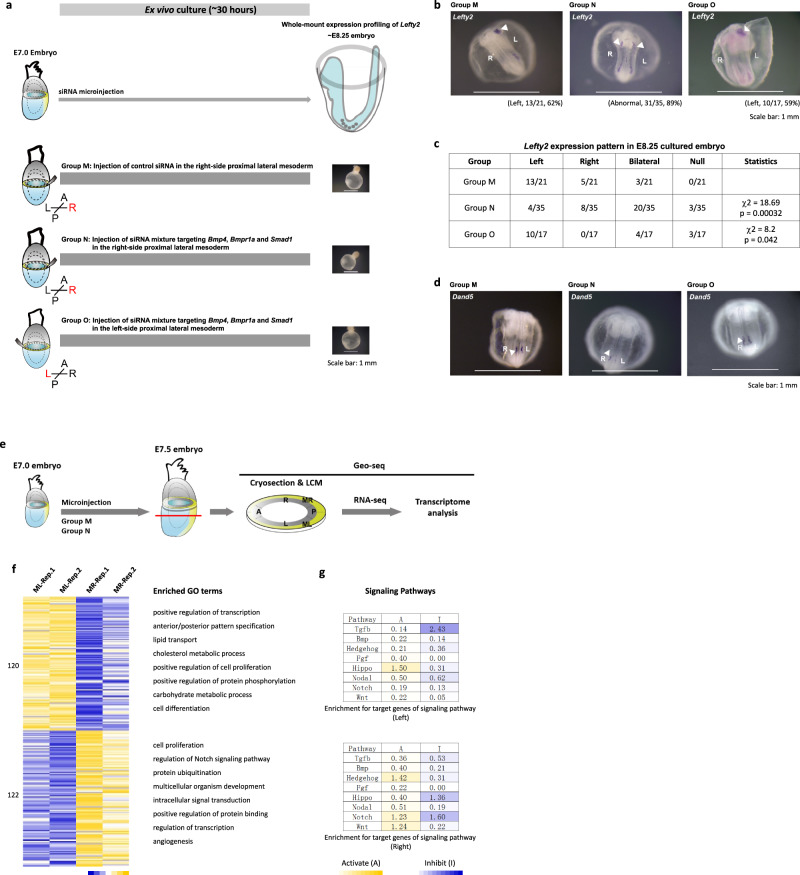
The role of the BMP signaling pathway in regulating left–right asymmetry during gastrulation. **a** Experimental strategy of ex vivo culture and analysis of embryos following siRNA microinjection beginning at E7.0 stage. Group M: Injection of control siRNA; Group N: Injection of siRNA mixture in the right-side mesoderm; Group O: Injection of siRNA mixture in the left-side mesoderm. **b** Whole-mount in situ hybridization of *Lefty2* after ex vivo culture (equivalent to E8.25), showing the expression of *Lefty2* in the lateral mesoderm (ventral view). The frequency and embryo number of annotated patterns of *Lefty2* are shown for each group. **c** Table showing the pattern of *Lefty2* expression in the cultured embryos collected at equivalent E8.25 stages. A chi-squared test was performed to determine the statistical significance. **d** Whole-mount in situ hybridization of *Dand5*/*Cerl2* in cultured embryos (equivalent to E8.25) from indicated experimental groups. Three biologically independent samples for each group were examined for consistency of gene expression pattern. **e** Schematic diagram showing the workflow of GEO-seq for microinjected embryos. **f** Heatmap showing the DEGs of the proximal-left mesoderm (*n* = 120) and proximal-right mesoderm (*n* = 122) (*p* < 0.01, fold change > 1.5) in the siRNA-KD embryos. Replicates: Rep-1, Rep-2. The enriched gene ontology (GO) terms for each group were listed on the right. **g** The enrichment for target/response genes of development-related signaling pathways in the proximal-left and proximal-right mesoderm of siRNA microinjected embryos. Signaling activity: red, activating (A); green, inhibitory (I). The significance of the −log_10_(FDR) value in each cell was calculated by one-sided Fisher’s exact test followed by Benjamini–Hochberg correction.

To elucidate whether the enhanced BMP activity on the right side of the gastrulating embryo is critical for the acquisition of L-R molecular asymmetry, E7.0 embryos were treated by combined siRNA knockdown (KD) of *Bmp4*, *Bmpr1a*, and *Smad1* in the right lateral mesoderm (Fig. 7a and Supplementary Fig. 15e). The triple siRNA-KD embryos cultured to the early-somite stage displayed increased incidence of bilateral, ectopic and no expression of *Lefty2* in the lateral plate mesoderm (Fig. 7b, c), but proper node formation (Fig. 7d). Geo-seq analysis revealed that the early asymmetry, which was maintained in control embryos, was abolished after siRNA KD (Fig. 7e and Supplementary Fig. 15f, g). Differentially expressed genes (DEGs) analysis of left versus right mesoderm of the siRNA-KD embryos identified fewer DEGs than in control embryos and revealed no significant contralateral difference in the enrichment of BMP signaling (Fig. 7f, g). However, embryos that were subject to siRNA KD in the left lateral mesoderm maintained asymmetric *Lefty2* expression and proper node formation (Fig. 7a–d and Supplementary Fig. 15e). These results point to a critical requirement of the enhanced BMP activity on the right side of the embryo at late gastrulation stage for the establishment of laterality of the body plan.

To further elucidate whether the BMP signaling in the gastrulating embryo has any mechanistic connectivity to the function of L-R organizer^32^ in mediating ciliary nodal flow and signal transduction, we assessed the impact of loss of *Pkd1l1*, *Cerl2*, and *Dnah11* function^33–36^ on the asymmetric BMP signaling activity in the embryo. At the early somite stage (E8.25–E8.5), all three loss-of-function mutants displayed disruption of the asymmetric pattern of *Lefty2* expression (Supplementary Fig. 16a, b, d, e, g–i), that phenocopied the effect of abolishing asymmetric BMP activity at late gastrulation. However, the asymmetric expression pattern of *Lefty2* in the left mesoderm and *Hand1* in the right mesoderm of the mutant embryos at the late gastrulation stage (E7.5) remained unchanged (Supplementary Fig. 16c, f, j and Supplementary Fig. 17a–c). Thus, enhancement of BMP signaling activity in the mesoderm on the right side of the body at late gastrulation may represent an early symmetry-breaking event that is independent of the formation and function of the L–R organizer.

## Discussion

This time and space-resolved single-cell molecular atlas of mouse gastrulation adds to the knowledge base of the developmental trajectory of cell populations in the germ layers at this milestone of embryo development. Analysis of the transcriptome of single cells mapped to specific Geo-seq positions has revealed the heterogeneity of cell types in the populations, leading to the inference of single cell-based tissue lineages in the gastrulating embryo, exemplified by the developmental trajectories of PS/PSLCs to multiple mesoderm and endoderm lineages. Re-annotation of the single cells leads to the identification of new cell types within each lineage, which may be progenitors and their immediate descendants. Specifically, the transcriptome of the refined lineage trajectories across the timepoint of gastrulation can be utilized to infer the gene networks, e.g., the regulome, and the molecular activity of signaling pathways and morphogenetic interaction underpinning lineage specification, cell-fate decision, and germ layer tissue differentiation.

The knowledge of the heterogeneity of cell types in time and space offers new learning of the putative MEPs, which are presumed to possess the dual potential of producing mesoderm and endoderm derivates. Among the mouse embryonic stem cells, some single-cell clones behave like MEPs by contributing descendants to both mesoderm and endoderm lineages^37^. While MEPs have been identified in zebrafish and Xenopus^38,39^, whether these dual potential progenitors are present in the mouse embryo is not known. Transcriptome and gene expression analysis of *Eomes*-positive epiblast cells of the early-gastrulation embryo, which contribute descendant to both mesoderm and endoderm, showed no example of *Eomes*-positive cells that co-expressed mesoderm (*Mesp1*) and endoderm (*Foxa2*) markers, suggesting MEP-like cells are not present in the epiblast at early gastrulation. However, cells co-expressing *Mesp1* and *Foxa2* were found, albeit rarely, in the mesoderm layer and the PS of embryos at mid-gastrulation (E7.0)^40^. These putative bipotential cells that are presumed to be different from the progenitors of either the mesoderm or endoderm were found close to the position where the MEPs are identified in the E7.25 embryo (Fig. 3d, e). By analyzing single-cell transcriptome, our study has therefore captured these transient MEP populations in time and space in the gastrulating mouse embryo and predicted their contribution to the distal and perinodal endoderm, as well as to the anterior mesendoderm derived from the node^18,41^.

Combining Geo-seq, scRNA-seq, and computational modeling, we uncovered that asymmetric BMP signaling activity in the mesoderm of embryo at late gastrulation may play a role in initiating L–R body patterning. BMP signaling activity is known to modulate Nodal signaling activity in the lateral mesoderm during the specification of L–R asymmetry^30^. The enhanced BMP activity at late gastrulation may constrain Nodal signaling activity on the right side of the embryo, thereby predisposing the contralateral side to perceiving and responding to Nodal activity emanating from the L–R organizer. Whether the BMP activity in the mesoderm may influence the mesoderm-lineage propensity of the proximal lateral epiblast on the right side of the gastrulating embryo, and if the enhanced contribution of the epiblast to the lateral mesoderm has a role in L–R patterning is not known at this juncture. Nevertheless, our findings have pointed to that the specification of L–R asymmetry may be initiated at late gastrulation, ahead of the acquisition of functionality of the L–R organizer. This study of L–R patterning highlights the attribute of the spatio-temporal molecular atlas to advance our understanding of early mammalian development, which is an empowering resource for guiding future research on the molecular and cellular mechanism of lineage differentiation and tissue patterning in the post-implantation mouse embryos.

## Methods

### Sampling of mouse embryos for Geo-seq analysis

All animal experiments were performed in compliance with the guidelines of the Animal Ethical Committee of the CAS Center for Excellence in Molecular Cell Science, Chinese Academy of Sciences. C57BL/6J embryos were harvested from pregnant mice at Day 6.5, 6.75, 7.0, 7.25, and 7.5 of gestation (day of vaginal plug detection = Day E0.5). The sex of the embryo specimens was not recorded in this study. The embryo’s progression of gastrulation at the five developmental time points was staged by the proximal-distal span of the PS and anterior-posterior span of the mesoderm layer^42^. The staging was further confirmed by the spatial domain of *T-* and *Mixl*-expressing cell populations in the posterior (P) samples of the embryo. Embryos were collected immediately after harvesting in OCT medium (Leica Microsystems, catalog No. 020108926). Cryosections of the embryo were processed for laser capture dissection to collect cell samples from the germ layers as previously described^11^. Cell samples were processed for Geo-seq analysis^16^. The acquired cDNA was allocated for RT-qPCR analyses of gene expression and library preparation for transcriptome profiling (Novozyme, TruePrep DNA Library Prep Kit V2 for Illumina, TD-503). Next-generation sequencing was performed on the Illumina Hiseq 2500 or Novaseq platform (Berry Genomics).

### Whole-mount in situ hybridization

RNA whole-mount in situ hybridization of the gastrula was performed as previously reported^43^. Briefly, embryos were fixed in 4% paraformaldehyde (PFA; sigma; #P6148) overnight, rehydrated through the series of 75%, 50%, and 25% methanol at room temperature, washed by 3 times in DPBS, treated with 10 mg/mL proteinase K (Life Technologies, # AM2548) in PBS for 8 min and post-fixed for 20 min in 4% paraformaldehyde. Riboprobes were synthesized by DIG RNA labeling kit and in vitro transcription kit (Roche Applied science, 11277073910; mMESSAGE mMACHINE T7 ULTRA KIT, AM1345; MEGAclear™ Transcription Clean-Up Kit, AM1902) and the probes were amplified using primers listed in Supplementary Data 4. Post-fixed embryos were incubated with approximately 1 μg/mL of digoxigenin-labeled RNA probe at 68 °C overnight, washed and stained with anti-digoxigenin antibody to visualize the expression pattern of the gene transcripts hybridized with the riboprobes.

### RNAscope

OCT-embedded E7.5 mouse embryos were cryo-sectioned at 20 μm thickness serially from the distal to proximal region and mounted on electrostatic glass slides. Analysis of gene expression was performed using RNAscope® Multiplex Fluorescent Reagent Kit v2 (Advanced Cell Diagnostics, 323100) following manufacturer’s instructions with minor modifications (proteinase plus treatment for 15 min instead of proteinase IV for 30 min) using probes supplied by Advanced Cell Diagnostics: mm-Pou3f1 (436421), mm-Mesp1-C3 (436281-C3), mm-T-C3 (423511-C3), mm-Lefty2-C2 (436291-C2), mm-Nodal-C3 (436321-C3), mm-Smad6-C4 (528041-C4), mm-Hand1 (429651). Images were acquired using the Leica TCS SP8 STED system. The fluorescence intensity of each region was calculated using Image J software following standard steps (8-bit format transformation > setting a threshold for background removal > signal measurement). In order to minimize signal variance between embryos and experimental batches, the integrated fluorescent intensity of each region was normalized by calculating the relative ratio to the corresponding region (e.g., relative ratio to MPL region for *Lefty2*, relative ratio to MPR for *Hand1* and *Smad6*). For each RNAscope probe, at least three biological replicates were examined, and statistical significance was assessed by a student’s *t*-test.

### Small interfering RNA (siRNA) perturbation of BMP signaling

siRNA oligos for *Bmp4*, *Bmpr1a*, and *Smad1* tagged with Cy3 orange-fluorescent dye and fluorescein amidite (FAM)-labeled negative control siRNA were synthesized by GenePharm. The siRNAs were first dissolved using RNase-free water to make 50 μM stock solutions following the manufacturer’s instructions. The efficiency of each oligo for knocking down the transcripts was pre-determined in mouse embryonic stem cells. The siRNA with the highest KD efficiency was selected for the experiments. Equal volumes of the oligos for *Bmp4*, *Bmpr1a*, and *Smad1* were mixed into one siRNA preparation. For lipofectamine-mediated transfection, 4 μL siRNA mixture, 1.5 μL LipoFectamine 2000 (Invitrogen, 11668-027), and 4.5 μL OptiMEM were pre-mixed and incubated at room temperature for 5 min. E7.0 mid-streak stage mouse embryos were collected from the pregnant mice and transferred to drops of PB1 medium placed on the 37 °C warm plate under the microscope. Approximate 0.05 μL siRNA-lipid mix was micro-injected into the extracellular space between the ectoderm and endoderm of the right side or the left side of the recipient embryo using a flat-tip microinjection pipette^44^. Into control embryos, the same volume of negative control siRNA was injected. The injected embryos were cultured ex vivo as described below.

### Ex vivo culture of mouse embryo

Mid-primitive-streak (E7.0) embryos were cultured in the medium of 50% CMRL (Gibco, 11530037) and 50% rat serum, supplemented with 1 × Glutamax (Gibco, 35050061), 1×NEAA (Hyclone, SH30238.01), and Glucose (4 mg/mL) under 5% CO_2_ incubator at 37 °C. 1 μM chemical inhibitor of BMP signaling (LDN193189, Selleck, S2618-2 mg) was added to the culture medium in the respective experimental groups. The cultured embryos were collected at various time points (Figs. 6 and 7 and Supplementary Fig. 15) for whole-mount RNA in situ hybridization or Geo-seq. The whole-mount RNAscope experiments were performed following the published protocol^45^. Images were captured using the LiTone XL system (Light Innovation Technology Limited). Embryos subjected to Geo-seq were fixed in paraformaldehyde for 30 min at 4 °C, dehydrated in an alcohol series, embedded in OCT compound, and cryo-sectioned serially. Cells were sampled by laser capture microdissection following the published protocol^16^. The acquired samples were treated with protease K (Invitrogen, AM2546) at 56 °C for 30 min, and the lysate was precipitated in ethanol solution and prepared for RNA-sequencing as previously described^16^.

### Pre-processing of RNA-seq data

The sequencing quality of raw sequencing data was evaluated by FASTQC. Tophat2 v2.0.4 program^46^ was used to map raw reads to mm10 version of the mouse genome with the following parameters: -g 1 -N 4 --read-edit-dist 4 --microexon-search -G annotation.GTF. The mapping ratio was calculated based on the number of mapped reads and total reads for each sample. We calculated fragment per kilobase per million (FPKM) as expression level using Cufflinks v2.0.2 with default parameters^47^. For each embryo, genes with FPKM > 1.0 in at least two samples across all samples were retained for further analysis. Finally, the expression levels were transformed to logarithmic space by using the log2 (FPKM + 1).

### Identification of spatial domain and zipcodes

For E6.5 and E6.75 embryos, zipcodes were identified as follows: (1) applied an adaptive clustering algorithm, Bayesian Information Criterion-Super K means (BIC-SKmeans) algorithm^48^, to identify an optimum number of clusters that can best capture the variance in the data, these optimum clusters were defined as spatial domains, and (2) identify inter-domain DEGs using RankProd^49^ with *P* value < 0.05 and fold change > 1.5. The top 50 DEGs of each spatial domain were denoted as zip codes for E6.5 and E6.75 embryos.

For E7.0, E7.25, and E7.5 embryos, zipcodes were identified as follows: (1) Use ComBat^50^ to remove potential batch effects based on the expression of all genes (log_2_-transformed) due to variation between samples of different sequencing conditions. (2) Use Python program v2.7.13 to calculate the variance of each expressed gene across all samples and select the top 6000 genes as highly variable genes, then perform principle component analysis (PCA) using FactoMineR^51^ package in R. The top 50 highest and 50 lowest PC loading genes from the most significant PCs (E7.0, PC1–4; E7.25, PC1–5; E7.5, PC1–5), which showed inter-domain specific expression patterns, were denoted as zip codes for E7.0, E7.25, and E7.5 embryos respectively. Heatmaps were generated using Cluster 3.0 and JavaTreeView^52^.

### Population tracing algorithm

To trace the developmental trajectory of cell populations in different spatial domains, we developed a digital tracing algorithm with the following computational procedures: (1) Use the union of genes in zipcodes of pair of stages of interest as the input gene set, (2) calculate the Euclidean distance of any two domains from two embryos of adjacent stages (for example, the distance between *D_A1* and *D_B2*, *D-A1* denotes domain 1 at time point A, *D-B2* denotes domain 2 at time point B) (formula-1),

1 $$d\left(D{{\_}}A1,\, D{{\_}}B2\right)=\sqrt{\mathop{\sum }\limits_{i=1}^{k}{({D{{\_}}A1}_{i}-{D{{\_}}B2}_{i})}^{2}}$$

(3) Calculate the mutual nearest neighbors (minimum matrix distance and variation less than 10%, the formula-2 below) for each domain from one developmental timepoint against the domains from the next developmental timepoint and connect the spatial domains. This procedure was repeated across the timepoints from the beginning to the end of the developmental series.

2 $$\left({d}_{\max }-{d}_{\min }\right)/{d}_{\min } < \, 10\%$$

and (4) Convert the distance of any two connected spatial domains to logarithmic space by using log_2_ transformation and visualize the genealogy of cell populations by Sankey plot using Google Charts^11^.

### 3D Modeling for embryo structure and visualization of Geo-seq data

To mimic the spatial structure and visualize the spatial pattern of gene expression in the embryo, a geometric concentric-oval model that mirrored the architecture of the cup-shaped gastrula-stage mouse embryo was developed. The RNA-seq data of cell samples were assigned to the positions defined by spatial coordinates on the ‘3D corn plot’ model to depict the spatial pattern of expression of the gene or gene group of interest, with the expression levels indicated by a color scale computed from the transcript counts in the RNA-seq dataset.

### Multi-dimension single-cell mapping (MDSC mapping)

Based on the 3D model and the zipcode signature, a mathematical algorithm was developed for imputing the location of single cells in the germ layers of the mouse embryo. The mathematical operation included: (1) Calculate Spearman’s rank correlation coefficient (SRCC): The SRCCs between the expression values of the zip codes of each single cell and all samples of the reference embryo were computed to generate, for example, 74 SRCC values for each single cell against 74 Geo-seq samples of E7.0 embryo, (2) Apply a spatial smoothing algorithm to determine the high-confidence location of each cell. This mapping method contrasts with the previous imputation method^15^, in which single cells are mapped to the position of the maximum SRCC value. While the higher SRCC may indicate a strong probability of matching to a position, there were cases where a single cell could match to several adjoining positions. Therefore, for mapping the single cells, the top 3 matching positions with maximum SRCCs were extracted, then applied the formula-3 below to calculate a 3D coordinate:

3 $$f\left(x,\, y,\, z\right)=\mathop{\sum }\limits_{i=1}^{3}{{{{{\rm{SRCC}}}}}}_{i}\times \sqrt{{\left(x-{x}_{i}\right)}^{2}+{\left(y-{y}_{i}\right)}^{2}+{\left(z-{z}_{i}\right)}^{2}}$$

The distance between this location and every Geo-seq sample position was then calculated, and the sample with minimum distance was determined as the best-mapped position of the cell.

### Verification of the mapping efficiency

The efficiency of the MDSC Mapping pipeline was evaluated by mapping single cells manually isolated from known positions in E6.5, E6.75, E7.0, E7.25, and E7.5 C57BL/6J embryos. First, embryos were dissected from the decidua in 10% FBS-DMEM medium, and cells were isolated from a specific position of the embryo by mouth pipetting guided by microscopy. Altogether, 19 single cells from E6.5, 32 single cells from E6.75, 37 single cells from E7.0, 24 single cells from E7.25, and 6 single cells from E7.5 were subjected to automatic Smart-seq2 amplification and library construction with the Agilent Bravo automatic liquid-handling platform^53^. Data preprocessing encompassed mapping, quality control, and normalization using the same criteria previously established^11^. Based on the transcriptome data of these single cells, their position was mapped to the reference embryo by MDSC Mapping.

To access the accuracy of the mapping methodology, we performed the following analysis: (1) Apply image processing to identify the spatial positions of single cells isolated by pipetting, (2) Apply Gaussian Distribution to mathematically simulate the Confidence Intervals. For E6.5–E7.25 embryos, we employ the standard normal distribution (formula-4) to simulate the confidence intervals:

4 $$f\left(x\right)=\frac{1}{\sqrt{2\pi }\sigma }{e}^{\left(-\frac{{\left(x-\mu \right)}^{2}}{{2\sigma }^{2}}\right)}$$

At E7.5, since the cell number is fewer, the standard normal distribution is adjusted to a more concentrated distribution: *f(x)* ≈ 1. (3) Calculate the Pearson Correlation Coefficients (PCC) between the Confidence Intervals and MDSC Mapping results. Through this modeling protocol, we showed that the single cells could be mapped at significant fidelity to their site of origin; the PCC of MDSC mapping per stage is 0.7399 (E6.5), 0.8893 (E6.75), 0.7943 (E7.0), 0.7602 (E7.25), and 0.9738 (E7.5).

### Single-cell datasets for positional mapping

The 10X Genomics single-cell data were downloaded as raw files from the Gastrulation Atlas: https://github.com/MarioniLab/EmbryoTimecourse2018. Steps of quality control, normalization, batch correction, and clustering were performed using the same criteria as previously described^12^.

### 3D modeling for single-cell resolution map

To visualize the spatial distribution pattern of single cells, an Annulus Model was developed to reconstruct the embryo spatial structure in single-cell resolution. The model comprises (1) Concentric annuli in the anatomical section of the embryo, from the inside outward, representing the epiblast/ectoderm, mesoderm, and endoderm germ layer, respectively. (2) Division of the annulus into interior spaces matching the Geo-seq defined position. Single cells that mapped to a Geo-seq position were distributed uniformly across the interior space of the position in each annulus section. (3) Within each interior space, apply the Bubble Sort Algorithm to align the cells along the known gradient of gene expression level or signaling intensity. For example, on the basis that *Bmp4* expression in the posterior epiblast streak decreases along the proximal-distal axis of the embryo, the algorithm code (see below) was applied to rearrange cells in domain 8 P in the E6.5 epiblast (x-cd, y-cd, and z-cd are abbreviations of 3D spatial coordinate (x, y, z)), based on the gradient of *Bmp4* expression:

for i in range len(8 P mapped SCs):

for j in range len(8 P mapped SCs)-i-1:

if z-cd[j] < z-cd[j + 1] and *Bmp4*[j] > *Bmp4*[j + 1]:

x-cd[j], x-cd[j+1] = x-cd[j+1], x-cd[j]

y-cd[j], y-cd[j+1] = y-cd[j+1], y-cd[j]

z-cd[j], z-cd[j+1] = z-cd[j+1], z-cd[j]

This imputation enabled the assignment of spatial coordinates specific for each single cell and (4) the construct of the 3D positional map for all single cells for the visualization of the spatial pattern of the single cells of different cell/tissue lineages that are individually defined by transcriptome features. Further information can be drawn from the 3D embryo map for the position of single cells displaying different levels of expression (indicated by the color scale computed from the transcript counts in the “Gastrulation Atlas”) of the gene/gene group of interest.

### The migrating mesoderm model

To model the mesoderm migration during gastrulation, we refined the Annulus Model by devising a Gradually Extended Annulus Model for the mesoderm of E7.0 and E7.25 embryos. From distal to proximal, the incomplete annuli that represent the anatomical section of the mesoderm progressively envelop the epiblast from posterior to anterior. At E7.25, the annuli of the proximal mesoderm (7–12 M) completely encircles the epiblast. The bubble Sort Algorithm was then applied to assign coordinates to each single cell within the interior domain space of each annulus of the mesoderm.

### Euclidean-distance derived optimal coordinates

To refine the spatial distribution pattern of single cells within each Geo-seq position, an optimization algorithm based on Euclidean distance was developed. The mathematical operations include: (1) Compute the gene-expression matrix (GEM) of single cells mapped to a specific position, and transform the matrix to logarithmic space using log_2_((normalized count) + 1). (2) Based on the logarithmic transformed matrix, calculate the Euclidean distance between every two cells to generate the Euclidean distance matrix (EDM). In order to adapt EDM to the interior space of the Annulus Model, the EDM was normalized through the formula-5 below to make the maximum matrix distance (*d*_max_) equivalent to the maximum length (length_max_) within the interior space.

5 $${{{{{\rm{EDM}}}}}}_{{{{{\rm{norm}}}}}}=\, {{{{{\rm{EDM}}}}}}\times \frac{{{{{\rm{{length}}}}}}_{\max }}{{d}_{{{{{\rm{max }}}}}}}$$

(4) Apply the least square method (formula-6) under the constraint conditions of the spatial mathematical model of the interior space to derive the optimized coordinate for each single cell:

6 $$f=\mathop{\sum }\limits_{i=1}^{n}\mathop{\sum }\limits_{j=1}^{n}{\left(\sqrt{{\left({x}_{i}-{x}_{j}\right)}^{2}-{\left({y}_{i}-{y}_{j}\right)}^{2}-{\left({z}_{i}-{z}_{j}\right)}^{2}}-{d}_{{ij}}\right)}^{2}\left\{\begin{array}{c}{x}^{2}+{y}^{2} \, < \, {r}^{2}\\ y \, > -x\\ 7.5 \, < \, z \, < \, 8.5\end{array}\right.$$

(This example shows the constraint conditions of the mathematical model for position 8 P at the E6.5 stage) and (5) visualize the spatial pattern of single cells of different types in each position. All the spatial algorithms and 3D modeling were operated using MATLAB.

### Heterogeneity analysis

To reveal the composition of the single-cell population mapped to a Geo-seq position, clustering analysis by *t*-SNE in Seurat^19^ was performed on the single cells for every position in the E6.5–-E7.5 embryos. The cell clusters were annotated on the basis of marker gene expression and the knowledge of the prospective fate of cells in specific regions of the embryo, gleaned from lineage tracing and fate mapping studies^25,26^. Based on the composition of annotated cell types in the single-cell population, a Heterogeneity Map was constructed for all Geo-seq positions in the gastrula-stage embryos, with the cell-type composition displayed as pie charts in the corn plots. To construct the molecular trajectory of specific cell types across the developmental stages, single cells of the same type or aligned with a specific lineage were grouped. The Population Tracing algorithm was applied using the averaged gene expression level data to infer the trajectories, which was visualized by the Sankey plot using Google Charts^11^.

### Nomenclature for cell type annotation

The nomenclature ‘X → Y’ and ‘X(Y)’ represent different cell states. X represents the germ layer information of the cell population. ‘X → Y’ indicates these cells are representing a transitional cell state from X to Y. And ‘X(Y)’ represents the precursor of a specified cell type (Y) in the germ layer X.

### Clustering and deconvolution of E7.5 mesoderm single cells

Transcriptome data of E7.5 mesoderm single cells were extracted from the ‘Gastrulation Atlas.’ The Seurat package^19^ was used to perform single-cell clustering analysis, and *t*-SNE was applied to visualize the results. To evaluate the representation of identified cell clusters in Geo-seq samples, we prepared cell-cluster labels for each single cell, the single-cell gene-expression matrix, and all Geo-seq samples of mesoderm from the left and right side of the E7.5 embryo. CIBERSORT^54^ was applied to perform cell-cluster deconvolution analysis with default parameters (without quartile normalization). The proportion of each cell cluster in the population was visualized on a *t*-SNE plot.

### Functional enrichment analysis

Functional enrichment of gene sets with different expression patterns was performed using the Database for Annotation, Visualization, and Integrated Discovery (DAVID)^55^ version 6.8.

### Signaling pathway enrichment analysis

In addition to BMP, FGF, Nodal, WNT, Notch, and Hippo-Yap signaling pathways^11^, Hedgehog, and TGFβ pathways were included in the analysis^56^. Potential signaling-target genes of each pathway were identified by comparing control samples with treatment samples using RankProd (*P* < 0.01 for Hippo-Yap and *P* < 0.001 for others) from published perturbation data (Gene Expression Omnibus accession numbers GSE48092, GSE41260, GSE17879, GSE69669, GSE15268, GSE31544, GSE58664, and GSE90567). Fisher’s exact test, followed by Benjamin–Hochberg correction, was applied to determine the significance of the overlap of the target genes of signaling pathways in different DEG groups.

### Generation of *Pkd1l1*, *Dand5* (*Cerl2*), and *Dnah11* (*iv*) mutant embryos

For the *Pkd1l1* rks mutant, substitution mutation in the *Pkd1l1* gene^33^ was generated by adenosine base editing. *Cerl2* mutation was generated by introducing a stop codon in the first exon, which phenocopied the Exon 2 deletion mutations^34^. For the *iv* mutant, the first P domain in the *Dnah11* gene was deleted^36^.

Small guided RNAs targeted to the respective genomic regions were designed by using the online tool Chop-chop (http://chopchop.cbu.uib.no/). The DNA fragments containing the T7 promoter and sgRNA sequence and scaffold were transcribed in vitro using the MEGAshortscript Kit (Invitrogen, AM1354), and the products were purified using the MEGAclear kit (Invitrogen, AM1908). DNA fragments for T7-CRISPR-Cas9^57^, T7-ABE 7.10^58^, and T7-YE1-BE4max^59^ were transcribed in vitro using MMESSAGE MMACHINE T7 Ultra Kit (Invitrogen, AM1345) and MEGAclear kit (Invitrogen, AM1908).

C57BL/6 female mice (4 weeks old) were superovulated and mated with the male C57BL/6 mice. Twenty-four hours later, fertilized embryos were collected from oviducts. T7-CRISPR-Cas9 (for *Dnah11* mutant), T7-ABE 7.10 (for *Pkd1l1* rks mutant), T7-YE1-BE4max (for *Cerl2* mutant) mRNAs (100 ng/µl), and corresponding sgRNA (100 ng/µl) were mixed in HEPES-CZB medium containing 5 μg/ml cytochalasin B (CB) and injected into the cytoplasm of fertilized eggs using a FemtoJet microinjector (Eppendorf) with constant flow settings. The injected embryos were cultured in KSOM with amino acids at 37 °C under 5% CO_2_ in the air to reach the 2-cell stage after 24 h in vitro. Two-cell embryos were transferred into pseudo-pregnant ICR female mice, and embryos were collected at E7.5 and E8.5 for analyses.

### Reporting summary

Further information on research design is available in the Nature Portfolio Reporting Summary linked to this article.

### Supplementary information

Supplementary Information

Peer Review File

Description of Additional Supplementary Files

Supplementary Data 1

Supplementary Data 2

Supplementary Data 3

Supplementary Data 4

Supplementary Data 5

Supplementary Data 6

Reporting Summary

### Source data

Source Data

## Data Availability

The RNA-seq data generated in this study were deposited in the NCBI Gene Expression Omnibus under accession number GSE171588. For the 10X Genomics data, raw and processed single-cell data can be downloaded following the instructions at https://github.com/MarioniLab/EmbryoTimecourse2018. Source data are provided in this paper. All other data are available from the corresponding authors upon request. Source data are provided in this paper.

## Code availability

We established a web portal—‘scGastrulation’ at http://119.78.66.195/ to make the space-, time-, and single-cell resolved transcriptome data available as a research resource for the scientific community.
