# Supplementary material for: Time space and single-cell resolved tissue lineage trajectories and laterality of body plan at gastrulation
Source: Nat Commun. 2023 Sep 14;14:5675. doi: 10.1038/s41467-023-41482-5 (PMC10502153; doi:10.1038/s41467-023-41482-5)
Supplement: Supplementary file 1 — Supplementary Information [file 41467_2023_41482_MOESM1_ESM.pdf]

Supplementary Information

Time Space and Single-Cell Resolved Tissue Lineage Trajectories and Laterality of Body Plan at Gastrulation

Ran Wang[1,11], Xianfa Yang[2,11], Jiehui Chen[1,11], Lin Zhang[1,11], Jonathan A. Griffiths[3,4,10], Guizhong Cui[2], Yingying Chen[2], Yun Qian[1], Guangdun Peng[5,9], Jinsong Li[1], Liantang Wang[6], John C. Marioni[3,4,12], Patrick P.L. Tam[7,8,12,*], Naihe Jing[1,2,5,9,12,*]

[1] *State Key Laboratory of Cell Biology, CAS Center for Excellence in Molecular Cell Science, Shanghai Institute of Biochemistry and Cell Biology, Chinese Academy of Sciences; 320 Yue Yang Road, Shanghai 200031, China;*

[2] *Guangzhou National Laboratory, No. 9 XingDaoHuanBei Road, Guangzhou International BioIsland, Guangzhou 510005, Guangdong Province, China;*

[3] *Cancer Research UK Cambridge Institute, University of Cambridge, Cambridge CB2 0RE, UK;*

[4] *European Molecular Biology Laboratory, European Bioinformatics Institute (EMBL-EBI), Cambridge CB10 1SD, UK;*

[5] *CAS Key Laboratory of Regenerative Biology, Guangdong Provincial Key Laboratory of Stem Cell and Regenerative Medicine, Guangzhou Institutes of Biomedicine and Health, Chinese Academy of Sciences, Guangzhou 510530, China;*

[6] *School of Mathematics, Northwest University, Xi'an 710127, China;*

[7] *Embryology Research Unit, Children's Medical Research Institute, University of Sydney, New South Wales, Australia;*

[8] *School of Medical Sciences, Faculty of Medicine and Health, University of Sydney, New South Wales, Australia;*

[9] *Institute for Stem Cell and Regeneration, Chinese Academy of Sciences, Beijing 100101, China;*

[10] *Current address: Genomics Plc, 50-60 Station Road, Cambridge, CB1 2JH, UK;*

[11] *These authors contributed equally;*

[12] *These authors jointly supervised this work: John C. Marioni, Patrick P.L. Tam, Naihe Jing;*

* Correspondence: ptam@cmri.org.au (P.P.L.T.), njing@sibcb.ac.cn (N.J.)

a

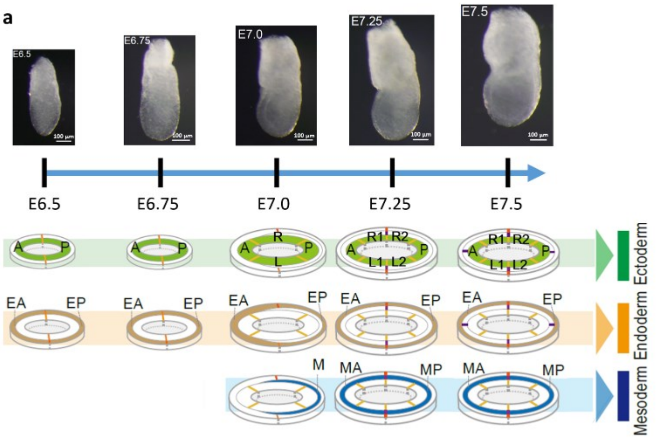

b

Stages	Replicates	Germ layer cells
E6.5	2	Epiblast, Endoderm
E6.75	2	Epiblast, Endoderm
E7.0	3	Epiblast, Mesoderm, Endoderm
E7.25	2	Epiblast, Mesoderm, Endoderm
E7.5	3	Ectoderm, Mesoderm, Endoderm

c

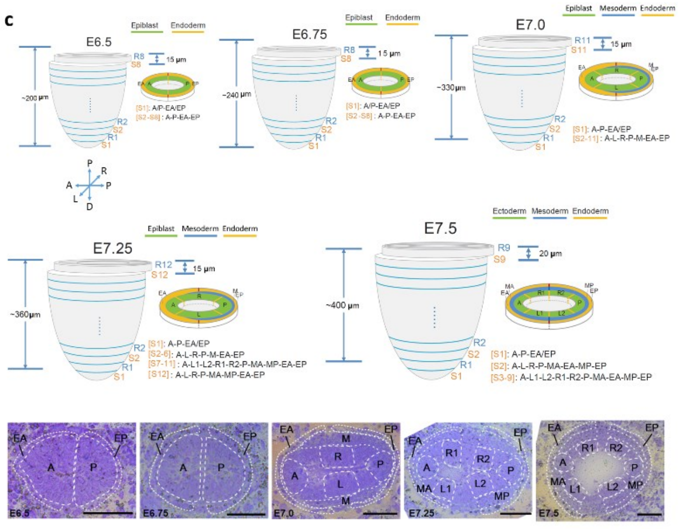

d

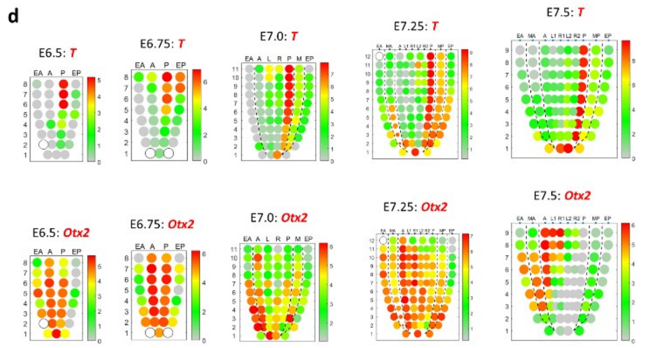

e f g

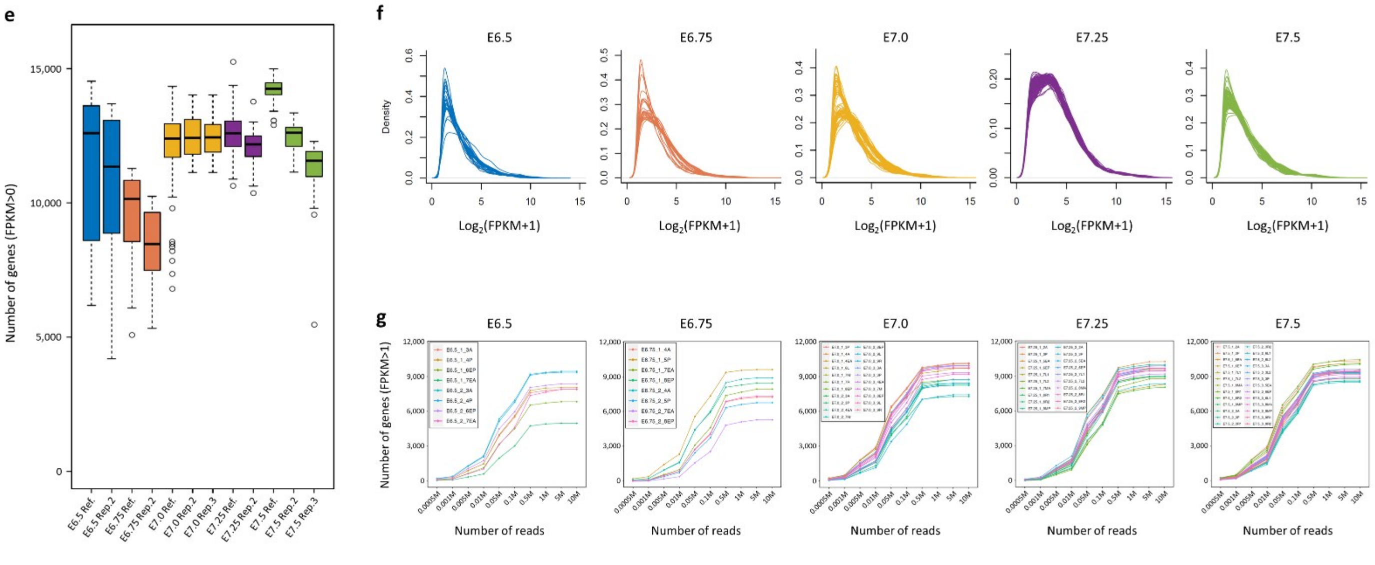

Supplementary Figure 1. Geo-seq analysis.

a. Schematics of laser capture microdissection of cell samples in E6.5-E7.5 embryos. A, anterior; P, posterior; L, left lateral; R, right lateral; L1, anterior left lateral; R1, anterior right lateral; L2, posterior left lateral; R2, posterior right lateral; M, mesoderm; MA, anterior mesoderm; MP, posterior mesoderm; EA, anterior endoderm; EP, posterior endoderm.

b. Samples for Geo-seq: embryonic stages, biological replicates and the germ layer cells. Stages: E6.5-E6.75, early-streak stage; E7.0-E7.25, mid- to late-streak stage; E7.5, late-streak to no-bud stage.

c. The strategy of sampling of cell populations in the epiblast/ectoderm and endoderm in E6.5 to E7.5 embryos and mesoderm in E7.0 to E7.5 embryos (areas of sampling shown in histology images). Samples were designated in ascending order of serial sections (1 = the most distal section) and the regions in the section (R, reference section; S, sample section). Embryonic axes: anterior – posterior, A ↔ P; proximal – distal, P ↔ D; left – right, L ↔ R. Scale bar, 50 μm.

d. Corn plots showing the spatio-temporal pattern of expression of *T* and *Otx2*. *T* expression domain marks the length of the primitive streak for staging the development of the embryo; *Otx2* is a representative ectoderm marker.

e. Box plot showing the number of detected genes (FPKM > 0) in samples of E6.5-E7.5 embryos (1 biological replicate for E6.5, E6.75 and E7.25 embryos; 2 biological replicates for E7.0 and E7.5 embryos). The center line marks the median and box edges represent 25th and 75th percentiles. The median of genes detected in reference embryo per stage is 11,033 (E6.5), 10,150 (E6.75), 12,340 (E7.0), 12,549 (E7.25) and 14,246 (E7.5).

f. Gene expression density plot of Geo-seq data of samples of E6.5-E7.5 embryos. The X-axis of the density plots is harmonized to the same scale.

g. Saturation analysis for reads for samples from each development stage. Different numbers of reads were selected, and the number of detected genes was plotted.

Panel 1

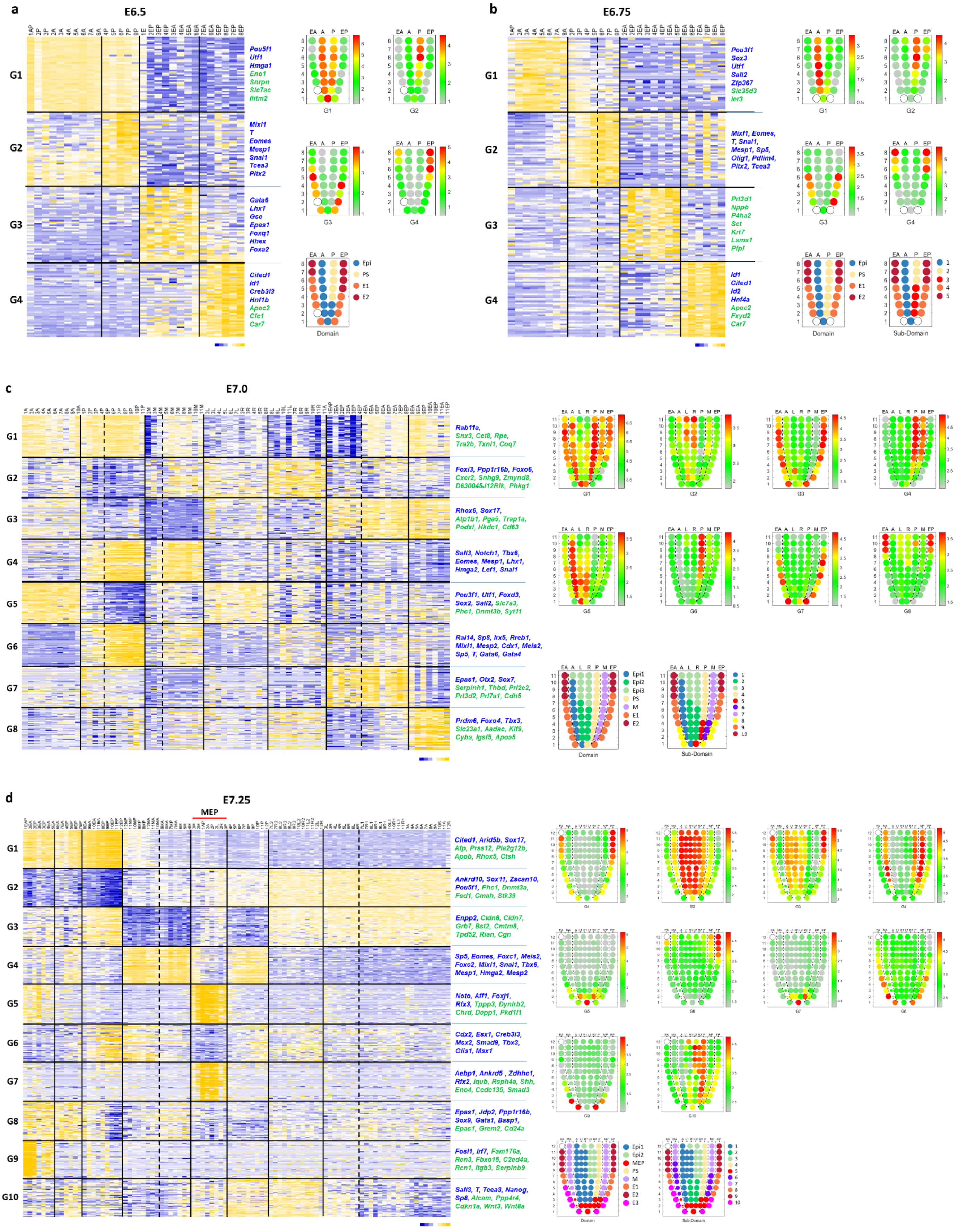

a
E6.5
G1
G2
G3
G4
Pou5f1
Utf1
Hmga1
Eno1
Snrpn
Slc7ac
Ifitm2
Mixl1
T
Eomes
Mesp1
Snai1
Tcea3
Pitx2
Gata6
Lhx1
Gsc
Epas1
Foxq1
Hhex
Foxa2
Cited1
Id1
Creb3l3
Hnf1b
Apoc2
Cfc1
Car7
EA A P EP
Epi
PS
E1
E2
Domain
b
E6.75
Pou3f1
Sox3
Utf1
Sall2
Zfp367
Slc35d3
Ier3
Mixl1, Eomes,
T, Snai1,
Mesp1, Sp5,
Olig1, Pdlim4,
Pitx2, Tcea3
Prl3d1
Nppb
P4ha2
Sct
Krt7
Lama1
Ptpl
Id1
Cited1
Id2
Hnf4a
Apoc2
Fxyd2
Car7
Sub-Domain
c
E7.0
G5
G6
G7
G8
Rab11a,
Snx3, Cct8, Rpe,
Tra2b, Txnl1, Coq7
Foxi3, Ppp1r16b, Foxo6,
Cxcr2, Snhg9, Zmynd8,
D630045J12Rik, Phkg1
Rhox6, Sox17,
Atp1b1, Pga5, Trap1a,
Podxl, Hkdc1, Cd63
Sall3, Notch1, Tbx6,
Eomes, Mesp1, Lhx1,
Hmga2, Lef1, Snai1
Pou3f1, Utf1, Foxd3,
Sox2, Sall2, Slc7a3,
Phc1, Dnmt3b, Sytl1
Rai14, Sp8, Irx5, Rreb1,
Mixl1, Mesp2, Cdx1, Meis2,
Sp5, T, Gata6, Gata4
Epas1, Otx2, Sox7,
Serpinh1, Thbd, Prl2c2,
Prl3d2, Prl7a1, Cdh5
Prdm6, Foxo4, Tbx3,
Slc23a1, Aadac, Klf9,
Cyba, Igsf5, Apoa5
EA A L R P M EP
Epi1
Epi2
Epi3
PS
M
E1
E2
d
E7.25
MEP
G9
G10
Cited1, Arid5b, Sox17,
Afp, Prss12, Pla2g12b,
Apob, Rhox5, Ctsh
Ankrd10, Sox11, Zscan10,
Pou5f1, Phc1, Dnmt3a,
Fsd1, Cmah, Stk39
Enpp2, Cldn6, Cldn7,
Grb7, Bst2, Cmtm8,
Tpd52, Rian, Cgn
Sp5, Eomes, Foxc1, Meis2,
Foxc2, Mixl1, Snai1, Tbx6,
Mesp1, Hmga2, Mesp2
Noto, Aff1, Foxj1,
Rfx3, Tppp3, Dynlrb2,
Chrd, Dcpp1, Pkd1l1
Cdx2, Esx1, Creb3l3,
Msx2, Smad9, Tbx3,
Glis1, Msx1
Aebp1, Ankrd5, Zdhhc1,
Rfx2, Iqub, Rsph4a, Shh,
Eno4, Ccdc135, Smad3
Epas1, Jdp2, Ppp1r16b,
Sox9, Gata1, Basp1,
Epas1, Grem2, Cd24a
Fosl1, Irf7, Fam176a,
Rcn3, Fbxo15, C2cd4a,
Rcn1, Itgb3, Serpinb9
Sall3, T, Tcea3, Nanog,
Sp8, Alcam, Ppp4r4,
Cdkn1a, Wnt3, Wnt8a
Epi1
Epi2
MEP
PS
M
E1
E2
E3
Domain
Sub-Domain

Panel 2

e

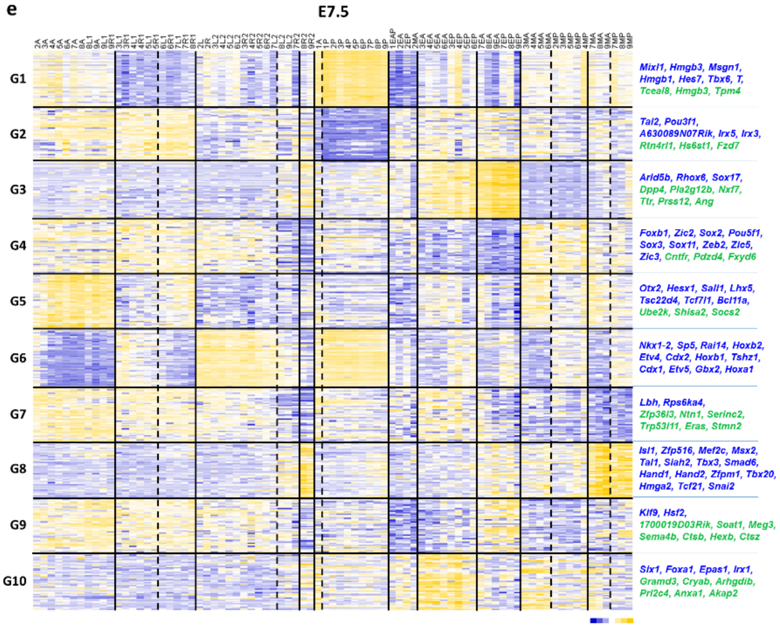

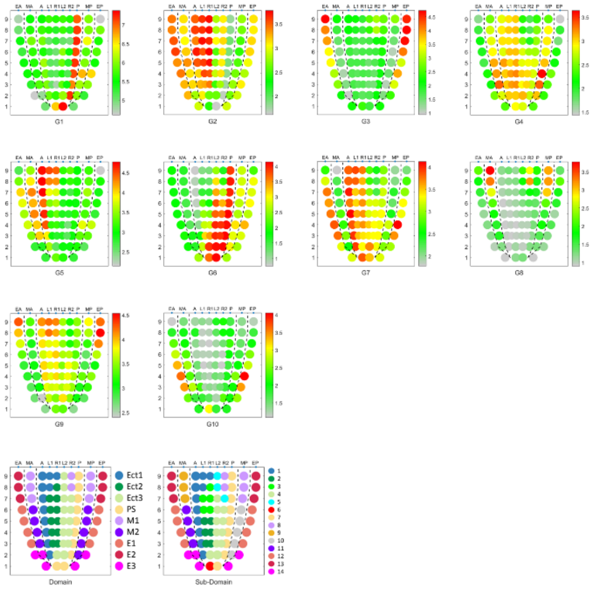

f

Time Point A → **Time Point B** → **Time Point C** → …… → **Time Point N**

$$d(\text{D\_A1},\text{D\_B2})=\sqrt{\sum_{i=1}^{k}(\text{D\_A1}_i-\text{D\_B2}_i)^2}$$

Dist.	Domain_B_1	Domain_B_2	...	Domain_B_n
Domain_A_1	d_{1-1}	d_{1-2}	...	d_{1-n}
Domain_A_2	d_{2-1}	d_{2-2}	...	d_{2-n}
...	$d_{...-1}$	$d_{...-2}$	...	$d_{...-n}$
Domain_A_m	d_{m-1}	d_{m-2}	...	d_{m-n}

Consecutive operations to identify the downstream developmental domains.

g

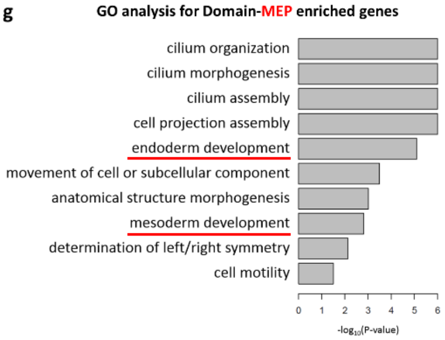

h

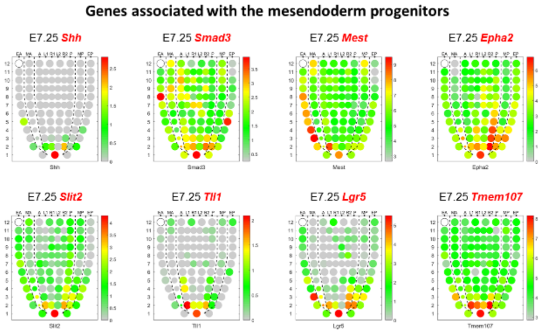

i

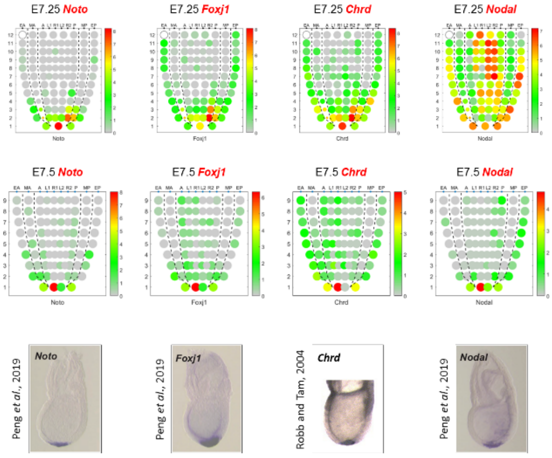

Supplementary Figure 2. The spatial domains in the germ layers of E6.5-E7.5 embryos delimited by the expression profile of zipcode genes.

a-e. Heat maps and corn plots of zipcode groups in E6.5 (**a**), E6.75 (**b**), E7.0 (**c**), E7.25 (**d**) and E7.5 (**e**) embryos. Transcription factors (blue marked) and top DEGs (green marked) of each gene group were listed on the right side of heat map. G, group.

f. Population Tracing algorithm. The Euclidean distance of any two domains in embryos of successive stages were computed, following by delineating the mutual nearest neighbors of each domain from one stage against the domains of embryo at the successive stage. D_A1 denotes domain 1 at time point A, D_B2 denotes domain 2 at time point B.

g. Functional ontology of enriched genes associated with the mesendoderm progenitors (MEP).

h. Corn plots showing representative genes associated with the MEP.

i. Expression pattern of MEP- and node-related genes in E7.25 and E7.5 embryo. The spatial transcriptome (corn plot) data of *Noto*, *Foxj1*, *Chrd* and *Nodal* were verified with reference to whole mount in situ hybridization (WISH) data. WISH images of *Noto*, *Foxj1* and *Nodal* from Peng *et al*. (2019), and *Chrd* from Robb and Tam (2004).

Panel 1

a

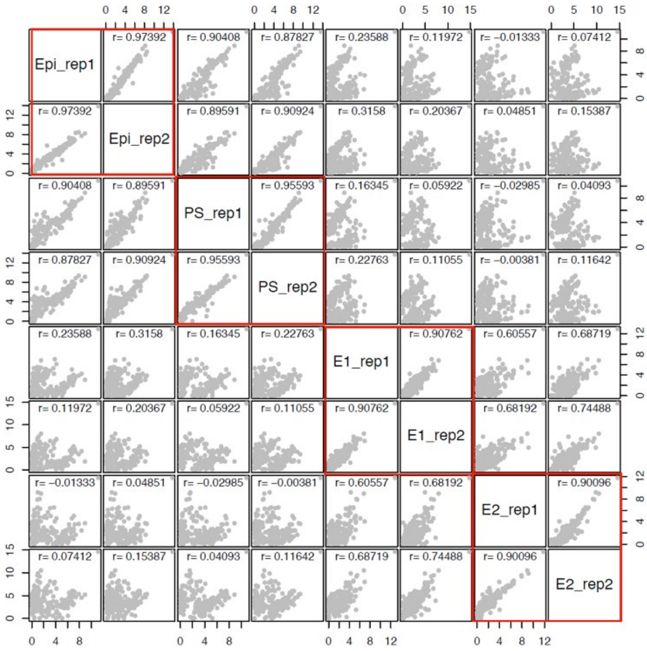
E6.5
Epi_rep1
Epi_rep2
PS_rep1
PS_rep2
E1_rep1
E1_rep2
E2_rep1
E2_rep2

b

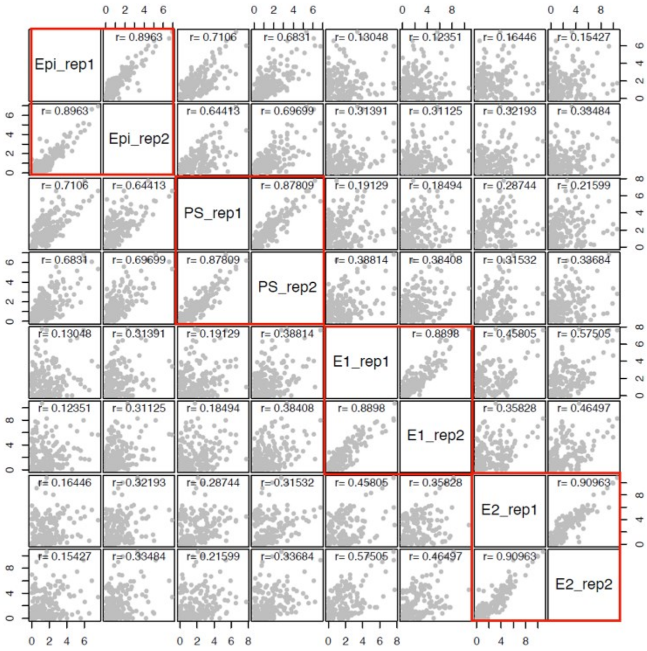
E6.75
Epi_rep1
Epi_rep2
PS_rep1
PS_rep2
E1_rep1
E1_rep2
E2_rep1
E2_rep2

c

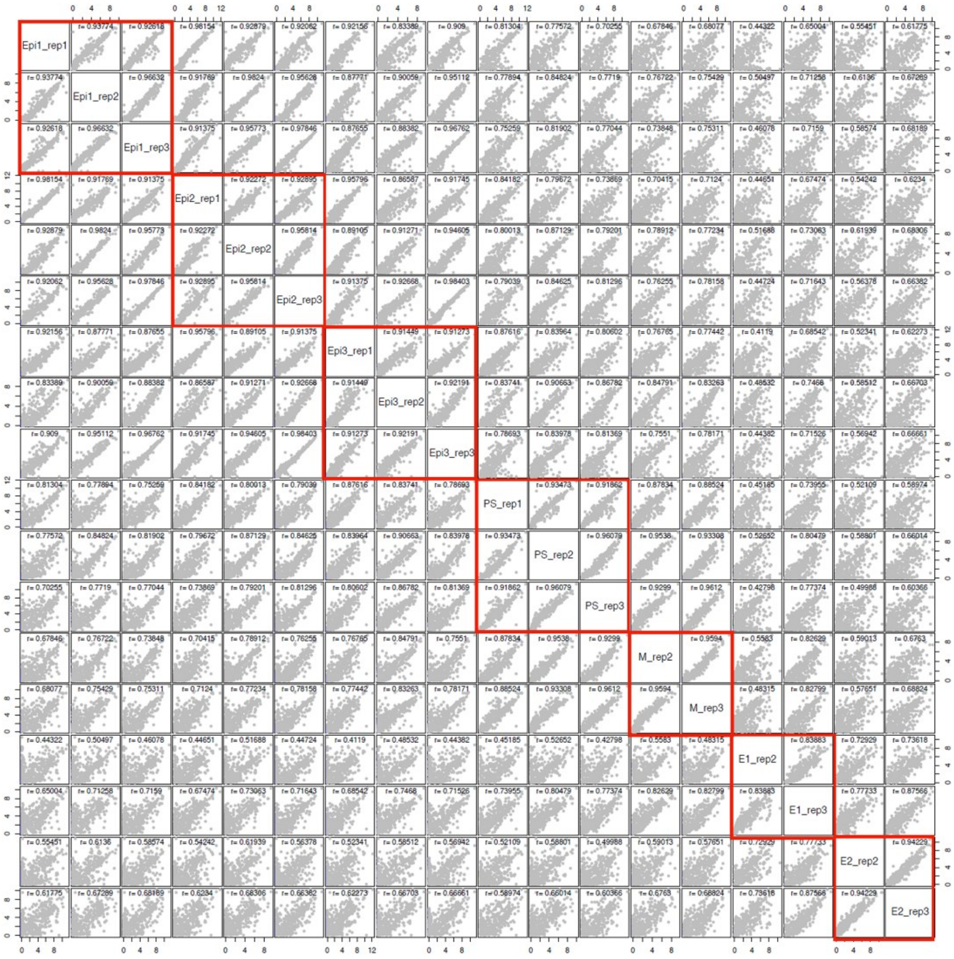
E7.0
Epi1_rep1
Epi1_rep2
Epi1_rep3
Epi2_rep1
Epi2_rep2
Epi2_rep3
Epi3_rep1
Epi3_rep2
Epi3_rep3
PS_rep1
PS_rep2
PS_rep3
M_rep2
M_rep3
E1_rep2
E1_rep3
E2_rep2
E2_rep3

Panel 2

d

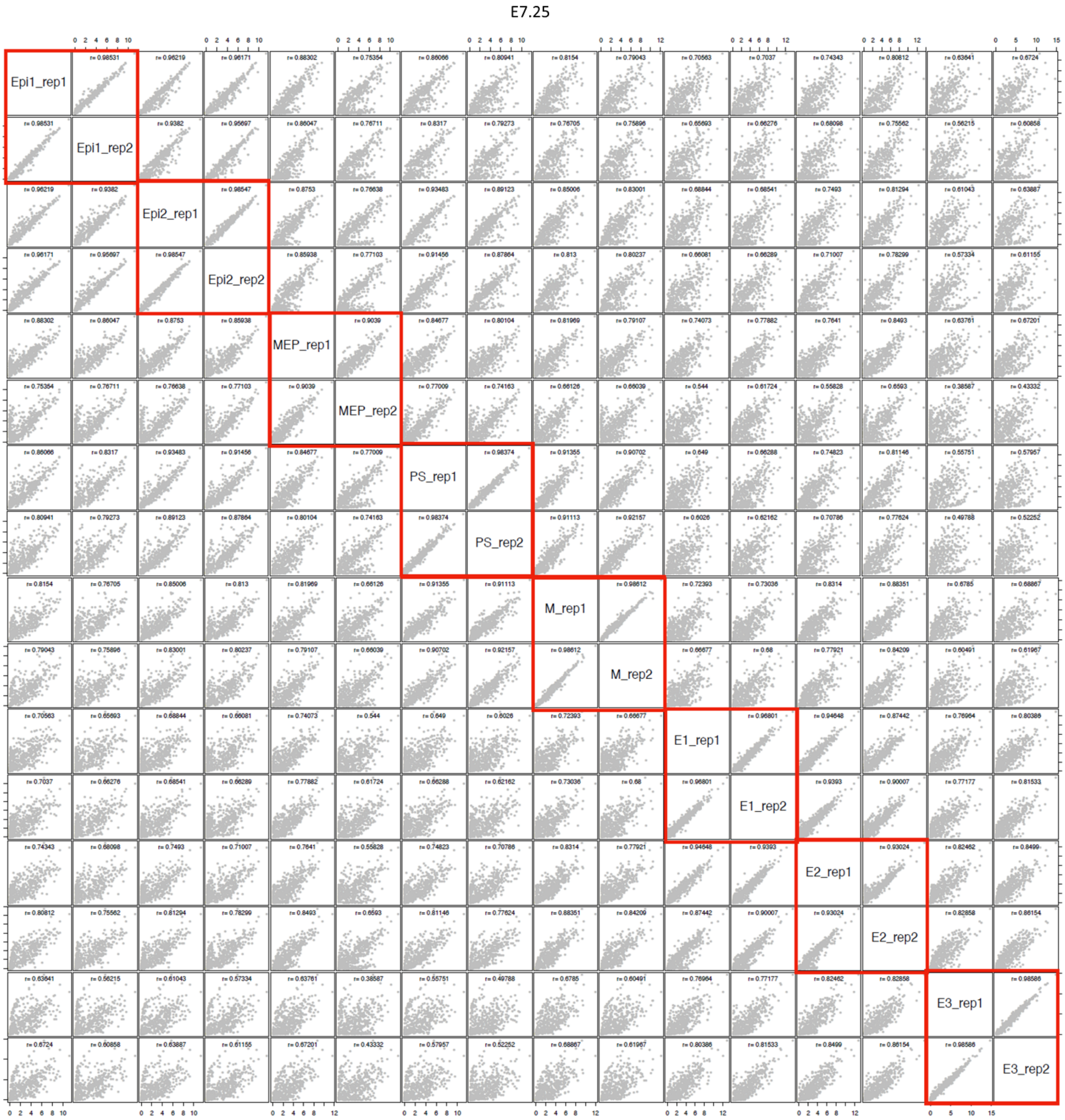
E7.25
Epi1_rep1
Epi1_rep2
Epi2_rep1
Epi2_rep2
MEP_rep1
MEP_rep2
PS_rep1
PS_rep2
M_rep1
M_rep2
E1_rep1
E1_rep2
E2_rep1
E2_rep2
E3_rep1
E3_rep2

Panel 3

e

E7.5

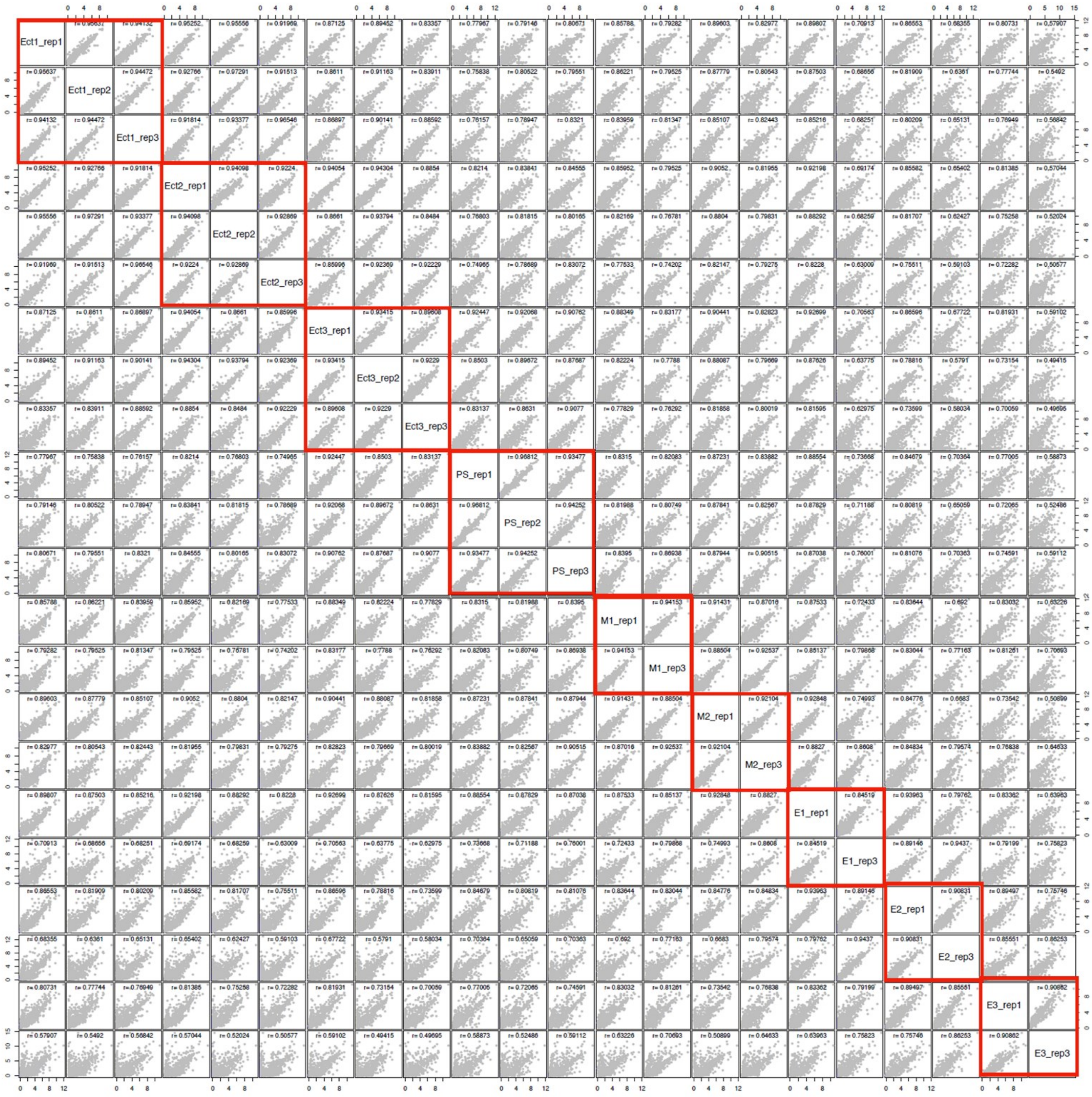
Ect1_rep1
Ect1_rep2
Ect1_rep3
Ect2_rep1
Ect2_rep2
Ect2_rep3
Ect3_rep1
Ect3_rep2
Ect3_rep3
PS_rep1
PS_rep2
PS_rep3
M1_rep1
M1_rep3
M2_rep1
M2_rep3
E1_rep1
E1_rep3
E2_rep1
E2_rep3
E3_rep1
E3_rep3

Supplementary Figure 3. Inter-embryo correlation of spatial transcriptome data.

a-e. The Pearson Correlation Coefficient (PCC) of spatial domains between biological replicates at E6.5 (**a**), E6.75 (**b**), E7.0 (**c**), E7.25 (**d**) and E7.5 (**e**). For E7.0 replicate 2 and E7.5 replicate 2, only epiblast/ectoderm domains were assessed.

Panel 1

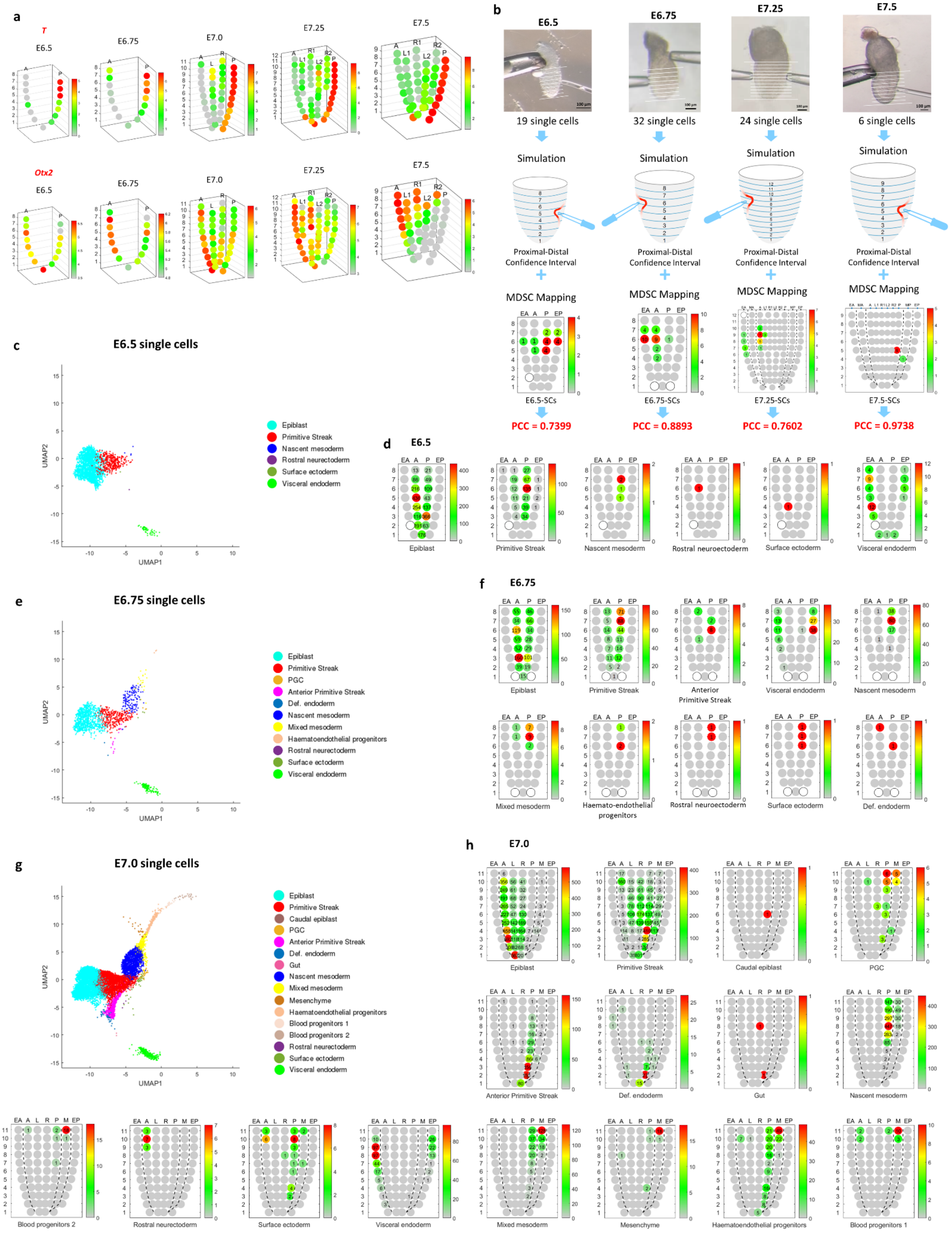

a
T
Otx2
E6.5
E6.75
E7.0
E7.25
E7.5
b
E6.5
E6.75
E7.25
E7.5
19 single cells
32 single cells
24 single cells
6 single cells
Simulation
Proximal-Distal Confidence Interval
MDSC Mapping
E6.5-SCs
E6.75-SCs
E7.25-SCs
E7.5-SCs
PCC = 0.7399
PCC = 0.8893
PCC = 0.7602
PCC = 0.9738
c
E6.5 single cells
UMAP1
UMAP2
Epiblast
Primitive Streak
Nascent mesoderm
Rostral neurectoderm
Surface ectoderm
Visceral endoderm
d
E6.5
Epiblast
Primitive Streak
Nascent mesoderm
Rostral neuroectoderm
Surface ectoderm
Visceral endoderm
e
E6.75 single cells
Epiblast
Primitive Streak
PGC
Anterior Primitive Streak
Def. endoderm
Nascent mesoderm
Mixed mesoderm
Haematoendothelial progenitors
Rostral neurectoderm
Surface ectoderm
Visceral endoderm
f
E6.75
Epiblast
Primitive Streak
Anterior Primitive Streak
Visceral endoderm
Nascent mesoderm
Mixed mesoderm
Haemato-endothelial progenitors
Rostral neuroectoderm
Surface ectoderm
Def. endoderm
g
E7.0 single cells
Epiblast
Primitive Streak
Caudal epiblast
PGC
Anterior Primitive Streak
Def. endoderm
Gut
Nascent mesoderm
Mixed mesoderm
Mesenchyme
Haematoendothelial progenitors
Blood progenitors 1
Blood progenitors 2
Rostral neurectoderm
Surface ectoderm
Visceral endoderm
h
E7.0
Epiblast
Primitive Streak
Caudal epiblast
PGC
Anterior Primitive Streak
Def. endoderm
Gut
Nascent mesoderm
Blood progenitors 2
Rostral neurectoderm
Surface ectoderm
Visceral endoderm
Mixed mesoderm
Mesenchyme
Haematoendothelial progenitors
Blood progenitors 1

Panel 2

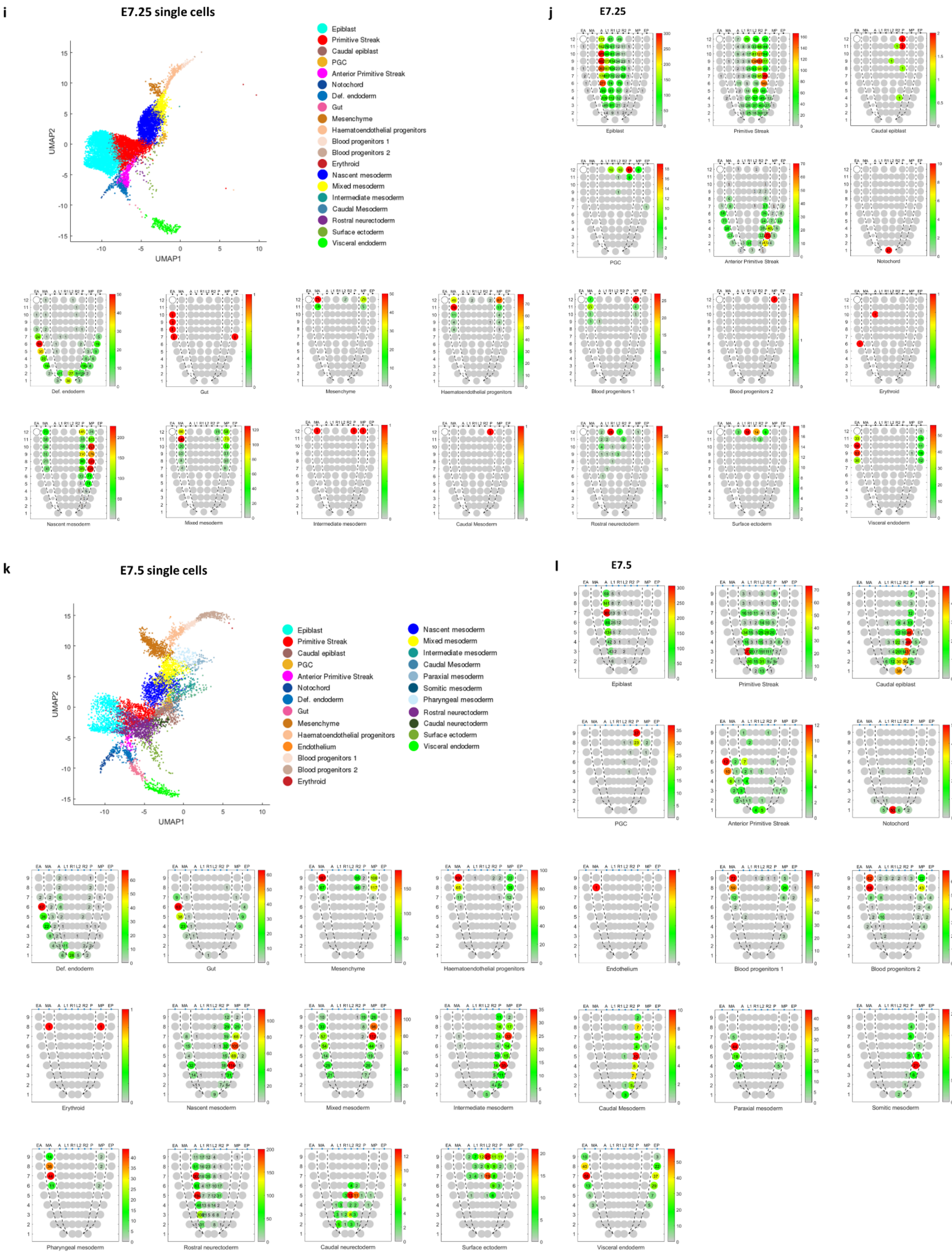

i
E7.25 single cells
UMAP1
UMAP2
Epiblast
Primitive Streak
Caudal epiblast
PGC
Anterior Primitive Streak
Notochord
Def. endoderm
Gut
Mesenchyme
Haematoendothelial progenitors
Blood progenitors 1
Blood progenitors 2
Erythroid
Nascent mesoderm
Mixed mesoderm
Intermediate mesoderm
Caudal Mesoderm
Rostral neurectoderm
Surface ectoderm
Visceral endoderm
j
E7.25
k
E7.5 single cells
UMAP1
UMAP2
Epiblast
Primitive Streak
Caudal epiblast
PGC
Anterior Primitive Streak
Notochord
Def. endoderm
Gut
Mesenchyme
Haematoendothelial progenitors
Endothelium
Blood progenitors 1
Blood progenitors 2
Erythroid
Nascent mesoderm
Mixed mesoderm
Intermediate mesoderm
Caudal Mesoderm
Paraxial mesoderm
Somitic mesoderm
Pharyngeal mesoderm
Rostral neurectoderm
Caudal neurectoderm
Surface ectoderm
Visceral endoderm
l
E7.5

Supplementary Figure 4. 3D model for displaying transcriptome data and the verification of MDSC Mapping results.

a. 3D corn plots showing the spatio-temporal pattern of expression of *T* and *Otx2* in epiblast/ectoderm layer of E6.5-E7.5 embryos.

b. Verification of the results of MDSC Mapping of single cells isolated from known positions of E6.5, E6.75, E7.25 and E7.5 embryos. The number on each corn indicates the number of cells mapped to the specific position in the germ layers. PCC values and confidence intervals shown in the simulation.

c-l. Uniform manifold approximation and projection (UMAP) plots (**c, e, g, i, k**) showing the data structure of single cells identified in the 'Gastrulation Atlas' and MDSC Mapping results (**d, f, h, j, l,**) for E6.5 (**c, d**), E6.75 (**e, f**), E7.0 (**g, h**), E7.25 (**i, j**) and E7.5 (**k, l**) embryos. Cell types are annotated (legend of UMAP) and the spatial distribution of each annotated cell types is displayed in corn plots, with the number of cells mapped to specific Geo-seq position (number in the corn) shown.

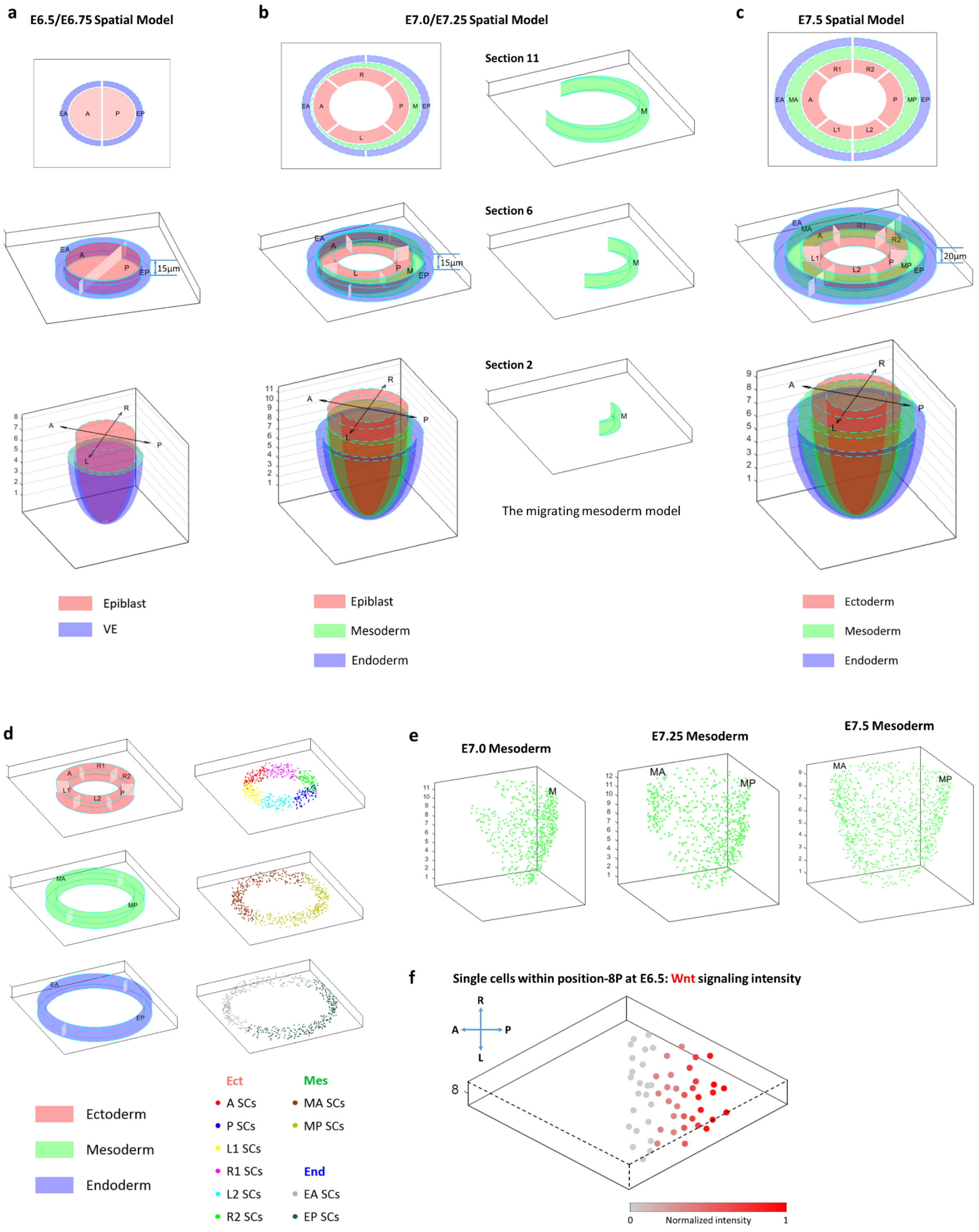
a
E6.5/E6.75 Spatial Model
EA
A
P
EP
15µm
R
L
Epiblast
VE
b
E7.0/E7.25 Spatial Model
R
A
P
M
EP
15µm
Section 11
Section 6
Section 2
The migrating mesoderm model
Epiblast
Mesoderm
Endoderm
c
E7.5 Spatial Model
R1
R2
EA
MA
A
P
MP
EP
L1
L2
20µm
Ectoderm
Mesoderm
Endoderm
d
Ectoderm
Mesoderm
Endoderm
Ect
A SCs
P SCs
L1 SCs
R1 SCs
L2 SCs
R2 SCs
Mes
MA SCs
MP SCs
End
EA SCs
EP SCs
e
E7.0 Mesoderm
E7.25 Mesoderm
E7.5 Mesoderm
f
Single cells within position-8P at E6.5: Wnt signaling intensity
Normalized intensity
0
1

Supplementary Figure 5. 3D modeling for single-cell resolution embryo map.

a. The 3D model of E6.5 and E6.75 embryos. Based on Geo-seq sampling strategy, semicircular pattern for the epiblast and Annulus Model for the endoderm were devised to display the spatial distribution of single cells. Section thickness, 15 μm.

b. The Annulus Model of the epiblast and endoderm of E7.0 and E7.25 embryos. At E7.0-E7.25, the Migration Model mirrors the mesoderm layer spanning from posterior to anterior of the embryo. Section thickness, 15 μm.

c. The Annulus Model of E7.5 embryo. Concentric annuli represent the ectoderm, mesoderm and endoderm from the inside outward. Section thickness, 20 μm.

d. The display of single cells in the three germ layers of E7.5 embryo. Single cells that mapped to a Geo-seq position were distributed uniformly across the corresponding interior space of each domain in the annulus section.

e. The display of single cells in the Migration Model of the mesoderm of E7.0-E7.5 embryo.

f. Bubble Sort algorithm re-ordered the distribution pattern of single cells in E6.5 position-8P by graded WNT activity in the anterior-posterior axis. The color legend indicates the normalized expression level determined by averaged transcript counts.

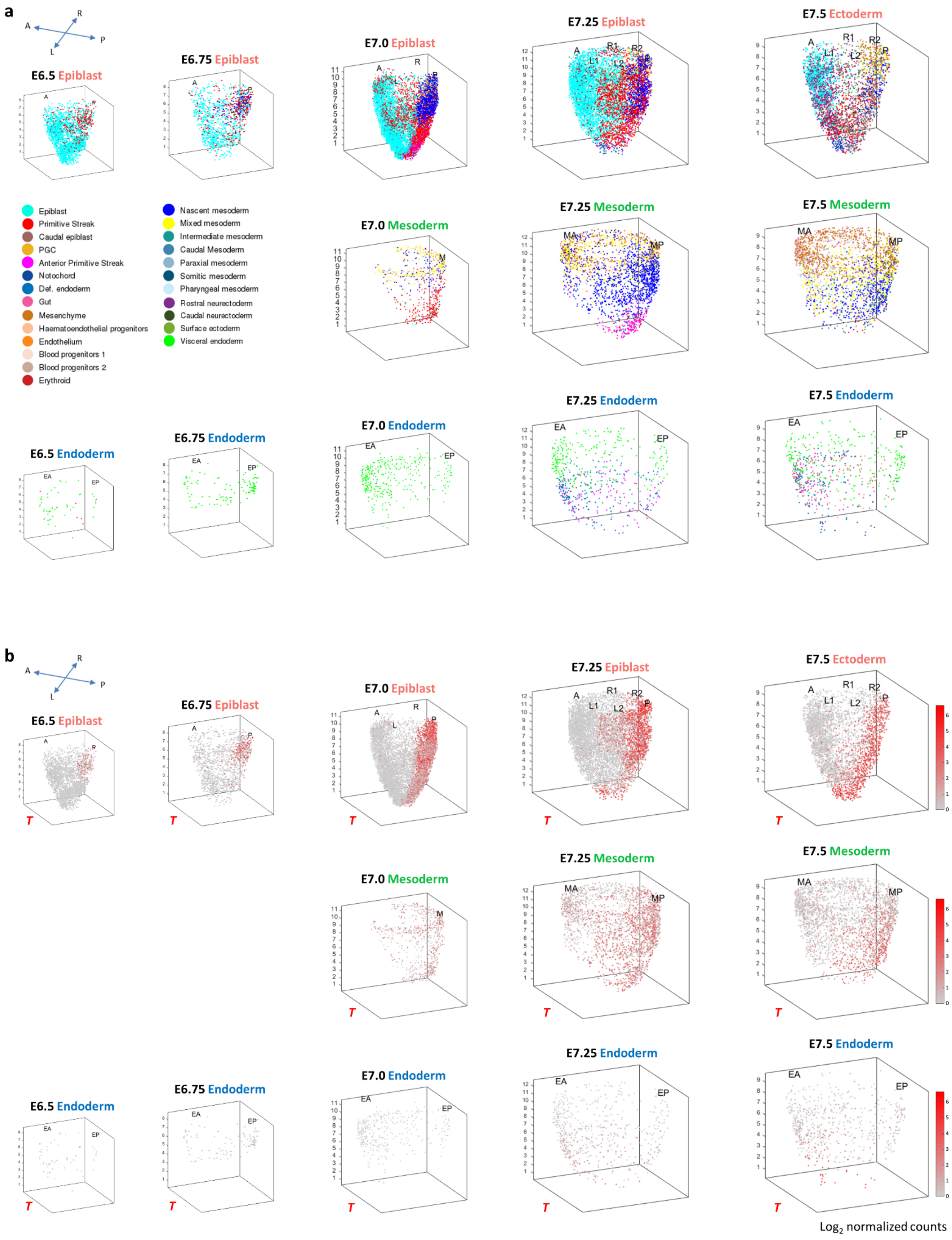

a
A
R
P
L
E6.5 Epiblast
E6.75 Epiblast
E7.0 Epiblast
E7.25 Epiblast
E7.5 Ectoderm
E7.0 Mesoderm
E7.25 Mesoderm
E7.5 Mesoderm
E6.5 Endoderm
E6.75 Endoderm
E7.0 Endoderm
E7.25 Endoderm
E7.5 Endoderm
L1
L2
R1
R2
MA
MP
M
EA
EP
Epiblast
Primitive Streak
Caudal epiblast
PGC
Anterior Primitive Streak
Notochord
Def. endoderm
Gut
Mesenchyme
Haematoendothelial progenitors
Endothelium
Blood progenitors 1
Blood progenitors 2
Erythroid
Nascent mesoderm
Mixed mesoderm
Intermediate mesoderm
Caudal Mesoderm
Paraxial mesoderm
Somitic mesoderm
Pharyngeal mesoderm
Rostral neurectoderm
Caudal neurectoderm
Surface ectoderm
Visceral endoderm
b
T
Log2 normalized counts

Supplementary Figure 6. The spatio-temporal distribution of single cells in E6.5-E7.5 mouse embryos.

a. The spatio-temporal distribution of all the single cells identified in the 'Gastrulation Atlas' in the epiblast/ectoderm, mesoderm and endoderm of E6.5-E7.5 mouse embryos.

b. The spatio-temporal distribution of *T*-expressing cells in E6.5-E7.5 embryos. The color legend indicates the level of expression determined by transcript counts.

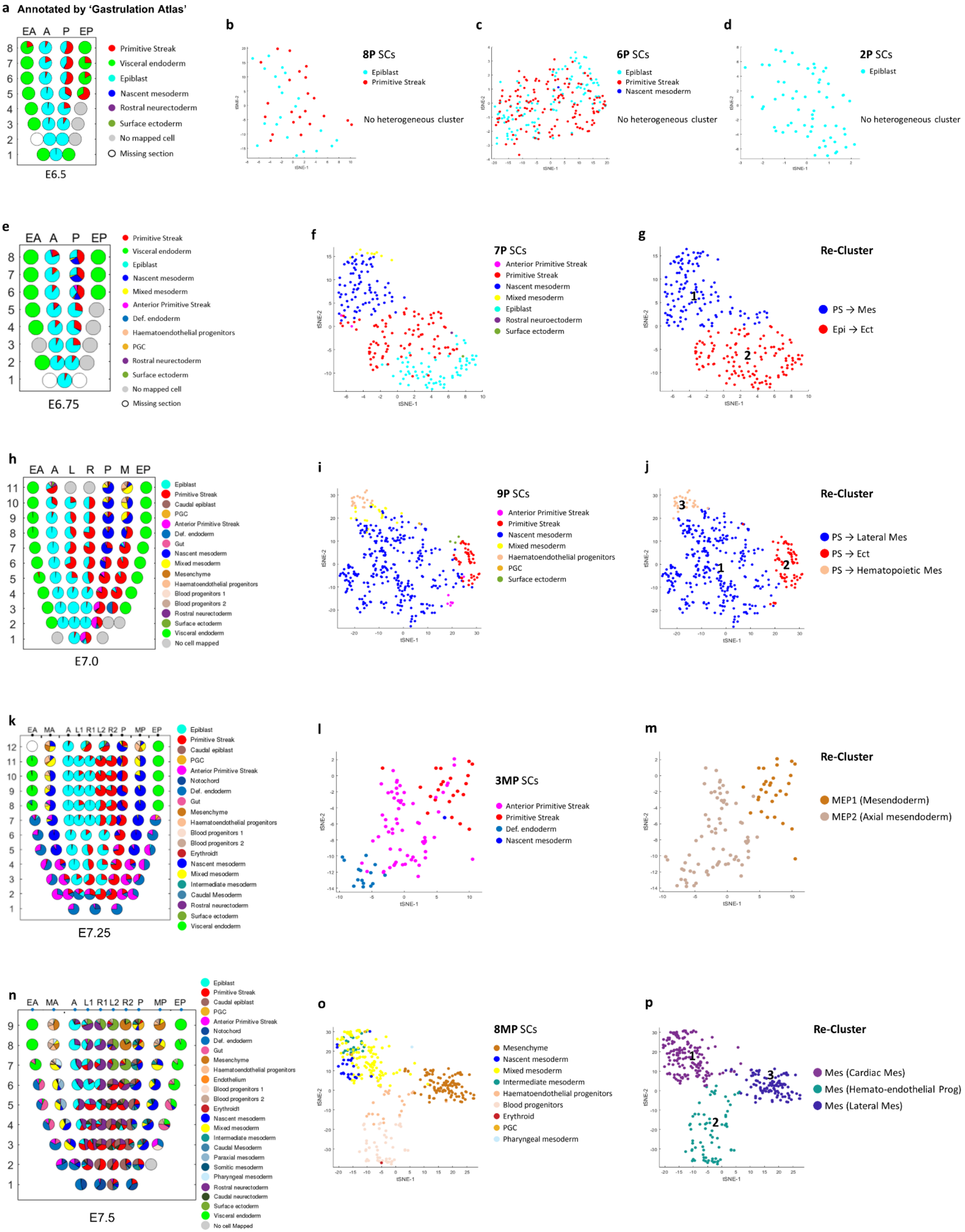
a
Annotated by 'Gastrulation Atlas'
E6.5
Primitive Streak
Visceral endoderm
Epiblast
Nascent mesoderm
Rostral neurectoderm
Surface ectoderm
No mapped cell
Missing section
b
8P SCs
No heterogeneous cluster
c
6P SCs
No heterogeneous cluster
d
2P SCs
No heterogeneous cluster
e
E6.75
f
7P SCs
g
Re-Cluster
PS → Mes
Epi → Ect
h
E7.0
i
9P SCs
j
Re-Cluster
PS → Lateral Mes
PS → Ect
PS → Hematopoietic Mes
k
E7.25
l
3MP SCs
m
Re-Cluster
MEP1 (Mesendoderm)
MEP2 (Axial mesendoderm)
n
E7.5
o
8MP SCs
p
Re-Cluster
Mes (Cardiac Mes)
Mes (Hemato-endothelial Prog)
Mes (Lateral Mes)

Supplementary Figure 7. Analysis of heterogeneity of single cell population.

a-p. Heterogeneity Map of single cell types annotated by 'Gastrulation Atlas' displayed as pie charts in corn plots (**a, e, h, k, n**) and *t*-SNE plots of single cells (**b, c, d, f, g, i, j, l, m, o, p**) showing examples of heterogeneity of cell types (with revised annotation according to **Supplementary Fig. 8**) in specified Geo-seq positions of E6.5 (**a-d**), E6.75 (**e-g**), E7.0 (**h-j**), E7.25 (**k-m**) and E7.5 (**n-p**) embryos.

Stage	**Gastrulation Atlas** (Pijuan-Sala, B. *et al.*, 2019)	**Geo-seq** (Figure 1a)	**Heterogeneity Map** (Figure 3d, e)	**Cell label**
**E6.5**	Epiblast	Epi	Epi	Epiblast
	Primitive Streak	PS	PSLC	Primitive streak like cells
	Visceral endoderm	E1	E1 (Non-proximal Ve)	Non-proximal visceral endoderm
		E2	E2 (Proximal Ve)	Proximal visceral endoderm
**E6.75**	Epiblast	Epi	Epi	Epiblast
	Primitive streak	PS	Epi → Ect	Posterior epiblast: ectoderm progenitor
			PSLC	Primitive streak like cells
	Nascent mesoderm		PS → Mes	Primitive streak: mesoderm progenitor
	Visceral endoderm	E1	E1 (Non-proximal Ve)	Non-proximal visceral endoderm
		E2	E2 (Proximal Ve)	Proximal visceral endoderm
**E7.0**	Epiblast	Epi1	Epi → Neuroectoderm	Epiblast: neuroectoderm progenitor
		Epi2	Epi → Ect	Epiblast: ectoderm progenitor
		Epi3	Epi → SEct/AmEct	Epiblast: surface ectoderm, amnion ectoderm progenitor
	Primitive streak	PS	PS → Ect	Primitive streak: ectoderm progenitor
			PS → Lateral Mes	Primitive streak: lateral mesoderm progenitor
			PS → Hematopoietic Mes	Primitive streak: hematopoietic mesoderm progenitor
	Anterior primitive streak		PS → Paraxial Mes Prog	Primitive streak: paraxial mesoderm progenitor
			PS (Anterior PS)	Primitive streak: anterior-streak like cells
			PS → Endoderm	Primitive streak: endoderm progenitor
	Nascent mesoderm	M	Mes (Mesoderm Prog)	Mesoderm progenitor
			Mes → Axial mesendoderm	Axial mesendoderm progenitor
	Mixed mesoderm		Mes (Cardiogenic Mes)	Cardiogenic mesoderm
			Mes (Paraxial Mes)	Paraxial mesoderm
	Haematoendothelial progenitors		Mes (Vasculogenic Mes)	Vasculogenic Mesoderm
	Visceral endoderm	E1	E1 (Non-proximal Ve)	Non-proximal visceral endoderm
		E2	E2 (Proximal Ve)	Proximal visceral endoderm
**E7.25**	Epiblast	Epi1	Epi (Neuroectoderm Prog)	Epiblast: neuroectoderm progenitor
			Epi (Ectoderm Prog)	Epiblast: ectoderm progenitor
		Epi2	Epi (Posterior epiblast)	Epiblast: posterior epiblast
			Epi (Surface Ect Prog)	Epiblast: surface ectoderm progenitor
	Primitive streak	PS	PS → ExE Mes	Primitive streak: extraembryonic mesoderm progenitor
			PS → Mesoderm Prog	Primitive streak: mesoderm progenitor
			PS (PSLC)	Primitive streak: primitive streak like cells
			PS → Mesendoderm Prog	Primitive streak: mesendoderm progenitor
	Anterior primitive streak	MEP	MEP1 (Mesendoderm)	MEP1: mesendoderm
			MEP2 (Axial mesendoderm)	MEP2: axial mesendoderm
	Nascent mesoderm	M	Mes (Nascent Mes)	Nascent mesoderm
	Mixed mesoderm		Mes (Paraxial Mes)	Paraxial mesoderm
	Haematoendothelial progenitors		Mes (Hematopoietic Mes)	Hematopoietic mesoderm
	Mesenchyme		Mes (Cardiac Mes)	Cardiac mesoderm
	Visceral endoderm	E1	E1 (Non-proximal Ve)	Non-proximal visceral endoderm
		E2	E2 (Proximal Ve)	Proximal visceral endoderm
	Def. endoderm	E3	E3 (Distal endoderm)	Distal endoderm
**E7.5**	Rostral neuroectoderm	Ect1	Ect (Neuroectoderm)	Ectoderm: neuroectoderm progenitor
	Epiblast	Ect2	Ect (Epiblast)	Ectoderm: epiblast
	Caudal epiblast		Ect (PSLC)	Ectoderm: primitive streak like cells
	Surface ectoderm	Ect3	Ect (Surface Ect)	Ectoderm: surface ectoderm progenitor
	Mesenchyme		Ect (Lateral Mes Prog)	Ectoderm: lateral mesoderm progenitor
	Primitive streak	PS	PS → Lateral Mes	Primitive streak: lateral mesoderm progenitor
			PS → Paraxial Mes	Primitive streak: paraxial mesoderm progenitor
			PS → Mesoderm Prog	Primitive streak: mesoderm progenitor
			PS → Anterior Mes	Primitive streak: anterior mesoderm progenitor
			PS → Upper trunk Mes	Primitive streak: upper trunk mesoderm progenitor
	Notochord		Node1 (Def endoderm)	Definitive endoderm
	Anterior primitive streak		Node2 (Axial mesendoderm)	Axial mesendoderm
	Haematoendothelial/Blood progenitors	M1	Mes (Hemato-endothelial Prog)	Hemato-endothelial progenitor
	Mixed mesoderm		Mes (Cardiac Mes)	Cardiac mesoderm
			Mes (Lateral Mes)	Lateral mesoderm
	Intermediate mesoderm	M2	Mes (Paraxial Mes)	Paraxial mesoderm
	Nascent mesoderm		Mes (Nascent Mes)	Nascent mesoderm
	Visceral endoderm	E1	E1 (Non-proximal Ve)	Non-proximal visceral endoderm
		E2	E2 (Proximal Ve)	Proximal visceral endoderm
	Def. endoderm	E3	E3 (Distal endoderm)	Distal endoderm

Supplementary Figure 8. Re-annotation of cell types.

Revised annotation of single cell types mapped to the Geo-seq position in the epiblast/ectoderm, primitive streak, mesoderm and endoderm. Annotation of cell types in ‘Gastrulation Atlas’ is shown for comparison. The nomenclature ‘X→Y’ and ‘X(Y)’ represent different cell states. X represents the germ layer information of cell population. ‘X→Y’ indicates these cells are representing a transitional cell state from X to Y. And ‘X(Y)’ represents the precursor of a specified cell type (Y) in the germ layer X.

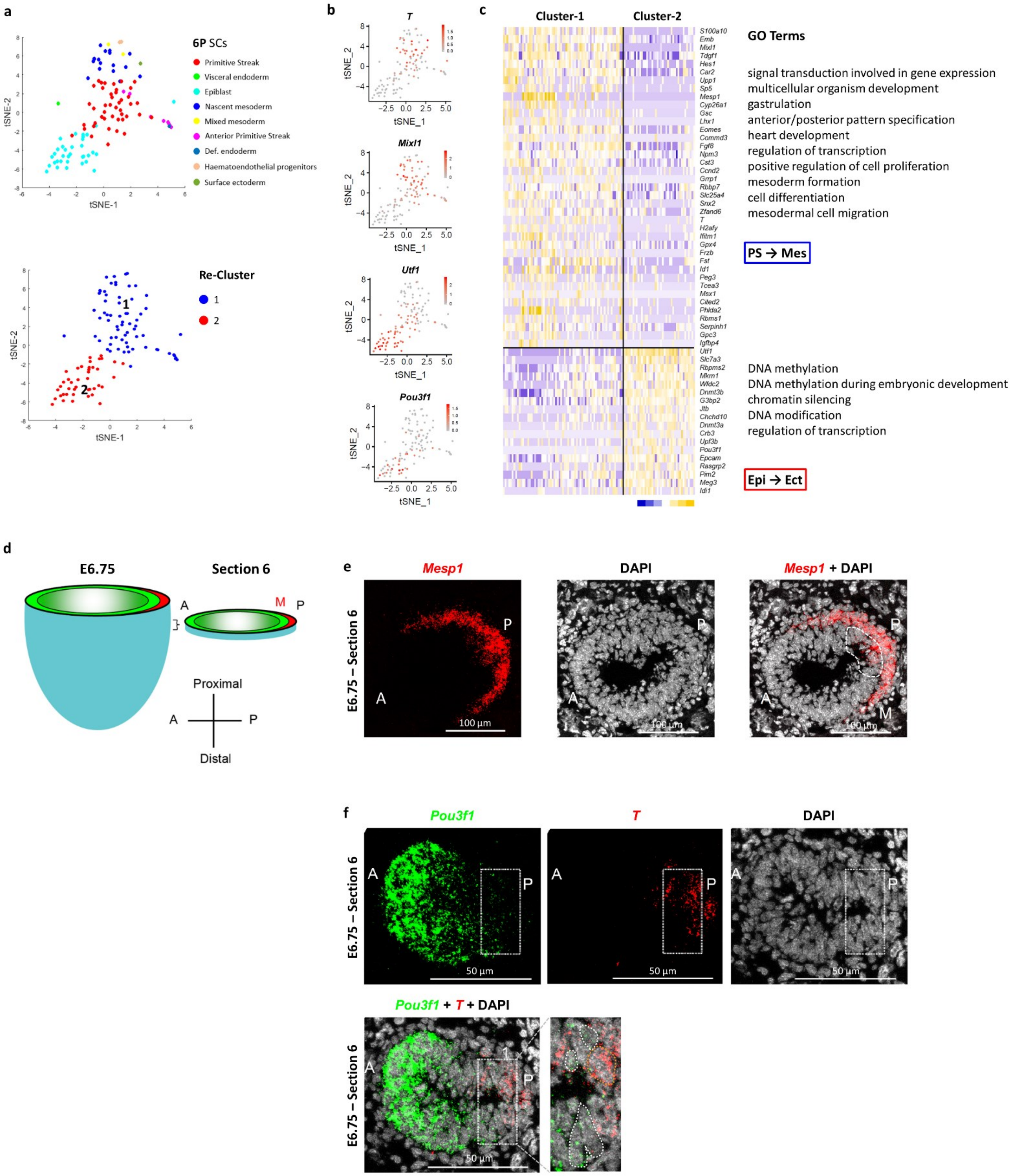

a
6P SCs
Primitive Streak
Visceral endoderm
Epiblast
Nascent mesoderm
Mixed mesoderm
Anterior Primitive Streak
Def. endoderm
Haematoendothelial progenitors
Surface ectoderm
tSNE-1
tSNE-2
Re-Cluster
1
2
b
T
Mixl1
Utf1
Pou3f1
tSNE_1
tSNE_2
c
Cluster-1
Cluster-2
S100a10
Emb
Mixl1
Tdgf1
Hes1
Car2
Upp1
Sp5
Mesp1
Cyp26a1
Gsc
Lhx1
Eomes
Commd3
Fgf8
Npm3
Cst3
Ccnd2
Grrp1
Rbbp7
Slc25a4
Snx2
Zfand6
T
H2afy
Ifitm1
Gpx4
Frzb
Fst
Id1
Peg3
Tcea3
Msx1
Cited2
Phlda2
Rbms1
Serpinh1
Gpc3
Igfbp4
Utf1
Slc7a3
Rbpms2
Mkrn1
Wfdc2
Dnmt3b
G3bp2
Jtb
Chchd10
Dnmt3a
Crb3
Upf3b
Pou3f1
Epcam
Rasgrp2
Pim2
Meg3
Idi1
GO Terms
signal transduction involved in gene expression
multicellular organism development
gastrulation
anterior/posterior pattern specification
heart development
regulation of transcription
positive regulation of cell proliferation
mesoderm formation
cell differentiation
mesodermal cell migration
PS → Mes
DNA methylation
DNA methylation during embryonic development
chromatin silencing
DNA modification
regulation of transcription
Epi → Ect
d
E6.75
Section 6
A
M
P
Proximal
Distal
e
Mesp1
DAPI
Mesp1 + DAPI
E6.75 – Section 6
100 µm
f
Pou3f1
T
DAPI
50 µm
Pou3f1 + T + DAPI

Supplementary Figure 9. Heterogeneity analysis and validation of marker genes.

a. *t*-SNE plot showing the single cells mapped to position-6P at E6.75 (top panel). Re-clustering revealed the presence of two distinct clusters (bottom panel).

b. *t*-SNE plot showing the expression pattern of *T* and *Mixl1* for cluster-1, and *Utf1* and *Pou3f1* for cluster-2.

c. Heat map showing the differentially expressed genes of the two cell clusters ($p < 0.01$, fold change > 1.5). The enriched gene ontology (GO) terms ($p < 0.05$) provided additional information for annotating Cluster-1 as ‘PS→Mes’ and Cluster-2 as ‘PS →Ect’.

d. RNAscope analysis for E6.75 embryo.

e. RNAscope analysis validated the expression of *Mesp1* in the posterior epiblast of E6.75 embryo.

f. RNAscope analysis validated the co-localization of *T* and *Pou3f1* at the posterior epiblast of E6.75 embryo.

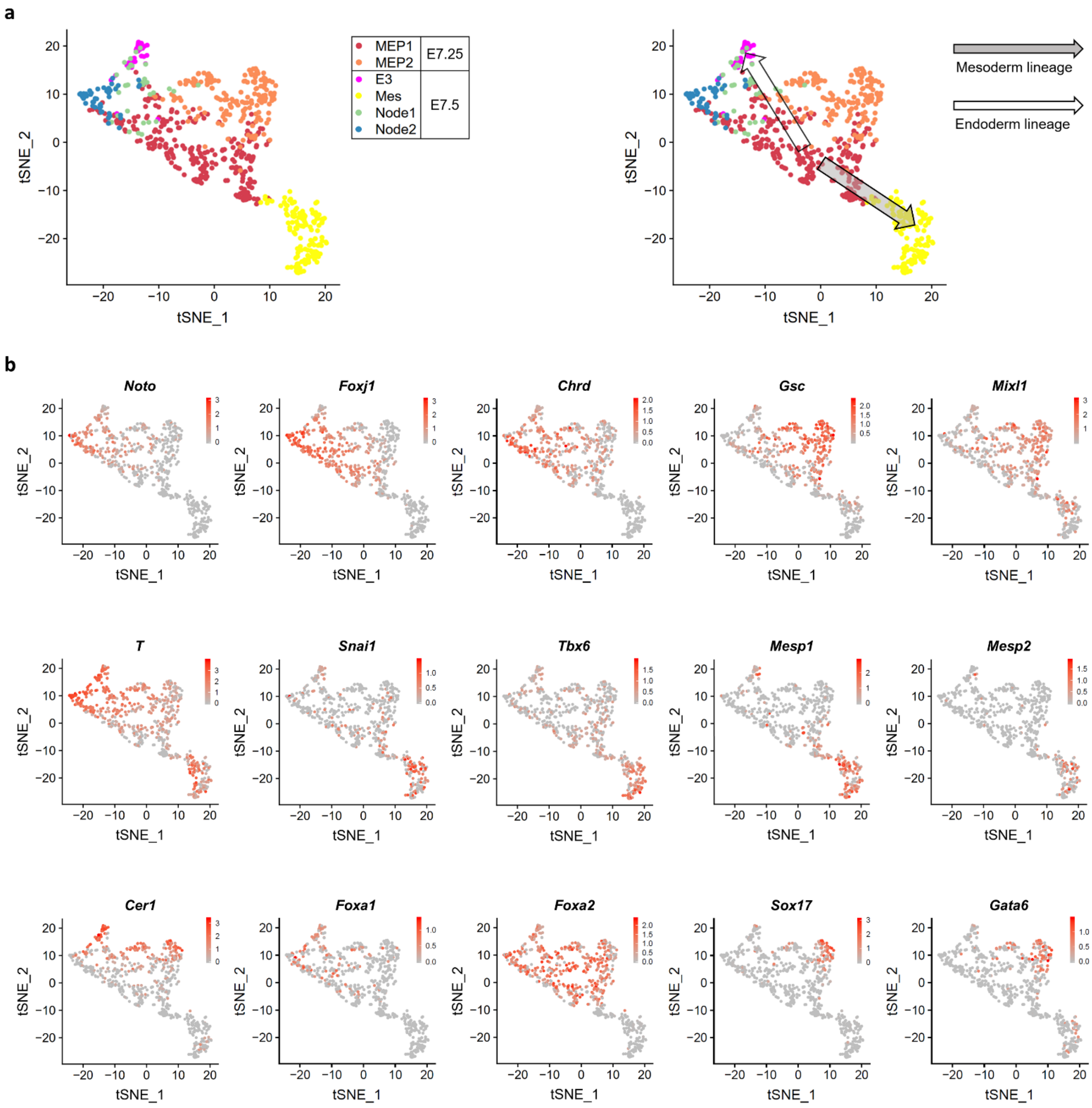
a
MEP1
MEP2
E7.25
E3
Mes
Node1
Node2
E7.5
tSNE_1
tSNE_2
Mesoderm lineage
Endoderm lineage
b
Noto
Foxj1
Chrd
Gsc
Mixl1
T
Snai1
Tbx6
Mesp1
Mesp2
Cer1
Foxa1
Foxa2
Sox17
Gata6

Supplementary Figure 10. Imputation of molecular trajectories of MEP.

a. *t*-SNE plots showing the E7.25 putative MEPs and the inferred E7.5 derivatives. Left panel: annotated cell types, right panel: developmental trajectory.

b. *t*-SNE plots showing the expression pattern of marker genes of node (top panels), mesoderm (middle panels) and endoderm (bottom panels). The color legend indicates the level of expression determined by transcript counts.

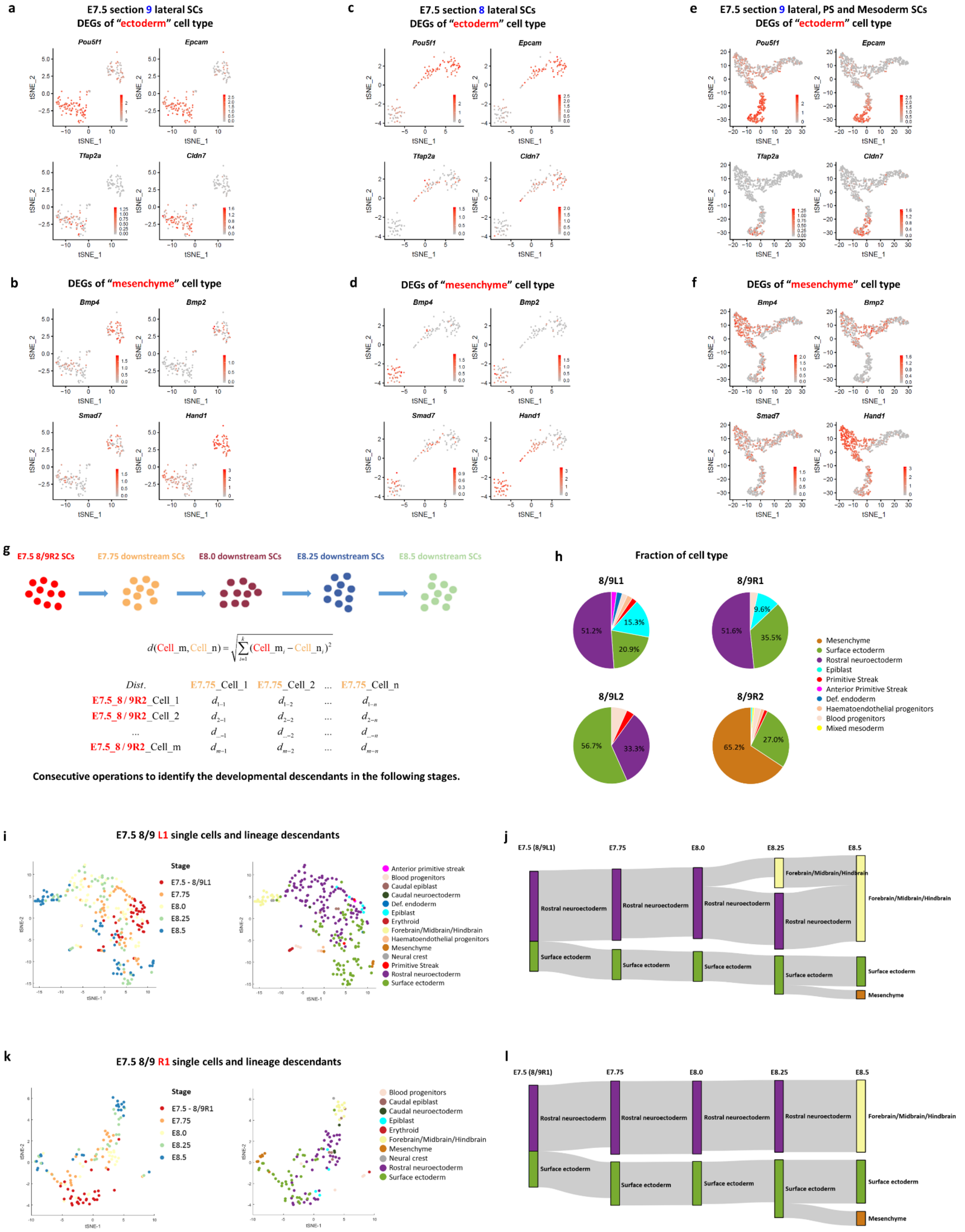

a
E7.5 section 9 lateral SCs
DEGs of "ectoderm" cell type
Pou5f1
Epcam
Tfap2a
Cldn7
tSNE_1
tSNE_2
b
DEGs of "mesenchyme" cell type
Bmp4
Bmp2
Smad7
Hand1
c
E7.5 section 8 lateral SCs
DEGs of "ectoderm" cell type
d
DEGs of "mesenchyme" cell type
e
E7.5 section 9 lateral, PS and Mesoderm SCs
DEGs of "ectoderm" cell type
f
DEGs of "mesenchyme" cell type
g
E7.5 8/9R2 SCs
E7.75 downstream SCs
E8.0 downstream SCs
E8.25 downstream SCs
E8.5 downstream SCs
$d(\text{Cell\_m}, \text{Cell\_n}) = \sqrt{\sum_{i=1}^{k} (\text{Cell\_m}_i - \text{Cell\_n}_i)^2}$
Dist. | E7.75_Cell_1 | E7.75_Cell_2 | ... | E7.75_Cell_n
E7.5_8/9R2_Cell_1 | d_{1-1} | d_{1-2} | ... | d_{1-n}
E7.5_8/9R2_Cell_2 | d_{2-1} | d_{2-2} | ... | d_{2-n}
... | $d_{...-1}$ | $d_{...-2}$ | ... | $d_{...-n}$
E7.5_8/9R2_Cell_m | d_{m-1} | d_{m-2} | ... | d_{m-n}
Consecutive operations to identify the developmental descendants in the following stages.
h
Fraction of cell type
8/9L1
51.2%
15.3%
20.9%
8/9R1
51.6%
9.6%
35.5%
8/9L2
56.7%
33.3%
8/9R2
65.2%
27.0%
Mesenchyme
Surface ectoderm
Rostral neuroectoderm
Epiblast
Primitive Streak
Anterior Primitive Streak
Def. endoderm
Haematoendothelial progenitors
Blood progenitors
Mixed mesoderm
i
E7.5 8/9 L1 single cells and lineage descendants
Stage
E7.5 - 8/9L1
E7.75
E8.0
E8.25
E8.5
Anterior primitive streak
Blood progenitors
Caudal epiblast
Caudal neuroectoderm
Def. endoderm
Epiblast
Erythroid
Forebrain/Midbrain/Hindbrain
Haematoendothelial progenitors
Mesenchyme
Neural crest
Primitive Streak
Rostral neuroectoderm
Surface ectoderm
j
E7.5 (8/9L1)
E7.75
E8.0
E8.25
E8.5
Rostral neuroectoderm
Surface ectoderm
Forebrain/Midbrain/Hindbrain
Mesenchyme
k
E7.5 8/9 R1 single cells and lineage descendants
Stage
E7.5 - 8/9R1
E7.75
E8.0
E8.25
E8.5
Blood progenitors
Caudal epiblast
Caudal neuroectoderm
Epiblast
Erythroid
Forebrain/Midbrain/Hindbrain
Mesenchyme
Neural crest
Rostral neuroectoderm
Surface ectoderm
l
E7.5 (8/9R1)
E7.75
E8.0
E8.25
E8.5
Rostral neuroectoderm
Surface ectoderm
Forebrain/Midbrain/Hindbrain
Mesenchyme

Supplementary Figure 11. Imputation of molecular trajectories of proximal-lateral ectoderm.

a-f. *t*-SNE plots showing the differentially expressed genes of “ectoderm” cell type (**a, c, e**) and “mesenchyme” cell type (**b, d, f**) in position-8R2/9R2 (corresponding to **Fig. 4a-f**). Panel **a-d** showed the single cells of proximal-lateral ectoderm positions in section-9 (**a**, **b**) and -8 (**c**, **d**). Panel **e**, **f** showed the single cells of proximal-lateral ectoderm, primitive streak and mesoderm positions in section-9.

g. Schematics of Population Tracing algorithm for single cells of E7.5-E8.5 embryos.

h. Pie charts showing the fraction of cell types at position-8L1/9L1, 8R1/9R1, 8L2/9L2 and 8R2/9R2.

i-l. *t*-SNE plots (**i, k**) and the molecular trajectories (**j, l**) of single cells (imputed using the Population Tracing algorithm) at position-8L1/9L1 (**i, j**) and position-8R1/9R1 (**k**, **l**) of E7.5 embryo and cells in E7.75-E8.5 embryos. Developmental timepoints (stage) and cell types (see legend of panel **i**, **k**) are indicated in the *t*-SNE plots. Cell types in E7.75-E8.5 embryos are annotated according to the ‘Gastrulation Atlas’.

a

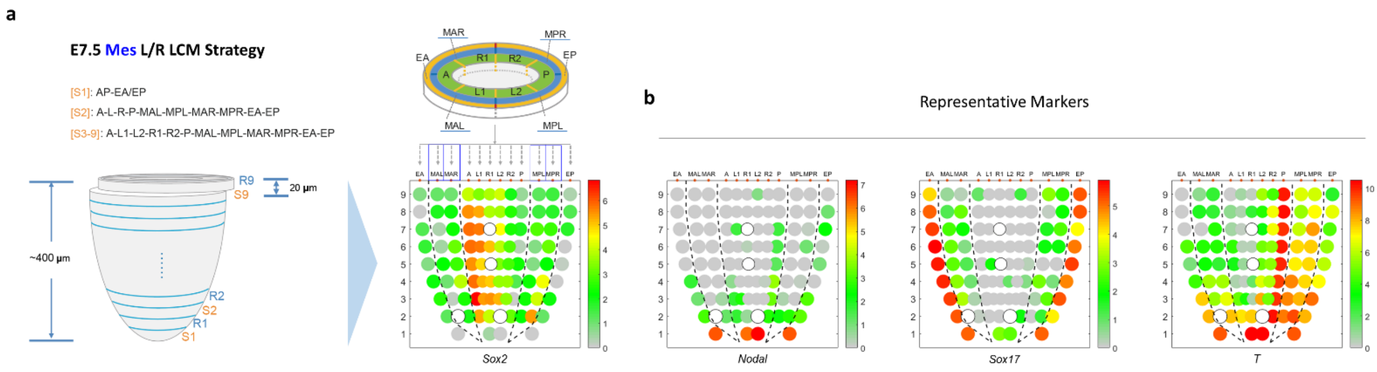

c

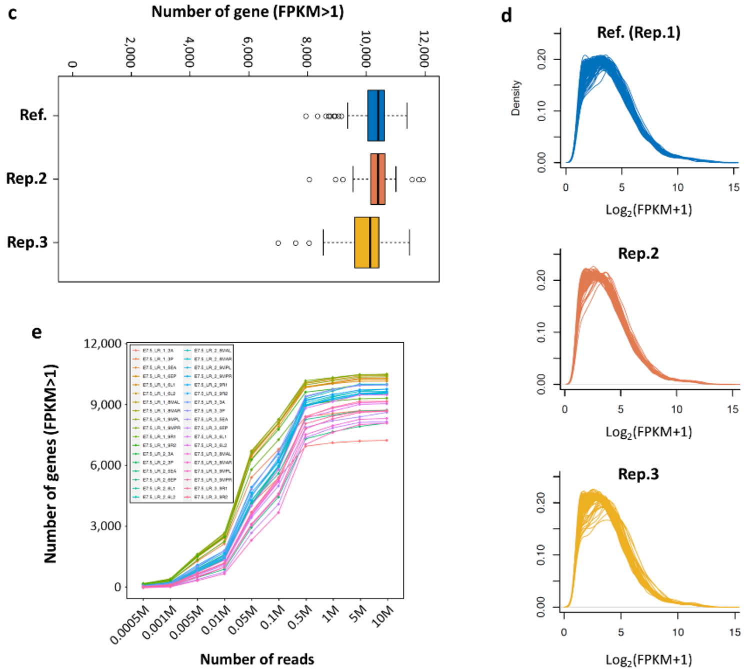

Replicate	Germ layer cells	Geo-seq strategy for mesoderm	Sequencing depth
Ref. (Rep.1)	Ect, Mes, End	MAL, MAR, MPL, MPR	~ 15 M reads per sample
Rep.2	Ect, Mes, End	MAL, MAR, MPL, MPR	~ 20 M reads per sample
Rep.3	Ect, Mes, End	MAL, MAR, MPL, MPR	~ 20 M reads per sample

f

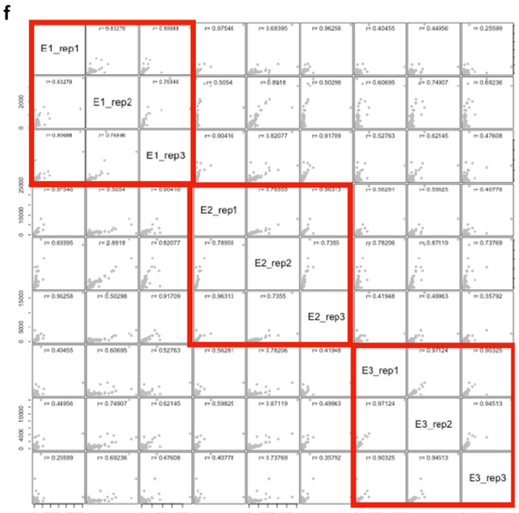

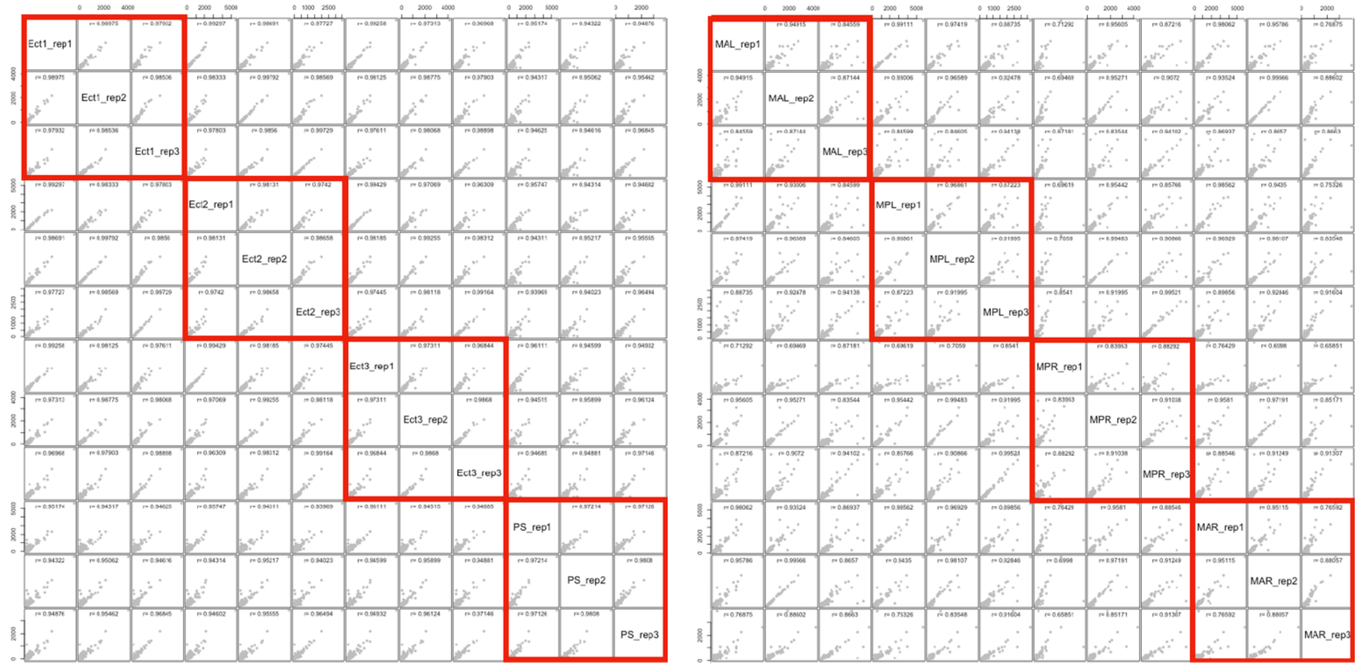

Supplementary Figure 12. Refined Geo-seq analysis of the mesoderm cell layer of the E7.5 embryo.

a. The strategy of sampling cell populations in the E7.5 embryo and the presentation of the spatial pattern of gene expression in the corn plot. MAL, anterior left mesoderm; MAR, anterior right mesoderm; MPL, posterior left mesoderm; MPR, posterior right mesoderm.

b. Corn plots showing the spatial pattern of expression of representative marker genes: *Nodal*, *Sox17*, *T*. Hollow circles indicate missing samples. Table: Geo-seq strategy and sequencing depth for biological replicates.

c. Box plot showing the number of detected genes (FPKM > 1) in the 3 biological replicates. The center line marks the median and box edges represent 25th and 75th percentiles. The median genes detected per replicate is 10,358 (Ref.), 10,389 (Rep. 2) and 10,042 (Rep. 3).

d. Gene expression density plot of Geo-seq data of the 3 biological replicates.

e. Saturation analysis. Different numbers of reads were selected, and the number of detected genes was plotted.

f. Inter-embryo correlation of spatial transcriptome data. The Pearson Correlation Coefficient (PCC) of spatial domains between biological replicates were calculated and showed high inter-embryo consistencies.

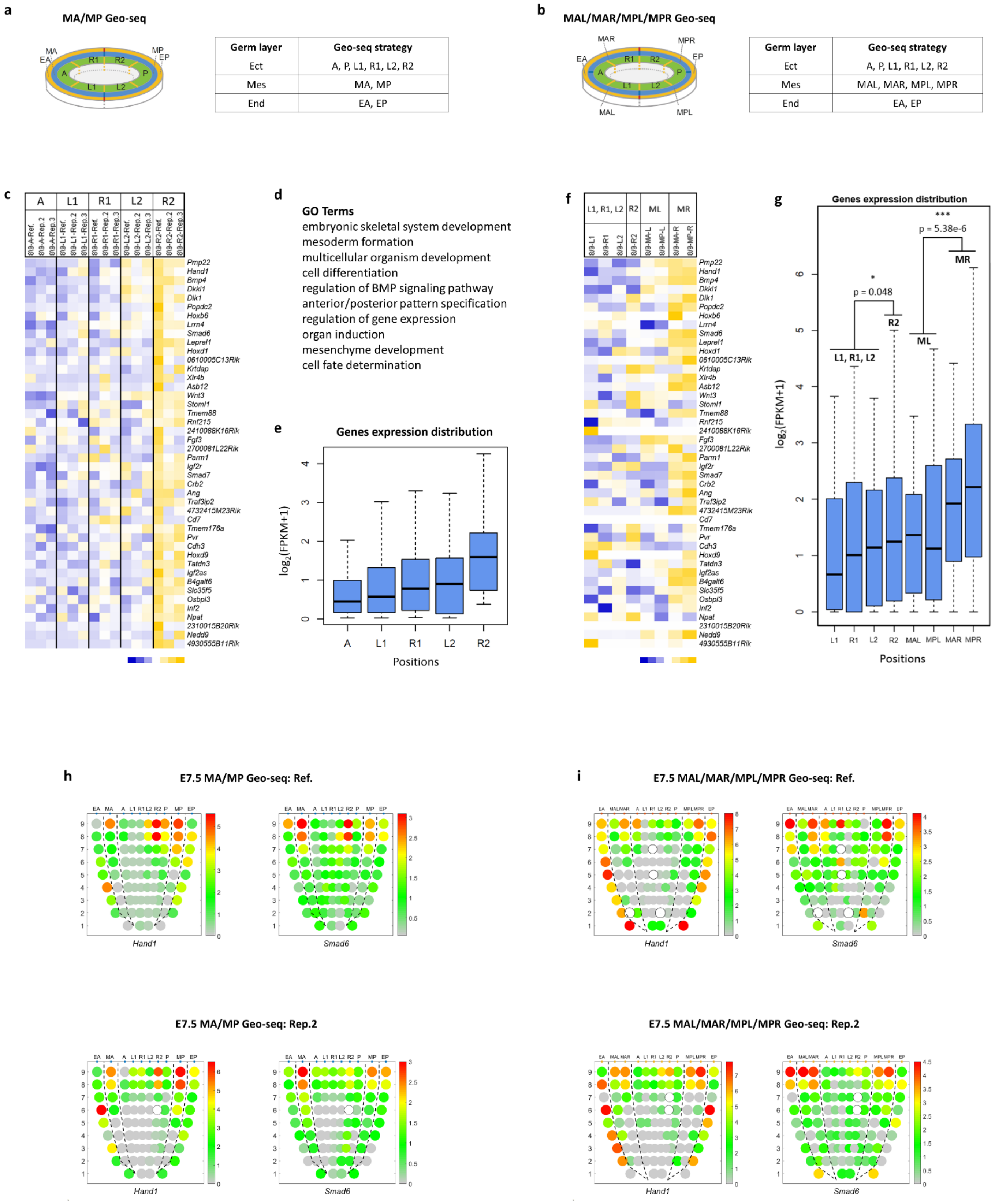

a
MA/MP Geo-seq
MA
EA
R1
R2
MP
EP
A
P
L1
L2
Germ layer | Geo-seq strategy
Ect | A, P, L1, R1, L2, R2
Mes | MA, MP
End | EA, EP
b
MAL/MAR/MPL/MPR Geo-seq
MAR
MPR
EA
EP
MAL
MPL
Germ layer | Geo-seq strategy
Ect | A, P, L1, R1, L2, R2
Mes | MAL, MAR, MPL, MPR
End | EA, EP
c
A
L1
R1
L2
R2
Pmp22
Hand1
Bmp4
Dkkl1
Dlk1
Popdc2
Hoxb6
Lrrn4
Smad6
Leprel1
Hoxd1
0610005C13Rik
Krtdap
Xlr4b
Asb12
Wnt3
Stoml1
Tmem88
Rnf215
2410088K16Rik
Fgf3
2700081L22Rik
Parm1
Igf2r
Smad7
Crb2
Ang
Traf3ip2
4732415M23Rik
Cd7
Tmem176a
Pvr
Cdh3
Hoxd9
Tatdn3
Igf2as
B4galt6
Slc35f5
Osbpl3
Inf2
Npat
2310015B20Rik
Nedd9
4930555B11Rik
d
GO Terms
embryonic skeletal system development
mesoderm formation
multicellular organism development
cell differentiation
regulation of BMP signaling pathway
anterior/posterior pattern specification
regulation of gene expression
organ induction
mesenchyme development
cell fate determination
e
Genes expression distribution
log2(FPKM+1)
A
L1
R1
L2
R2
Positions
f
L1, R1, L2
R2
ML
MR
g
Genes expression distribution
***
p = 5.38e-6
*
p = 0.048
MR
R2
ML
L1, R1, L2
log2(FPKM+1)
L1
R1
L2
R2
MAL
MPL
MAR
MPR
Positions
h
E7.5 MA/MP Geo-seq: Ref.
Hand1
Smad6
E7.5 MA/MP Geo-seq: Rep.2
Hand1
Smad6
i
E7.5 MAL/MAR/MPL/MPR Geo-seq: Ref.
Hand1
Smad6
E7.5 MAL/MAR/MPL/MPR Geo-seq: Rep.2
Hand1
Smad6

Supplementary Figure 13. The right-side DEGs of the proximal-lateral ectoderm showed significantly different expression between the proximal-lateral mesoderm on contralateral sides.

a. The Geo-seq strategy of sampling cells from MA/MP populations in the E7.5 embryo. MA, anterior mesoderm; MP, posterior mesoderm.

b. The Geo-seq strategy of sampling cells from MAL/MAR//MPL/MPR populations in the E7.5 embryo. MAL, anterior left mesoderm; MAR, anterior right mesoderm; MPL, posterior left mesoderm; MPR, posterior right mesoderm.

c. Heatmap showing the differentially expressed genes (DEGs) in 8/9R2 regions compared with other lateral regions (8/9L1, 8/9L2, and 8/9R1) and anterior (A) region in the 3 Geo-seq replicates (Original MA/MP Geo-seq strategy), $p < 0.1$, fold change > 1.5.

d. The enriched gene ontology (GO) terms for the 8/9R2 specific DEGs.

e. Box plot showing the distribution of expression level (Log2 normalized FPKM) of 8/9R2 specific DEGs in different ectoderm regions.

f. Heatmap showing the expression pattern of 8/9R2 specific DEGs (identified in **c**) in different regions of the MAL/MAR/MPL/MPR Geo-seq embryo.

g. Box plot showing the distribution of expression level (Log2 normalized FPKM) of 8/9R2 specific DEGs in different regions of the MAL/MAR/MPL/MPR Geo-seq embryo. T-test p-values were labelled on the box plot. *, $p < 0.05$; **, $p < 0.01$; ***, $p < 0.001$.

h. Corn plots showing the spatial expression pattern of *Hand1* and *Smad6* in the MA/MP Geo-seq embryos. Both reference and replicate embryos show L-R difference in the proximal ectoderm.

i. Corn plots showing the spatial expression pattern of *Hand1* and *Smad6* in the MAL/MAR/MPL/MPR Geo-seq embryos. Both reference and replicate embryos show more significant difference in the right-side proximal lateral mesoderm.

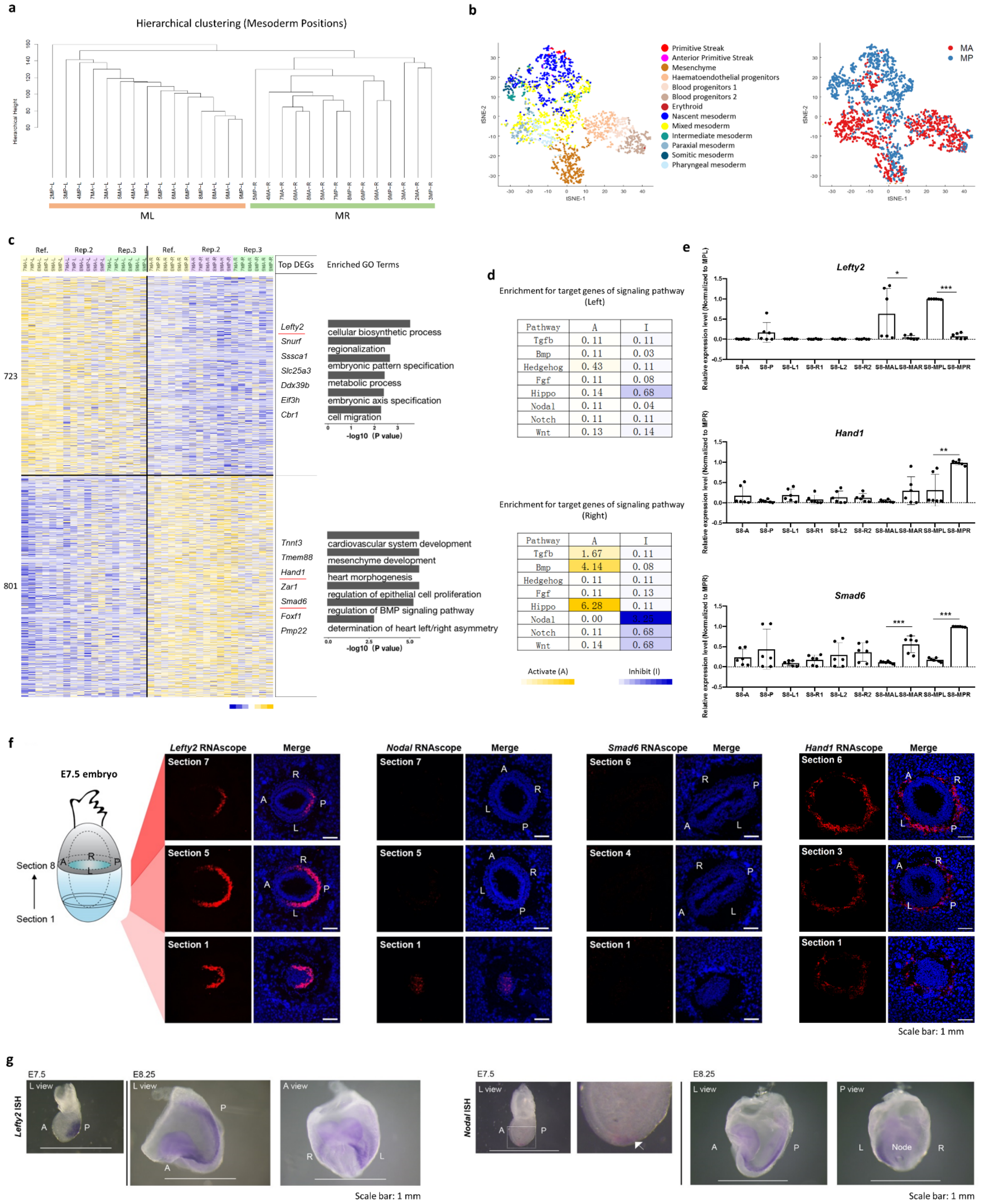

Enrichment for target genes of signaling pathway (Left)

Pathway	A	I
Tgfb	0.11	0.11
Bmp	0.11	0.03
Hedgehog	0.43	0.11
Fgf	0.11	0.08
Hippo	0.14	0.68
Nodal	0.11	0.04
Notch	0.11	0.11
Wnt	0.13	0.14

Enrichment for target genes of signaling pathway (Right)

Pathway	A	I
Tgfb	1.67	0.11
Bmp	4.14	0.08
Hedgehog	0.11	0.11
Fgf	0.11	0.13
Hippo	6.28	0.11
Nodal	0.00	3.25
Notch	0.11	0.68
Wnt	0.14	0.68

Supplementary Figure 14. Validation of the left-right asymmetric gene expression.

a. Hierarchical clustering showed the difference between the mesoderm tissues on contralateral sides of the E7.5 embryo.

b. *t*-SNE plot showing the data structure of single cells that are allocated to mesoderm positions at E7.5. Cells are colored by both their cell-type annotation according to 'Gastrulation Atlas' (left) and anterior/posterior location based on MDSC Mapping results (right).

c. Heat map showing the differentially expressed genes (DEGs) of the proximal-left mesoderm ($n = 723$) and proximal-right mesoderm ($n = 801$) regions across the 3 Geo-seq replicates. The top DEGs and the enriched gene ontology (GO) terms for each group were listed on the right ($p < 0.01$). The genes with red underline were assayed by real-time PCR in **e**.

d. The enrichment for target/response genes of development-related signaling pathways in the proximal-left and proximal-right mesoderm. Signaling activity: red, activating (A); green, inhibitory (I). The significance of $-\log_{10}$(FDR) value in each cell was calculated by one-sided Fisher's exact test followed by Benjamini-Hochberg correction.

e. Real-time PCR showing significant left/right differences of *Lefty2*, *Hand1* and *Smad6* expression in the proximal mesoderm (section 8) of E7.5 embryo. *, $p < 0.05$; **, $p < 0.01$; ***, $p < 0.001$.

f. RNAscope analyses showing the expression of *Lefty2, Nodal, Smad6* and *Hand1* in specified transverse sections (numbered) of E7.5 embryo. These RNAscope analyses serve as negative controls to the results in Fig. 5h. In these sections, there is no discernible difference between lateral sides, except for *Lefty2* in Section 5 and 7. A, anterior; P, posterior; L, left; R, right.

g. Whole-mount in situ hybridization (WISH) showed the laterally asymmetric expression pattern of *Lefty2* (left panel) and *Nodal* (right panel, arrow) in E7.5 and E8.25 mouse embryos. A, anterior; P, posterior; L, left; R, right.

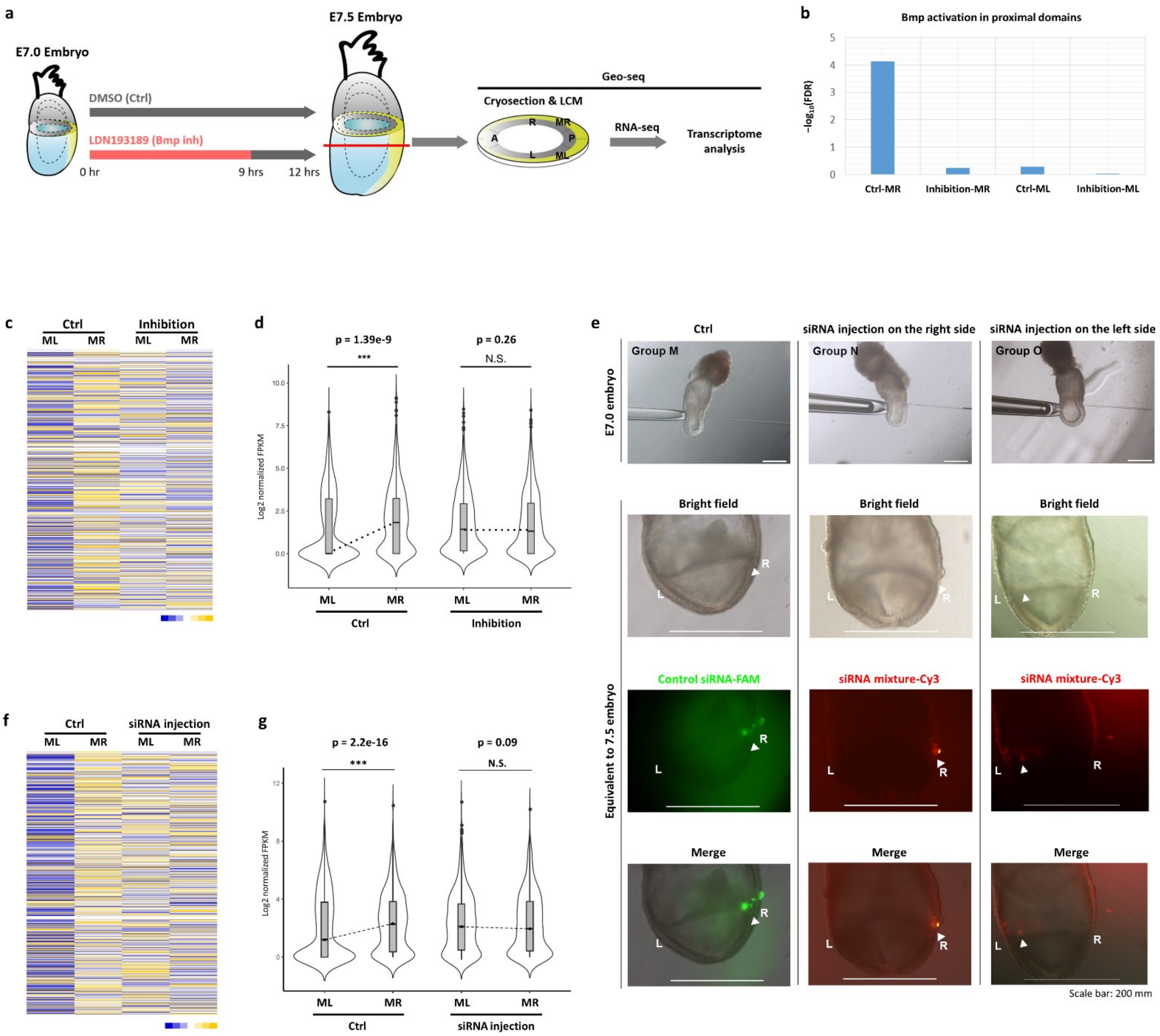

a
E7.0 Embryo
E7.5 Embryo
DMSO (Ctrl)
LDN193189 (Bmp inh)
0 hr
9 hrs
12 hrs
Geo-seq
Cryosection & LCM
R
MR
A
P
L
ML
RNA-seq
Transcriptome analysis
b
Bmp activation in proximal domains
-log10(FDR)
Ctrl-MR
Inhibition-MR
Ctrl-ML
Inhibition-ML
c
Ctrl
Inhibition
ML
MR
d
p = 1.39e-9
***
p = 0.26
N.S.
Log2 normalized FPKM
Ctrl
Inhibition
e
Ctrl
siRNA injection on the right side
siRNA injection on the left side
E7.0 embryo
Group M
Group N
Group O
Bright field
Equivalent to 7.5 embryo
Control siRNA-FAM
siRNA mixture-Cy3
Merge
Scale bar: 200 mm
f
Ctrl
siRNA injection
g
p = 2.2e-16
***
p = 0.09
N.S.
siRNA injection

Supplementary Fig. 15. Geo-seq analyses of the *ex vivo* cultured embryos.

a. Schematic showing the workflow of GEO-seq for *ex vivo* cultured embryos.

b. The BMP signaling activity in the right-side lateral mesoderm region (MR) was curtailed by LDN193189 treatment.

c. Heatmap showing the expression pattern of right-side specific genes of proximal lateral mesoderm (n = 801) in Control (Ctrl) and 9 hours BMP Inhibition embryos. A few right-side specific genes of proximal lateral mesoderm were not expressed in cultured embryos, these genes were then marked with white lines.

d. Violin plot showing the expression level of right-side specific genes of proximal lateral mesoderm in control (Ctrl) and 9 hours BMP Inhibition embryos. The left-right differences were abrogated in the inhibition group. Rank sum test, ***, $p < 0.001$; N.S., no significant difference.

e. Region-specific perturbation of BMP signaling by multiple siRNA knockdowns. Top panel: The experiment snapshot of siRNAs microinjection. Bottom panel: Region-specific transfection of corresponding siRNAs can be observed at embryos of equivalent E7.5 stage.

f. Heatmap showing the expression pattern of right-side specific genes of proximal lateral mesoderm (n = 801) in Control (Ctrl) and siRNA knockdown embryos. A few right-side specific genes of proximal lateral mesoderm were not expressed in cultured embryos, these genes were then marked with white lines.

g. Violin plot showing the expression level of right-side specific genes of proximal lateral mesoderm in control (Ctrl) and siRNA knockdown embryos. The left-right differences were abrogated in the Bmp siRNA microinjection group. Rank sum test, ***, $p < 0.001$; N.S., no significant difference.

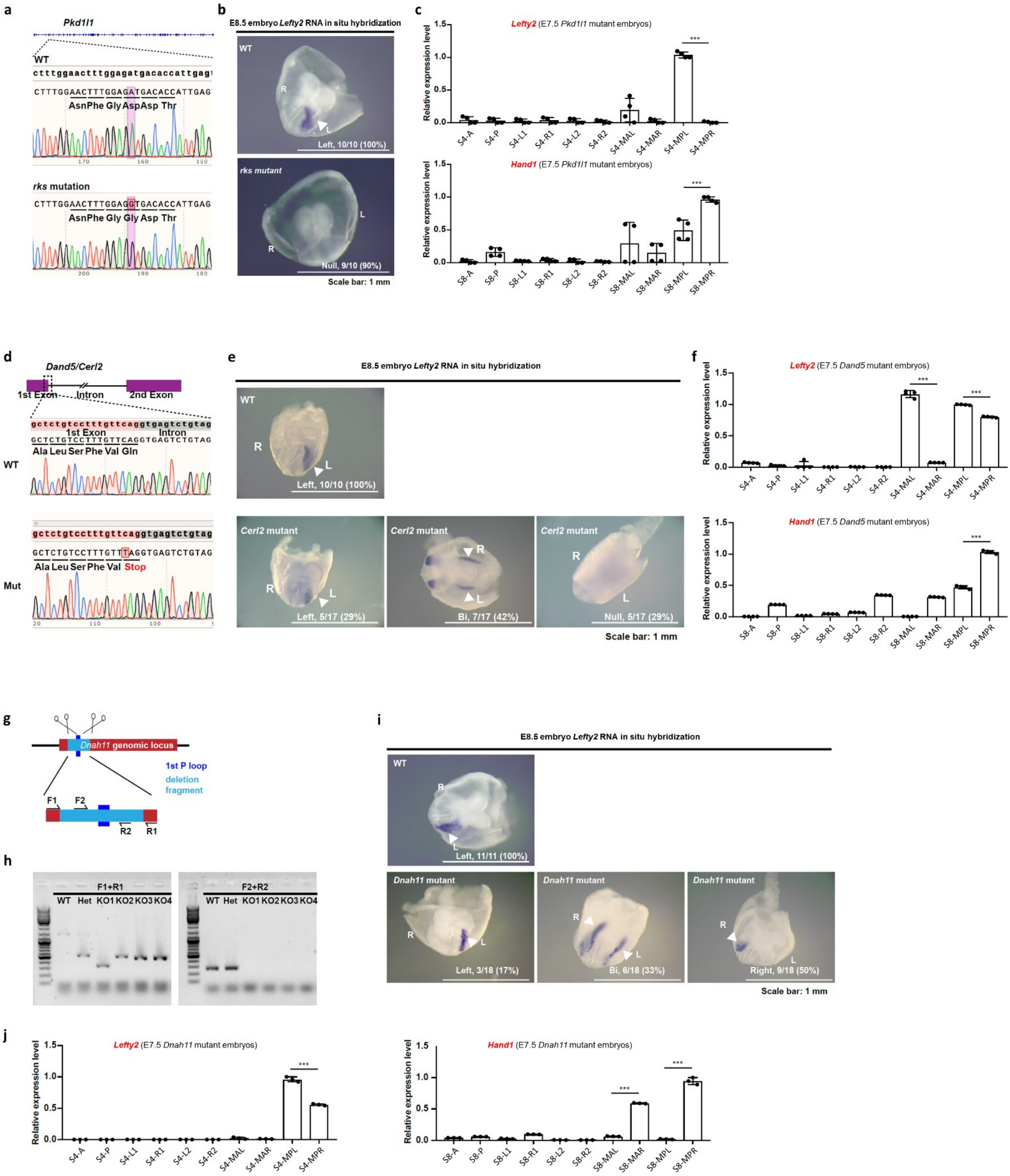
a
Pkd1l1
WT
ctttggaactttggagatgacaccattgag
CTTTGGAACTTTGGAGATGACACCATTGAG
AsnPhe Gly AspAsp Thr
rks mutation
CTTTGGAACTTTGGAGGTGACACCATTGAG
AsnPhe Gly Gly Asp Thr
b
E8.5 embryo Lefty2 RNA in situ hybridization
WT
Left, 10/10 (100%)
rks mutant
Null, 9/10 (90%)
Scale bar: 1 mm
c
Lefty2 (E7.5 Pkd1l1 mutant embryos)
Relative expression level
S4-A
S4-P
S4-L1
S4-R1
S4-L2
S4-R2
S4-MAL
S4-MAR
S4-MPL
S4-MPR
Hand1 (E7.5 Pkd1l1 mutant embryos)
S8-A
S8-P
S8-L1
S8-R1
S8-L2
S8-R2
S8-MAL
S8-MAR
S8-MPL
S8-MPR
d
Dand5/Cerl2
1st Exon
Intron
2nd Exon
gctctgtcctttgttcaggtgagtctgtag
GCTCTGTCCTTTGTTCAGGTGAGTCTGTAG
Ala Leu Ser Phe Val Gln
WT
gctctgtcctttgttcaggtgagtctgtag
GCTCTGTCCTTTGTTTAGGTGAGTCTGTAG
Ala Leu Ser Phe Val Stop
Mut
e
E8.5 embryo Lefty2 RNA in situ hybridization
WT
Left, 10/10 (100%)
Cerl2 mutant
Left, 5/17 (29%)
Bi, 7/17 (42%)
Null, 5/17 (29%)
Scale bar: 1 mm
f
Lefty2 (E7.5 Dand5 mutant embryos)
Hand1 (E7.5 Dand5 mutant embryos)
g
Dnah11 genomic locus
1st P loop
deletion fragment
F1
F2
R2
R1
h
F1+R1
F2+R2
WT Het KO1 KO2 KO3 KO4
i
E8.5 embryo Lefty2 RNA in situ hybridization
WT
Left, 11/11 (100%)
Dnah11 mutant
Left, 3/18 (17%)
Bi, 6/18 (33%)
Right, 9/18 (50%)
Scale bar: 1 mm
j
Lefty2 (E7.5 Dnah11 mutant embryos)
Hand1 (E7.5 Dnah11 mutant embryos)

Supplementary Fig. 16. Asymmetric expression of *Lefty2* and *Hand1* persisted in *Pkd1l1*, *Cerl2* and *Dnah11* mutant embryos.

a-c. *Pkd1l1* mutant embryo: (**a**) Sequencing data of *Pkd1l1* mutant allele. (**b**) Whole-mount in situ hybridization of *Lefty2* in E8.5 mutant embryo. WT, wild type. L, left; R, right. Expression pattern: Null, no expression. (**c**) Real-time PCR showing the relative level of *Lefty2* and *Hand1* expression in E7.5 mutant embryos.

d-f. *Cerl2* (*Dand5*) mutant embryo: (**d**) Sequencing data of *Cerl2* mutant allele. (**e**) Whole-mount in situ hybridization of *Lefty2* in E8.5 mutant embryo. WT, wild type. L, left; R, right. Expression pattern: left; Bi, bilateral; Null, no expression. (**f**) Real-time PCR showing the relative level of *Lefty2* and *Hand1* expression in E7.5 mutant embryos.

g-j. *Dnah11* (*iv*) mutant embryo: (**g**) Schematic of the gene editing strategy. (**h**) Genotyping results of *Dnah11* mutant embryo. (**i**) Whole-mount in situ hybridization of *Lefty2* in E8.5 mutant embryo. WT, wild type. L, left; R, right. Expression pattern: left; Bi, bilateral; Null, no expression. (**j**) Real-time PCR showing the relative level of *Lefty2* and *Hand1* expression in E7.5 mutant embryos.

a E7.5 *Pkd1l1* mutation embryo (3/4 embryos)

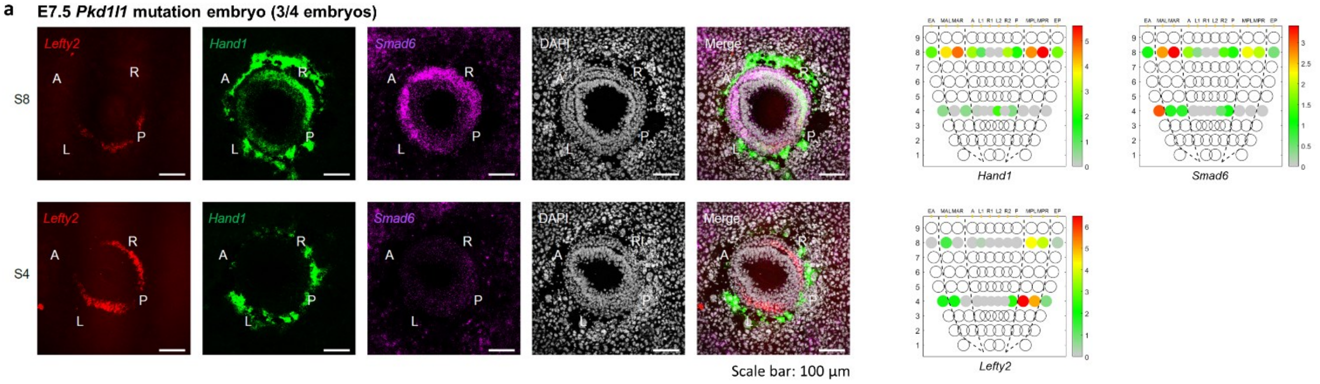

b E7.5 *Cerl2* mutation embryo (4/5 embryos)

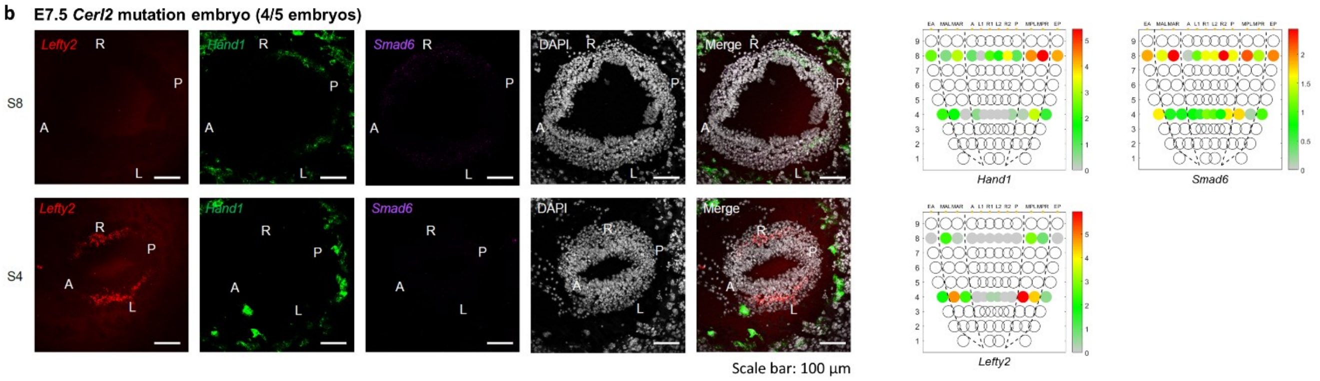

c E7.5 *Dnah11* mutation embryo (3/4 embryos)

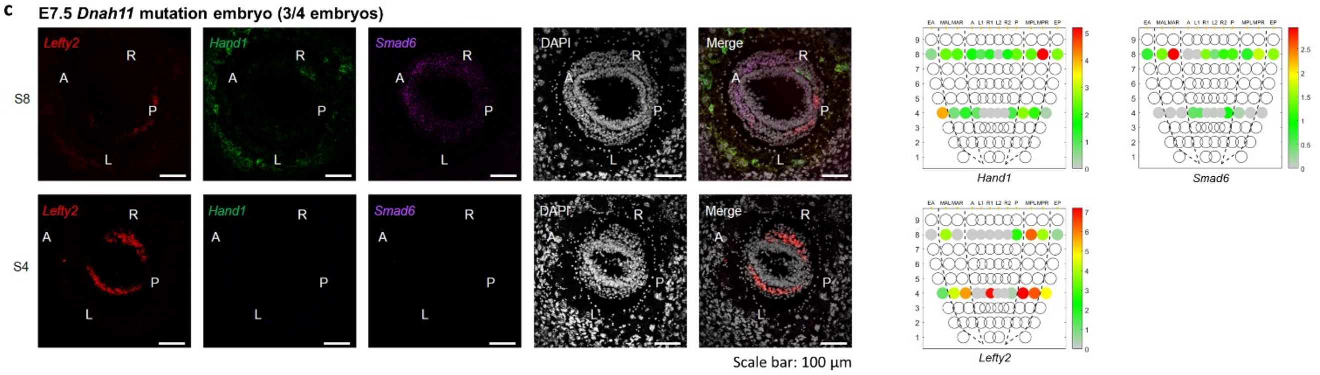

Supplementary Fig. 17. Left-right asymmetry persists in E7.5 mutant embryos.

a-c. Asymmetric pattern of expression of *Lefty2*, *Hand1* and *Smad6* in E7.5 *Pkd1l1* (**a**), *Cerl2* (**b**) and *Dnah11* (**c**) mutant embryos revealed by RNAscope analysis (immunofluorescence images, A, anterior; P, posterior; L, left; R, right) and Geo-seq analysis (corn plots).
