# Supplementary material for: Time space and single-cell resolved tissue lineage trajectories and laterality of body plan at gastrulation
Source: Nat Commun. 2023 Sep 14;14:5675. doi: 10.1038/s41467-023-41482-5 (PMC10502153; doi:10.1038/s41467-023-41482-5)
Supplement: Supplementary file 3 — Description of Additional Supplementary Files [file 41467_2023_41482_MOESM3_ESM.pdf]

Description of Additional Supplementary Files

File Name: Supplementary Data 1
Description: Sequencing depth of Geo-seq data. The number of reads sequenced of each Geo-seq sample.

File Name: Supplementary Data 2
Description: The number of genes detected in each Geo-seq sample.

File Name: Supplementary Data 3
Description: Normalized distance between the spatial domains of the developmental trajectory shown in Fig. 1c.

File Name: Supplementary Data 4
Description: Oligos and primers used in this study.

File Name: Supplementary Data 5
Description: Differentially expressed genes of proximal-left mesoderm.

File Name: Supplementary Data 6
Description: Differentially expressed genes of proximal-right mesoderm.
