# Supplementary material for: Time space and single-cell resolved tissue lineage trajectories and laterality of body plan at gastrulation
Source: Nat Commun. 2023 Sep 14;14:5675. doi: 10.1038/s41467-023-41482-5 (PMC10502153; doi:10.1038/s41467-023-41482-5)
Supplement: Supplementary file 10 — Reporting Summary [file 41467_2023_41482_MOESM10_ESM.pdf]

nature portfolio

Corresponding author(s): Naihe Jing, Patrick P.L. Tam

Last updated by author(s): Aug 11, 2023

Reporting Summary

Nature Portfolio wishes to improve the reproducibility of the work that we publish. This form provides structure for consistency and transparency in reporting. For further information on Nature Portfolio policies, see our Editorial Policies and the Editorial Policy Checklist.

Statistics

For all statistical analyses, confirm that the following items are present in the figure legend, table legend, main text, or Methods section.

n/a	Confirmed	
☐	☒	The exact sample size (*n*) for each experimental group/condition, given as a discrete number and unit of measurement
☐	☒	A statement on whether measurements were taken from distinct samples or whether the same sample was measured repeatedly
☐	☒	The statistical test(s) used AND whether they are one- or two-sided<br>*Only common tests should be described solely by name; describe more complex techniques in the Methods section.*
☒	☐	A description of all covariates tested
☐	☒	A description of any assumptions or corrections, such as tests of normality and adjustment for multiple comparisons
☐	☒	A full description of the statistical parameters including central tendency (e.g. means) or other basic estimates (e.g. regression coefficient) AND variation (e.g. standard deviation) or associated estimates of uncertainty (e.g. confidence intervals)
☐	☒	For null hypothesis testing, the test statistic (e.g. F, t, r) with confidence intervals, effect sizes, degrees of freedom and P value noted<br>*Give P values as exact values whenever suitable.*
☒	☐	For Bayesian analysis, information on the choice of priors and Markov chain Monte Carlo settings
☐	☒	For hierarchical and complex designs, identification of the appropriate level for tests and full reporting of outcomes
☐	☒	Estimates of effect sizes (e.g. Cohen's d, Pearson's r), indicating how they were calculated

Our web collection on statistics for biologists contains articles on many of the points above.

Software and code

Policy information about availability of computer code

Data collection	Fluorescent Images were acquired using Leica TCS SP8 STED system. RT-qPCR data was collected using Eppendorf Realplex mastercycler. Next generation sequencing was performed on the Illumina Hiseq 2500 or Novaseq at Illumina Novaseq 6000 system with paired-end 150bp reads.
Data analysis	Softwares used: ImageJ 1.51s, Graphpad Prism 8, Matlab R2017b, R v3.5.3, Python v2.7.13, FastQC v0.11.8, Tophat2 v2.0.4, Cufflinks v2.0.2, Cluster 3.0 (http://bonsai.hgc.jp/ ~mdehoon/software/cluster/software.htm), TreeView (http://jtreeview.sourceforge.net), DAVID v6.8.<br>R package used: ComBat (from sva v3.10.0), FactoMineR v1.30, Seurat v3.0, RankProd v2.42.0, pheatmap v1.0.10 .<br>Web service used: CIBERSORT (https:// cibersort.stanford.edu), g:profiler (https://biit.cs.ut.ee/gprofiler/gost), Google Charts (https:// developers.google.com/chart/interactive/docs/gallery/sankey).

For manuscripts utilizing custom algorithms or software that are central to the research but not yet described in published literature, software must be made available to editors and reviewers. We strongly encourage code deposition in a community repository (e.g. GitHub). See the Nature Portfolio guidelines for submitting code & software for further information.

Data

Policy information about availability of data

All manuscripts must include a data availability statement. This statement should provide the following information, where applicable:

- Accession codes, unique identifiers, or web links for publicly available datasets
- A description of any restrictions on data availability
- For clinical datasets or third party data, please ensure that the statement adheres to our policy

The RNA-seq data generated in this study were deposited in the NCBI Gene Expression Omnibus under accession number GSE171588. For the 10X Genomics data, raw and processed single-cell data can be downloaded following the instructions at https://github.com/ MarioniLab/EmbryoTimecourse2018. All other data are available from the corresponding authors upon request.

Human research participants

Policy information about studies involving human research participants and Sex and Gender in Research.

Reporting on sex and gender	N/A
Population characteristics	N/A
Recruitment	N/A
Ethics oversight	N/A

Note that full information on the approval of the study protocol must also be provided in the manuscript.

Field-specific reporting

Please select the one below that is the best fit for your research. If you are not sure, read the appropriate sections before making your selection.

☒ Life sciences ☐ Behavioural & social sciences ☐ Ecological, evolutionary & environmental sciences

For a reference copy of the document with all sections, see nature.com/documents/nr-reporting-summary-flat.pdf

Life sciences study design

All studies must disclose on these points even when the disclosure is negative.

Sample size	The sampling strategy was modified in accordance to the size of the section and specific research purpose. At least two embryo replicates for each developmental stages were performed and the correlation between these embryos was assessed. And the correlation coefficients were listed in the manuscript and figures. The sample size were determined based on our previous study (Nature volume 572, pages528–532 (2019); Nature Protocols volume 12, pages566–580 (2017)).
Data exclusions	No data were excluded. All replicates were included and only the representative data set were shown.
Replication	The consistency of the data has been clearly exhibited in the figures. For the Geo-seq samples, at least two embryo replicates for each stage were performed.
Randomization	All embryos for each embryonic stage subjected to Geo-seq in this study were chosen randomly. The embryos progression of gastrulation at the five developmental time-points were staged by the proximal-distal span of the primitive streak and anterior-posterior span of mesoderm layer (Development 118, 1255-1266 (1993))
Blinding	The embryos samples were collected at different embryonic stages by different people without knowing the outcome of the sequencing result.

Reporting for specific materials, systems and methods

We require information from authors about some types of materials, experimental systems and methods used in many studies. Here, indicate whether each material, system or method listed is relevant to your study. If you are not sure if a list item applies to your research, read the appropriate section before selecting a response.

Materials & experimental systems

n/a	Involved in the study
☒	☐ Antibodies
☒	☐ Eukaryotic cell lines
☒	☐ Palaeontology and archaeology
☐	☒ Animals and other organisms
☒	☐ Clinical data
☒	☐ Dual use research of concern

Methods

n/a	Involved in the study
☒	☐ ChIP-seq
☒	☐ Flow cytometry
☒	☐ MRI-based neuroimaging

Animals and other research organisms

Policy information about studies involving animals; ARRIVE guidelines recommended for reporting animal research, and Sex and Gender in Research

Laboratory animals	For Geo-seq analysis, whole-mount in situ hybridization, and ex vivo embryo culture, mus musculus C57BL/6 males and females at the age of 6-8 weeks were mated, and the embryos were collected at respective embryonic stages. For the generation of Pkd1l1, Dand5, Dnah11 mutant mouse embryos, pseudo-pregnant ICR female mice at the age of 10-12 weeks were used for two-cell injected embryos transferring. All animals were housed with a standard light/dark cycle and availability of food and water. The ambient temperatures was between 24~26°C with humidity between 50%~60%.
Wild animals	This study did not involve wild animals.
Reporting on sex	This information has not been collected.
Field-collected samples	This study did not involve animals collected from the field.
Ethics oversight	All animal experiments were performed in compliance with the guidelines of the Animal Ethical Committee of the CAS Center for Excellence in Molecular Cell Science, Chinese Academy of Sciences.

Note that full information on the approval of the study protocol must also be provided in the manuscript.
